# Supplementary material for: CNNM proteins selectively bind to the TRPM7 channel to stimulate divalent cation entry into cells
Source: PLoS Biol. 2021 Dec 20;19(12):e3001496. doi: 10.1371/journal.pbio.3001496 (PMC8726484; doi:10.1371/journal.pbio.3001496)

Fig. 1A_1
Normal Mg^{2+}

1. HA-TRPM7; Flag-CNNM1
2. HA-TRPM7; Flag-CNNM2
3. HA-TRPM7; Flag-CNNM3
4. HA-TRPM7; Flag-CNNM4
5. Flag-CNNM1
6. Flag-CNNM2
7. Flag-CNNM3
8. Flag-CNNM4

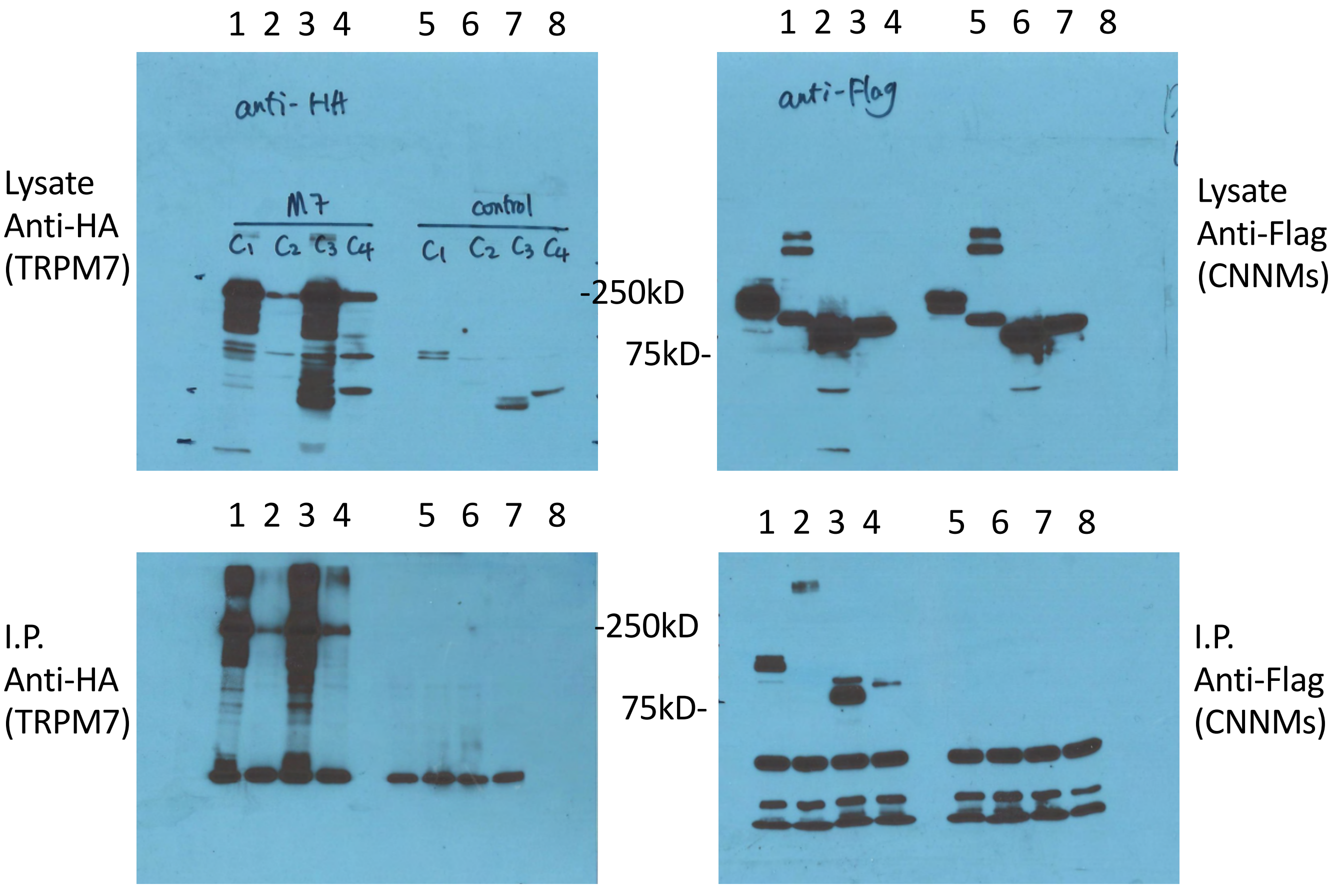

Fig. 1A_2

20mM Mg^{2+}

1. HA-TRPM7; Flag-CNNM1
2. HA-TRPM7; Flag-CNNM2
3. HA-TRPM7; Flag-CNNM3
4. HA-TRPM7; Flag-CNNM4
5. Flag-CNNM1
6. Flag-CNNM2
7. Flag-CNNM3
8. Flag-CNNM4

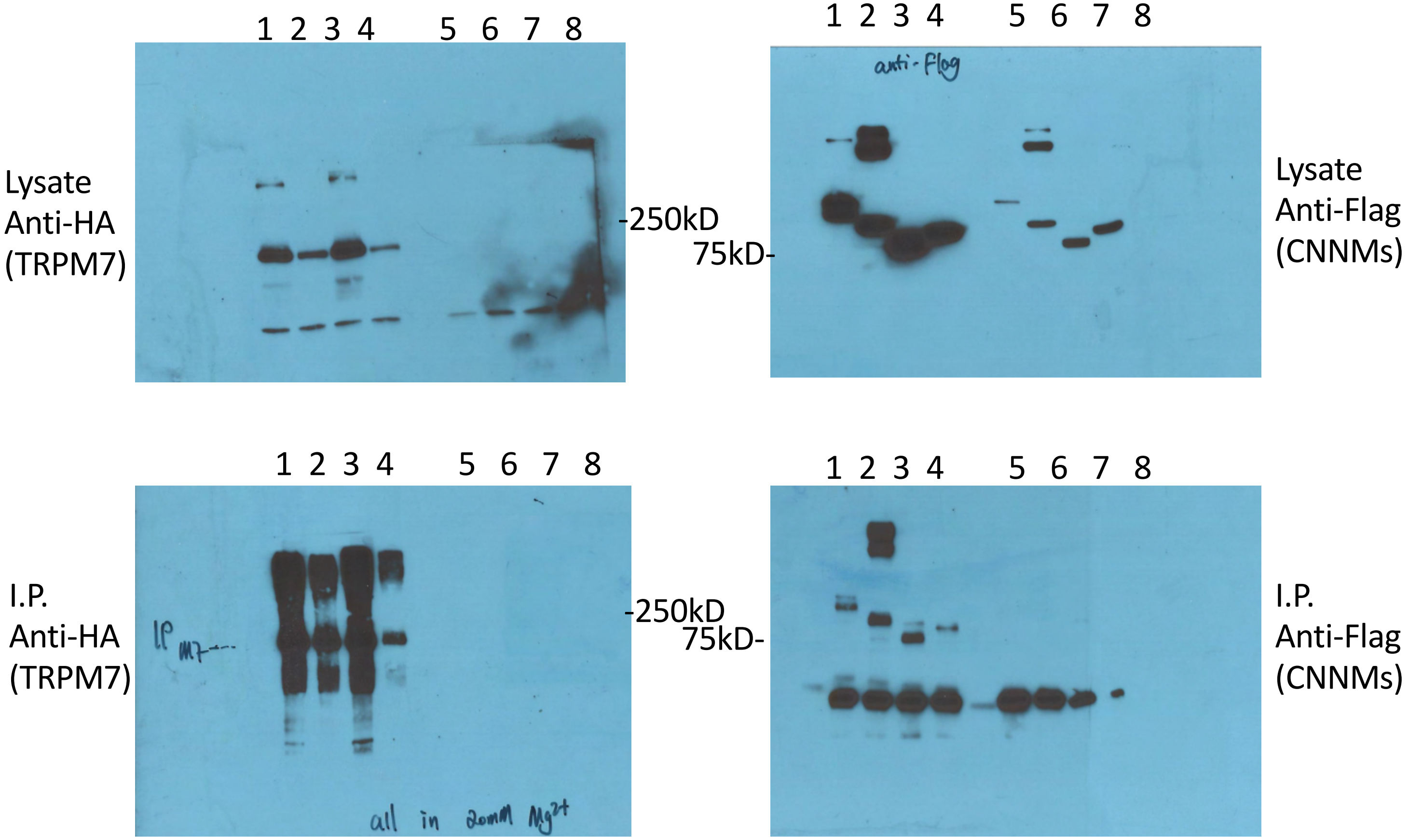

Fig. 1B

1. Lysate No Transfection
2. Lysate Flag-TRPM2
3. Lysate Flag-TRPM7
4. I.P. No Transfection
5. I.P. Flag-TRPM2
6. I.P. Flag-TRPM7

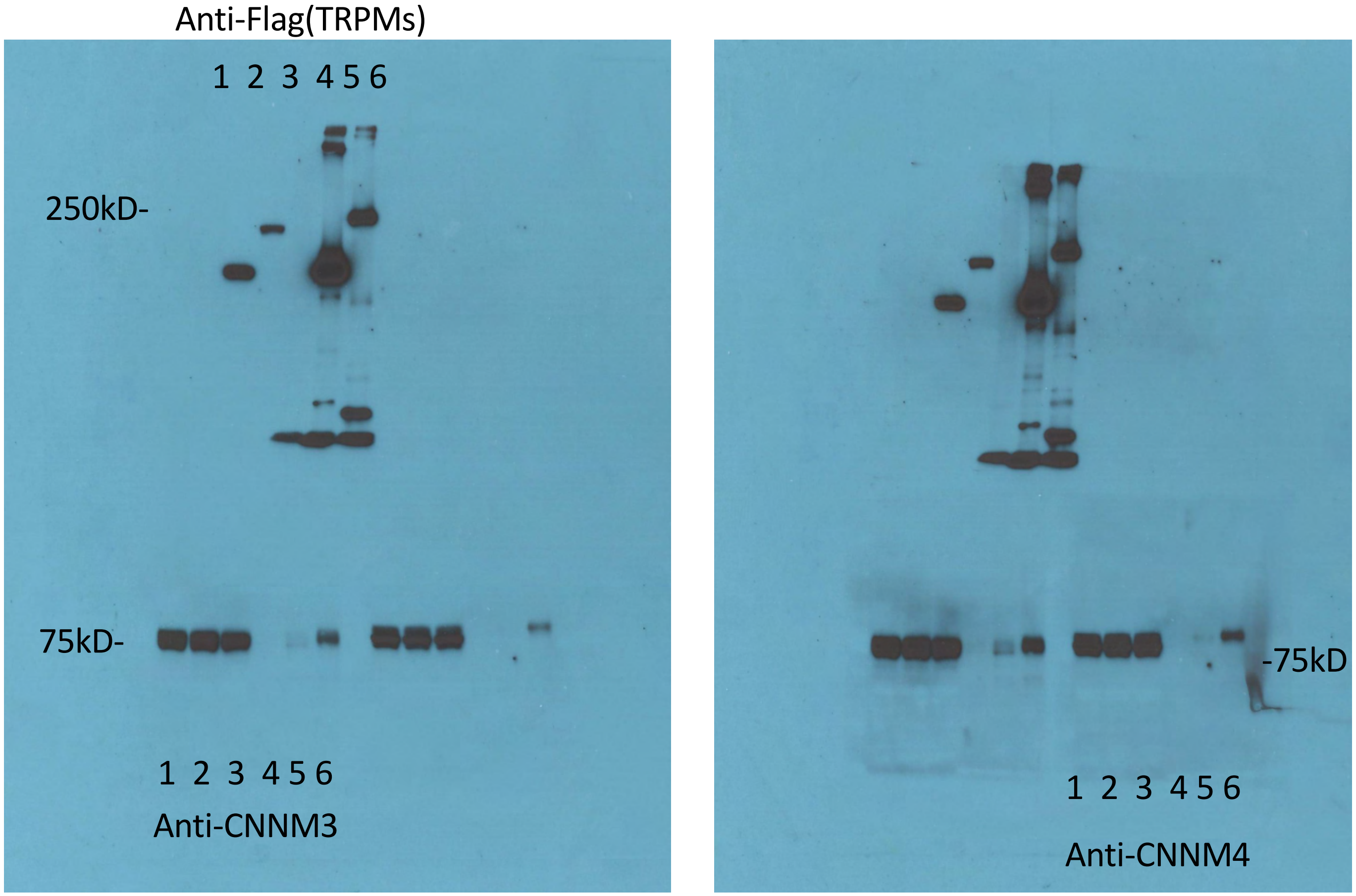

Fig. 1E

1. 293-M7
2. 293-M7; CNNM1
3. 293-M7; CNNM2
4. 293-M7; CNNM3
5. 293-M7; CNNM4

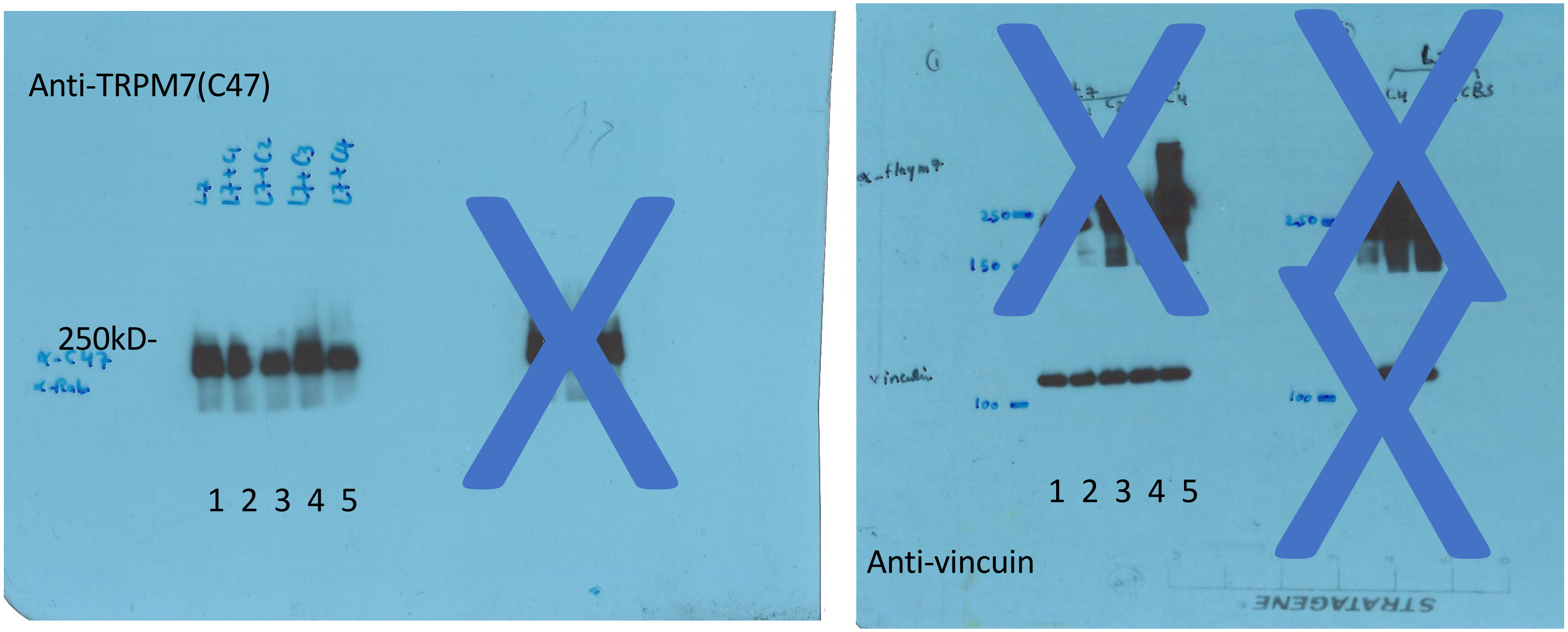

Fig. 2D_1

anti_CNNM3

1 2 3 4 5 6 7 8

75kd

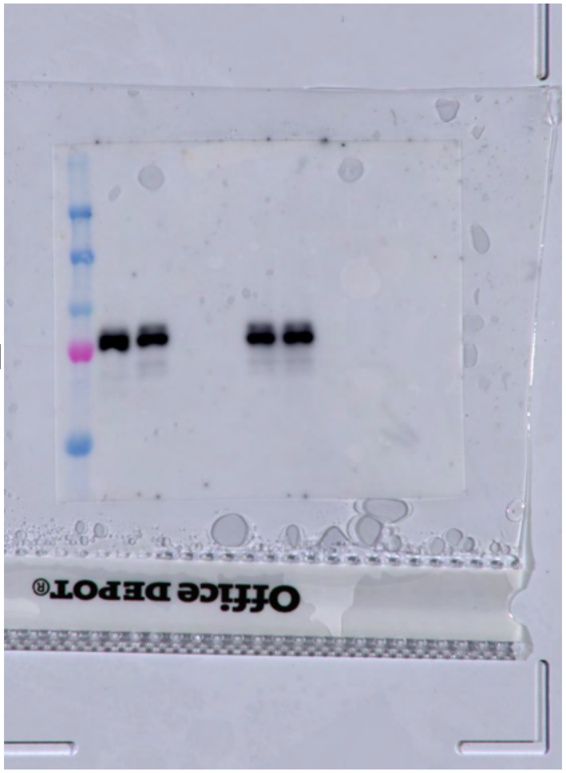

1. 293
2. 293-M7
3. 293-M7; ΔCNNM3 -1
4. 293-M7; ΔCNNM3 -2
5. 293-M7; ΔCNNM4 -1
6. 293-M7; ΔCNNM4 -2
7. 293-M7; ΔCNNM3; ΔCNNM4 -1
8. 293-M7; ΔCNNM3; ΔCNNM4 -2

anti_CNNM4

1 2 3 4 5 6 7 8

75kd

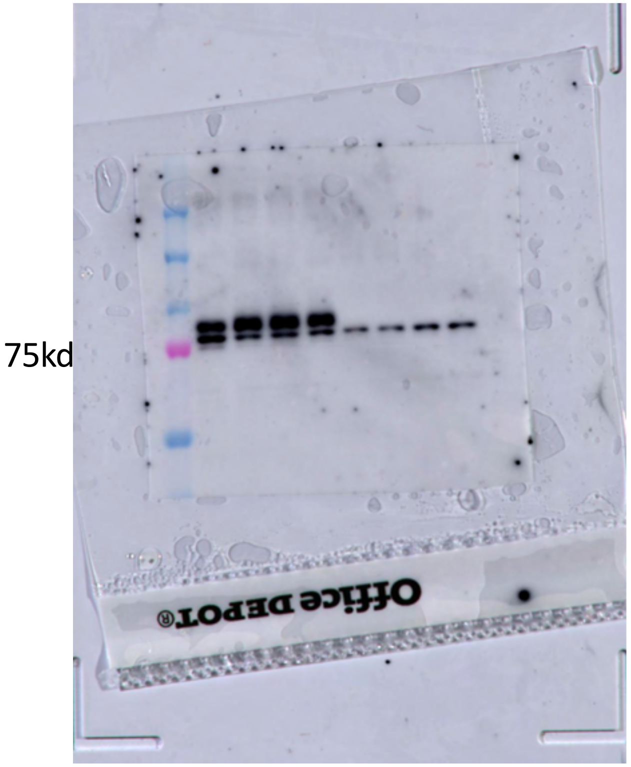

Fig. 2D_2

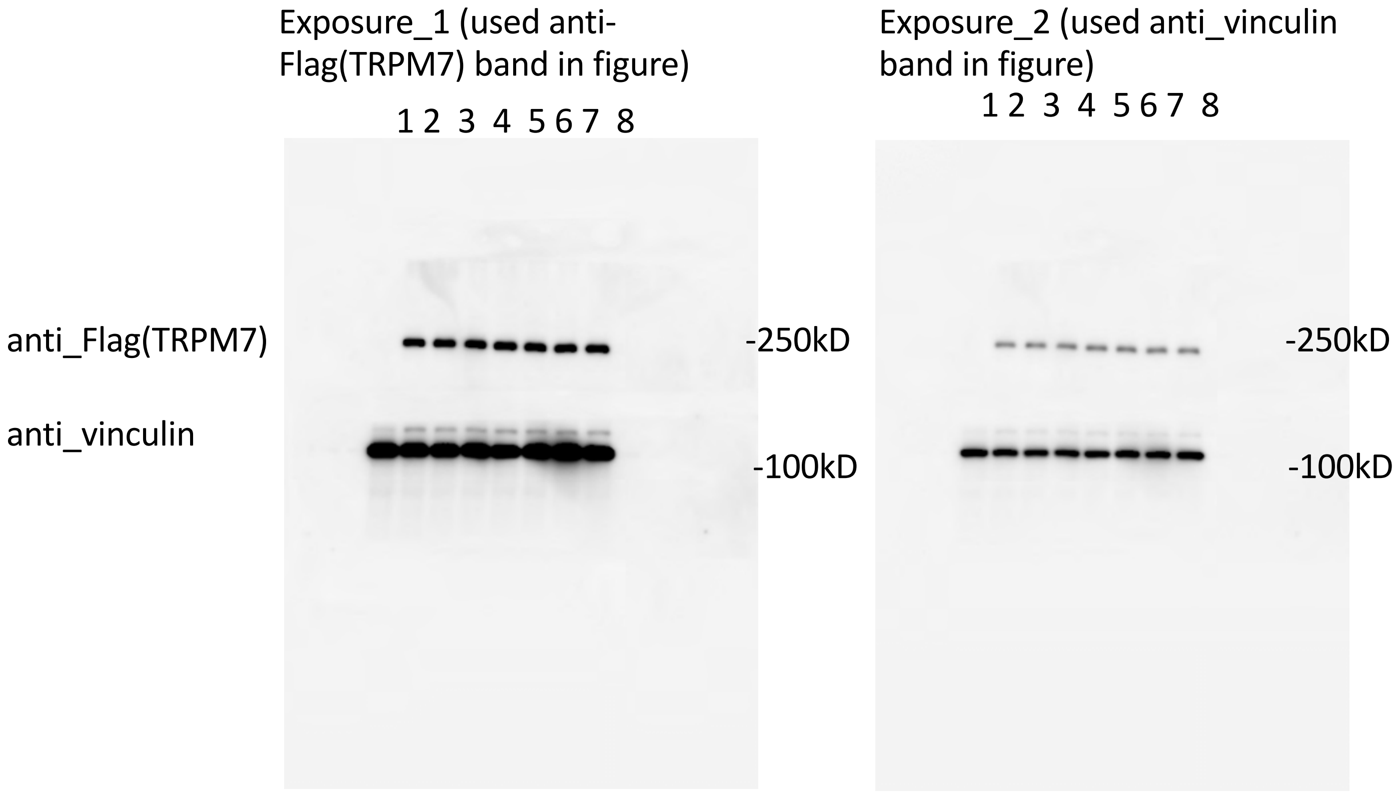

1. 293
2. 293-M7
3. 293-M7; ΔCNNM3 -1
4. 293-M7; ΔCNNM3 -2
5. 293-M7; ΔCNNM4 -1
6. 293-M7; ΔCNNM4 -2
7. 293-M7; ΔCNNM3; ΔCNNM4 -1
8. 293-M7; ΔCNNM3; ΔCNNM4 -2

Fig. 2G_1

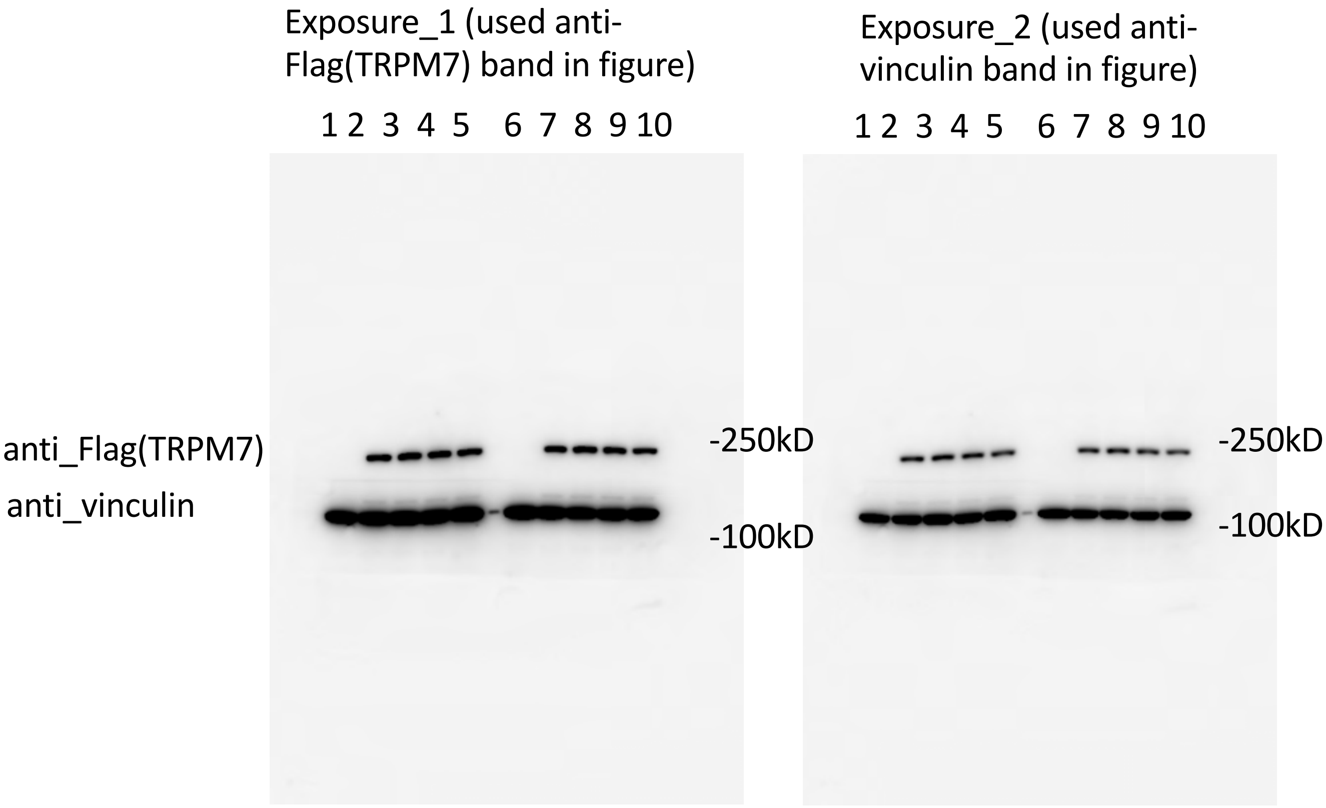

1. 293
2. 293-M7
3. 293-M7; ΔCNNM3
4. 293-M7; ΔCNNM4
5. 293-M7; ΔCNNM3; ΔCNNM4
6. 293; PRL-2
7. 293-M7; PRL-2
8. 293-M7; ΔCNNM3; PRL-2
9. 293-M7; ΔCNNM4; PRL-2
10. 293-M7; ΔCNNM3; ΔCNNM4: PRL-2

Fig. 2G_2

used anti-Flag(PRL-2) band in figure

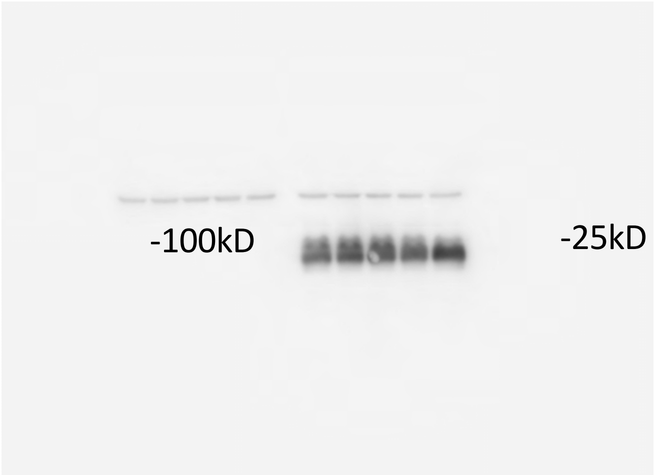

1. 293
2. 293-M7
3. 293-M7; ΔCNNM3
4. 293-M7; ΔCNNM4
5. 293-M7; ΔCNNM3; ΔCNNM4
6. 293; PRL-2
7. 293-M7; PRL-2
8. 293-M7; ΔCNNM3; PRL-2
9. 293-M7; ΔCNNM4; PRL-2
10. 293-M7; ΔCNNM3; ΔCNNM4: PRL-2

Fig. 2E

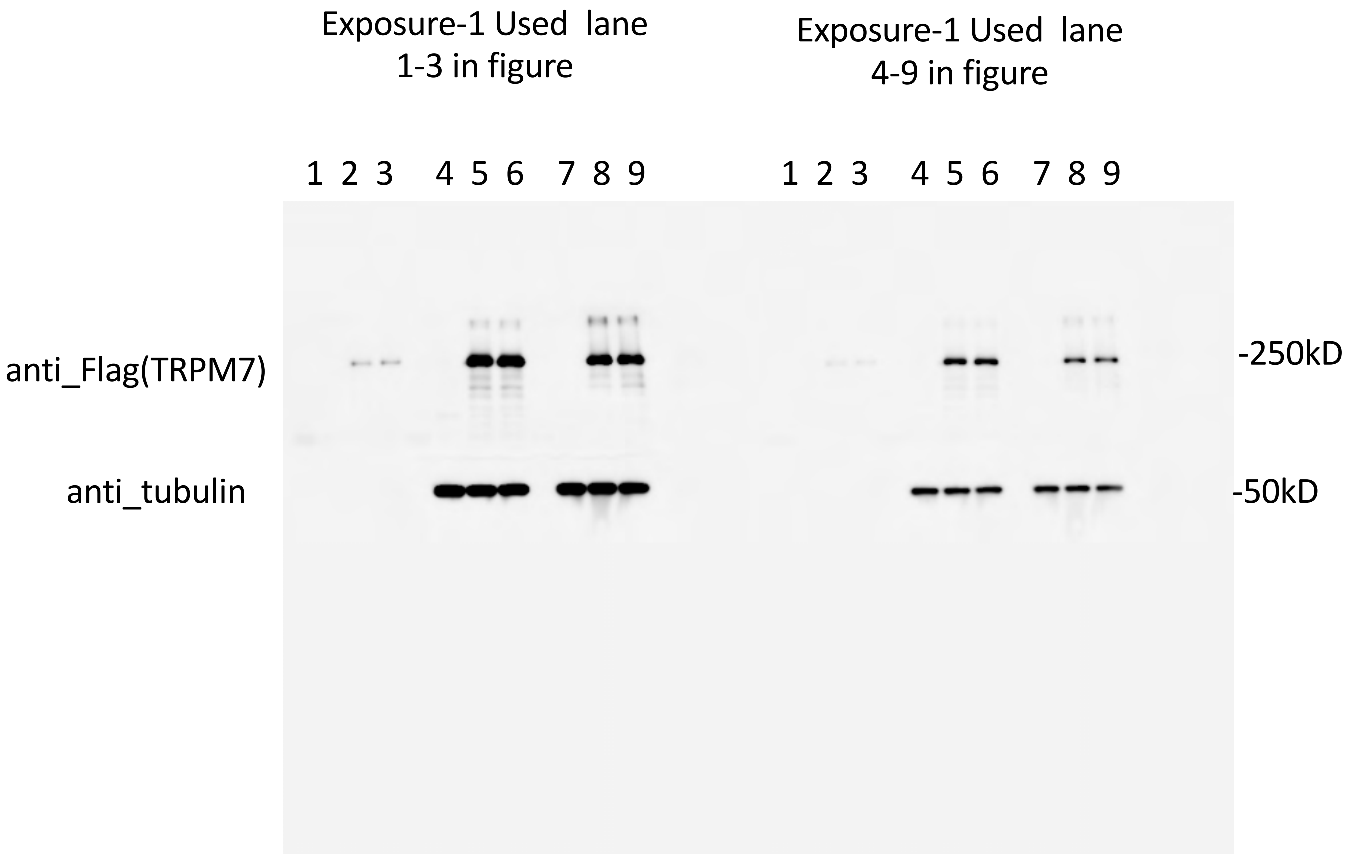

1. SA Purified 293
2. SA Purified 293-M7
3. SA Purified 293-M7; ΔC3; ΔC4
4. Input 293
5. Input 293-M7
6. Input 293-M7; ΔC3; ΔC4
7. SA unbound
8. SA unbound 293-M7
9. SA unbound 293-M7; ΔC3; ΔC4

Fig. 4A

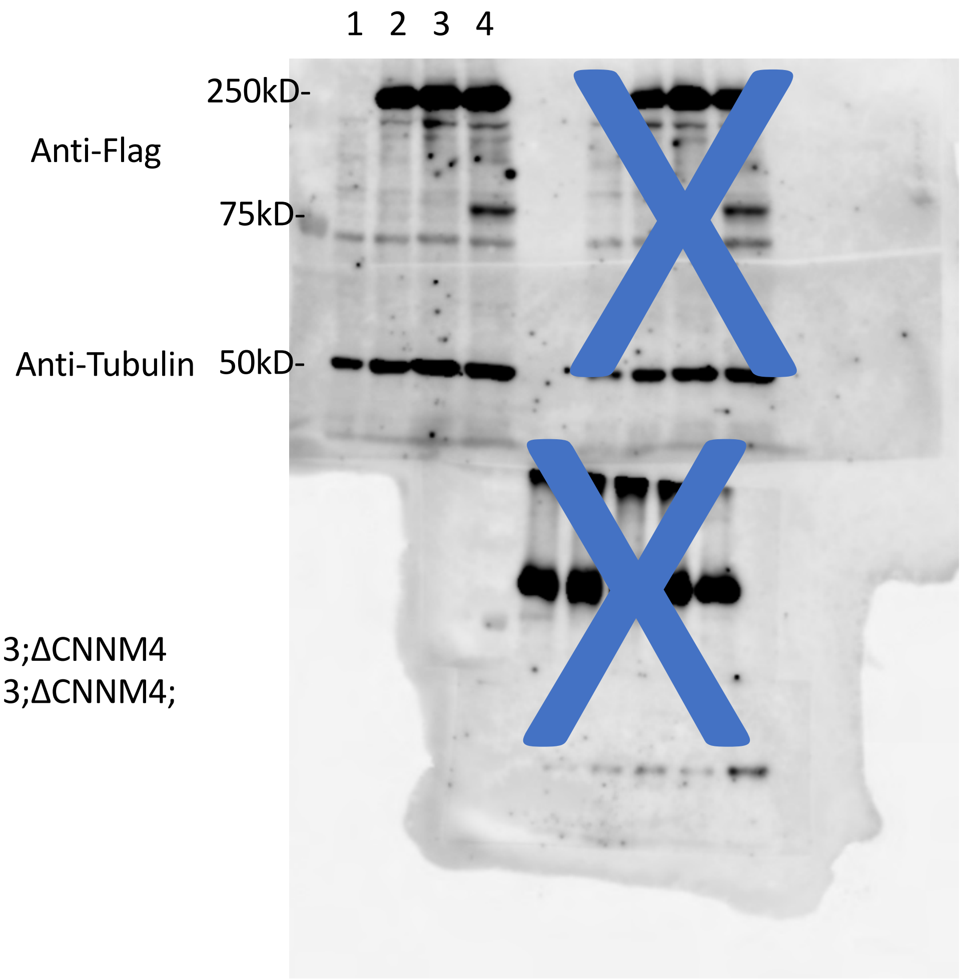

1. 293
2. 293-M7
3. 293-M7; ΔCNNM3;ΔCNNM4
4. 293-M7; ΔCNNM3;ΔCNNM4; Cumate-CNNM4

S1 Fig

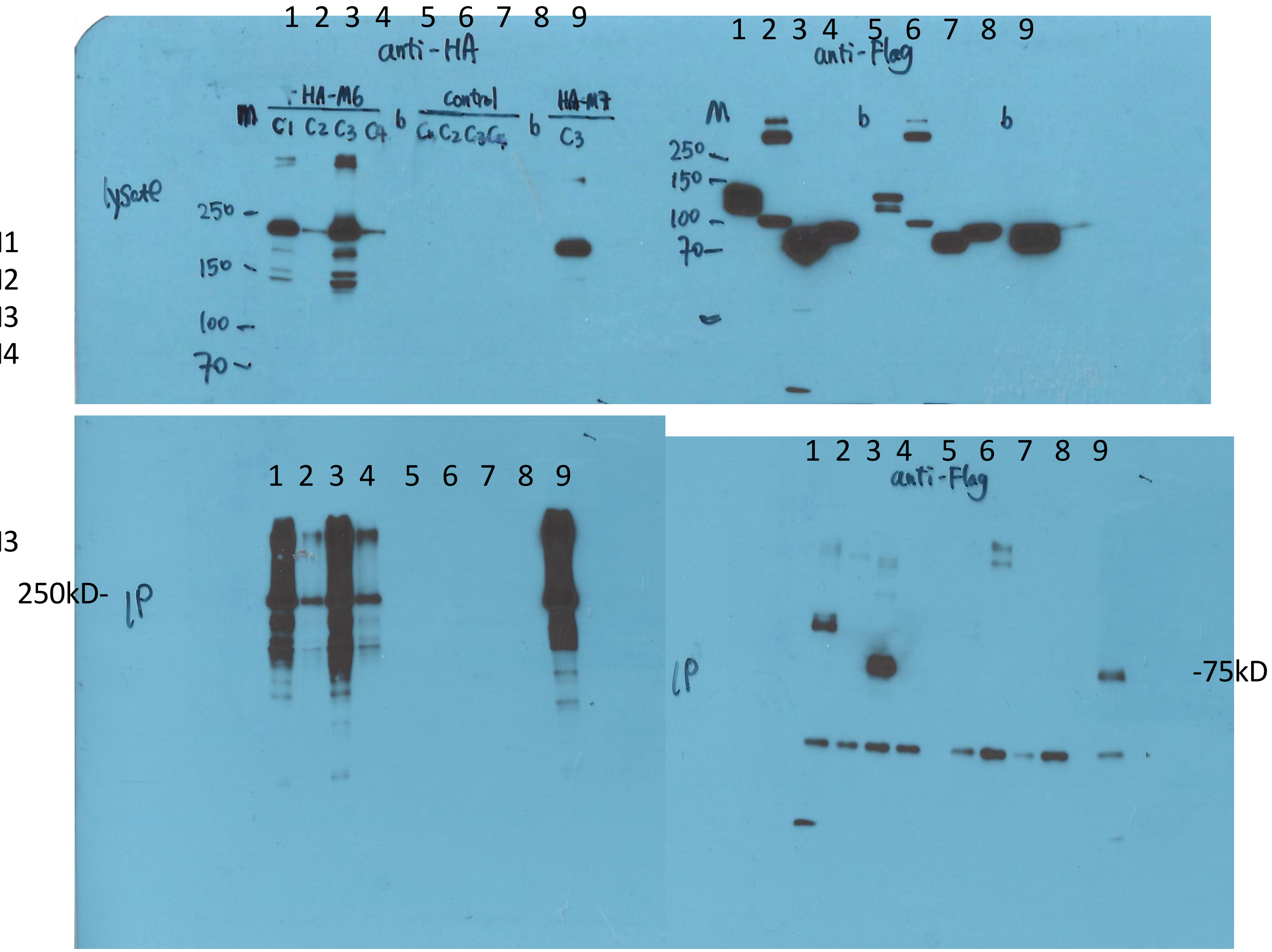

1. HA-M6; Flag-CNNM1
2. HA-M6; Flag-CNNM2
3. HA-M6; Flag-CNNM3
4. HA-M6; Flag-CNNM4
5. Flag-CNNM1
6. Flag-CNNM2
7. Flag-CNNM3
8. Flag-CNNM4
9. HA-M7; Flag-CNNM3

S3A Fig (Hela Cells)

1. Lysate control
2. Lysate HA-M7
3. I.P. control
4. I.P. HA-M7

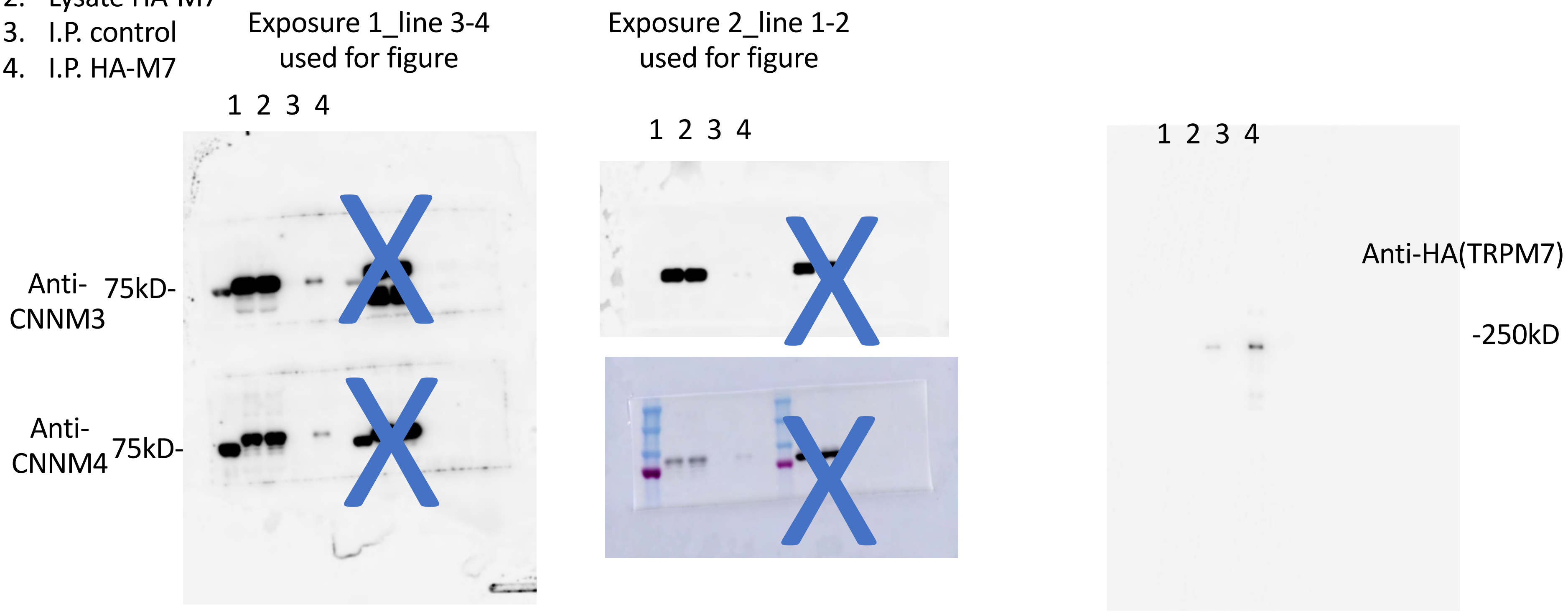

S3B Fig(OK Cells)

1. Lysate control
2. Lysate HA-M7
3. I.P. control
4. I.P. HA-M7

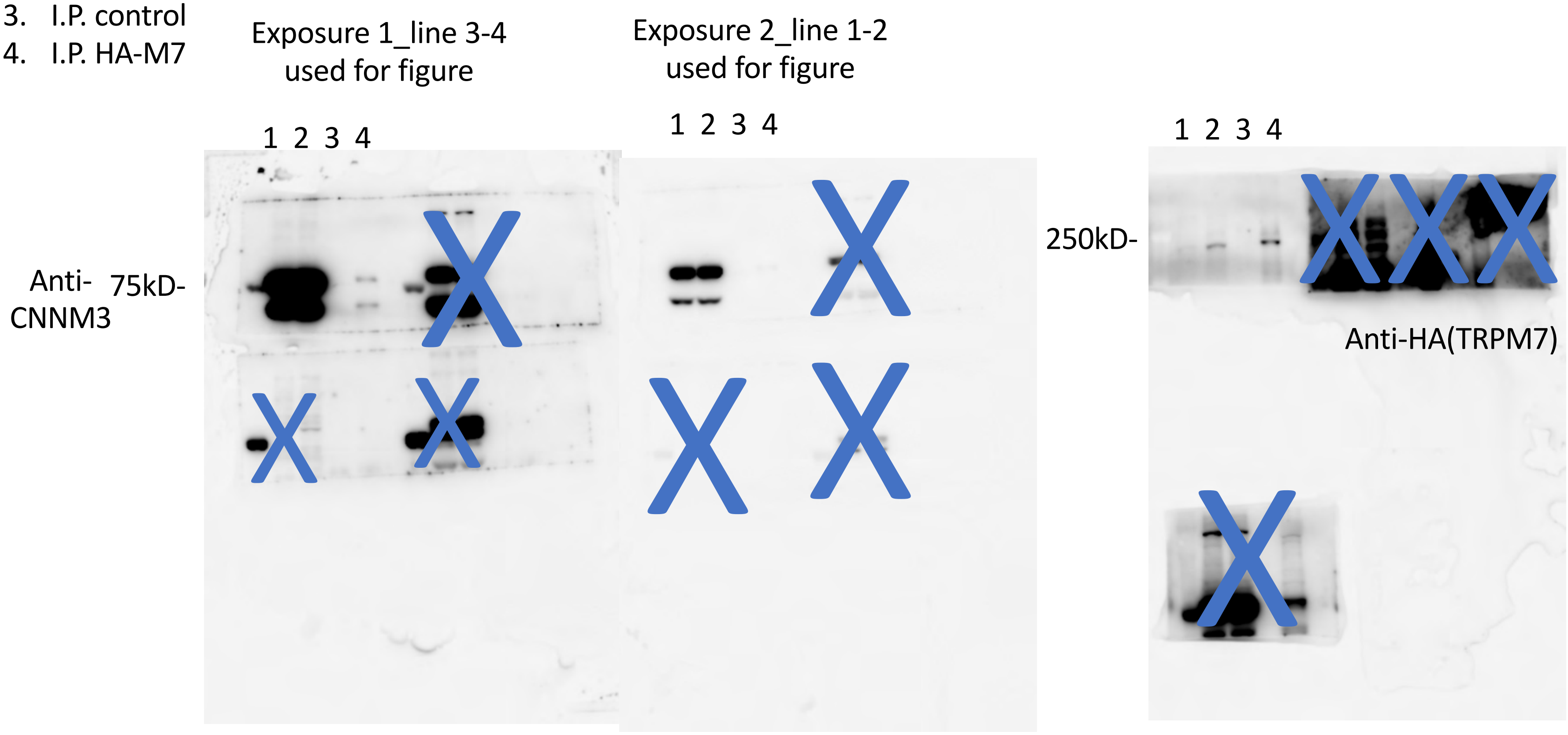

S3C Fig (HAP1 Cells)

1. Lysate control
2. Lysate HA-M7
3. I.P. control
4. I.P. HA-M7

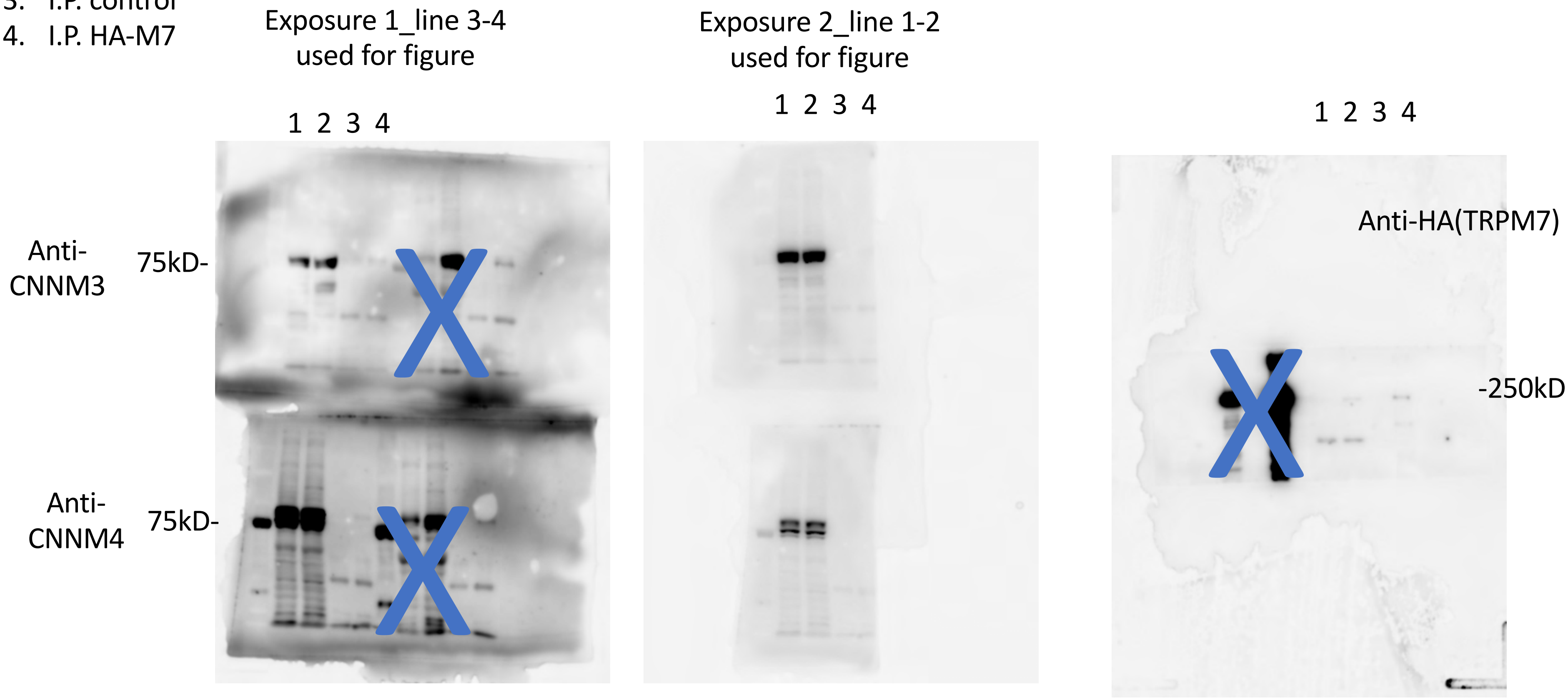

S3D Fig(RPTEC Cells)

1. Lysate control
2. Lysate HA-M7
3. I.P. control
4. I.P. HA-M7

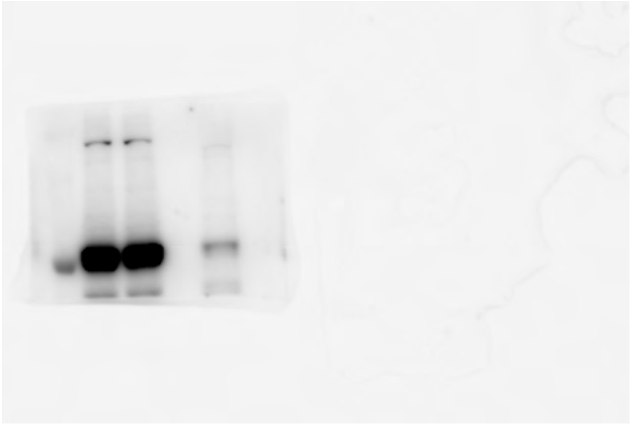

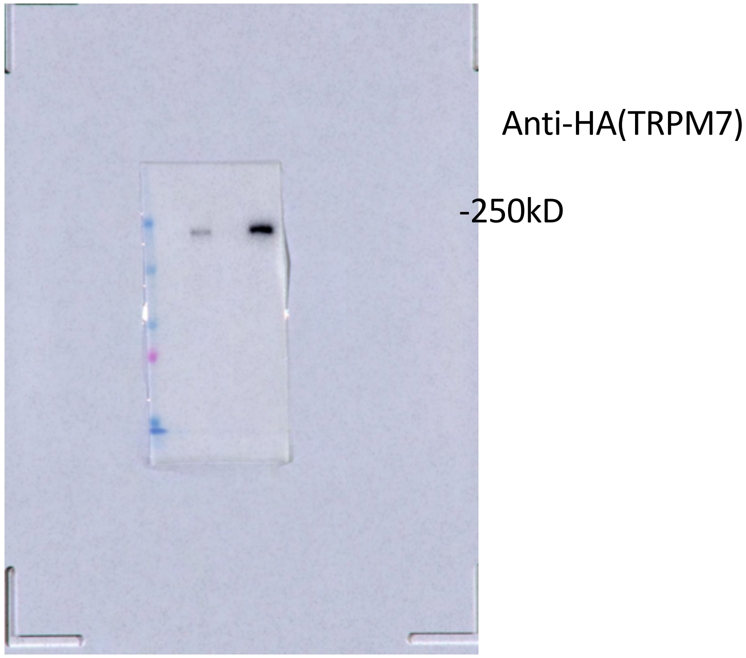

S3E Fig (ZR-75-1 Cells)

1. Lysate (anti-myc)
2. Lysate (anti-TRPM7)
3. I.P. anti-myc
4. I.P. anti-TRPM7
5. Lysis buffer anti-myc
6. Lysis buffer anti-TRPM&

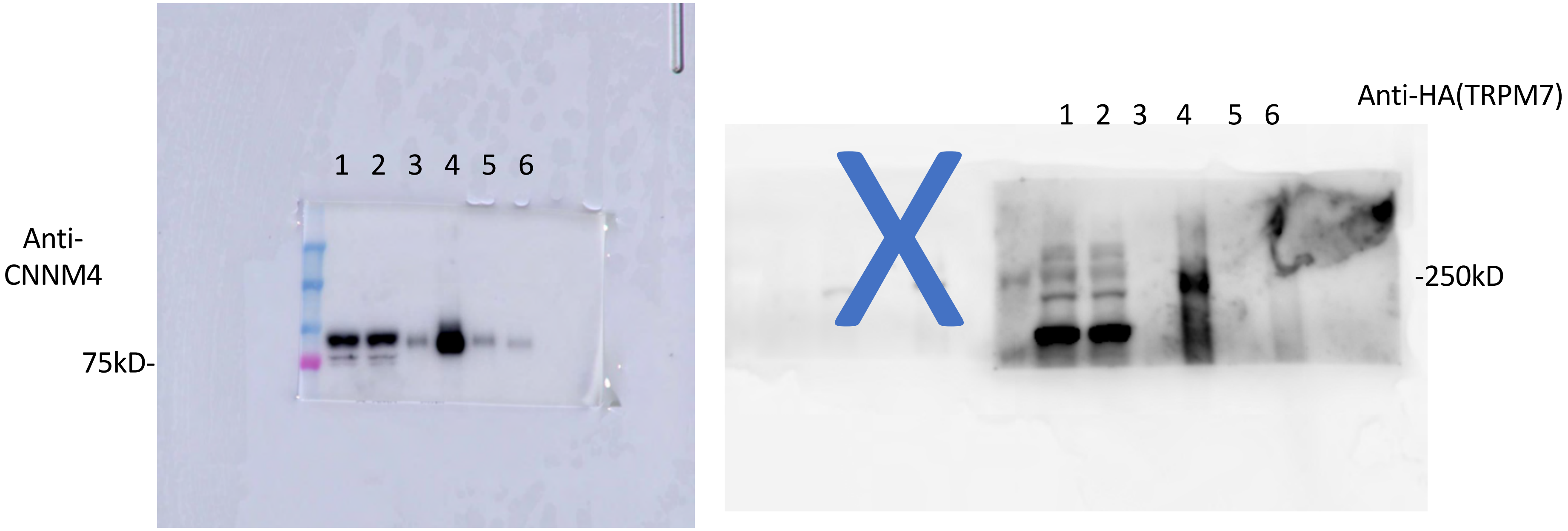

S5 Fig

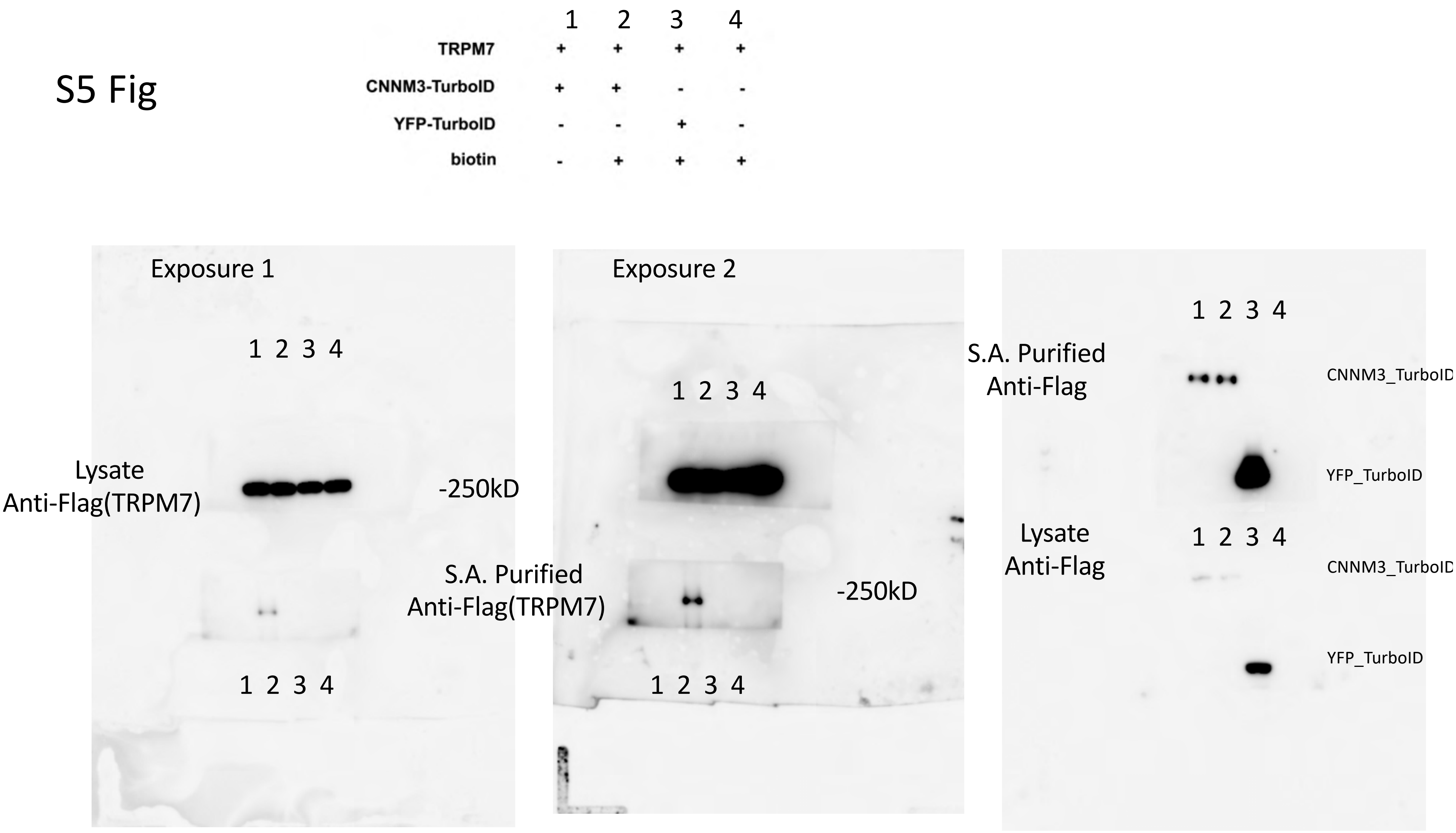

1 2 3 4
TRPM7 + + + +
CNNM3-TurboID + + - -
YFP-TurboID - - + -
biotin - + + +
Exposure 1
1 2 3 4
Lysate
Anti-Flag(TRPM7)
-250kD
1 2 3 4
Exposure 2
1 2 3 4
S.A. Purified
Anti-Flag(TRPM7)
-250kD
1 2 3 4
1 2 3 4
S.A. Purified
Anti-Flag
CNNM3_TurboID
YFP_TurboID
Lysate
Anti-Flag
1 2 3 4
CNNM3_TurboID
YFP_TurboID

S6C Fig

1. 293T No Transfection
2. 293T CNNM2
3. 293T(ΔM7) No Transfection
4. 293T(ΔM7) CNNM2

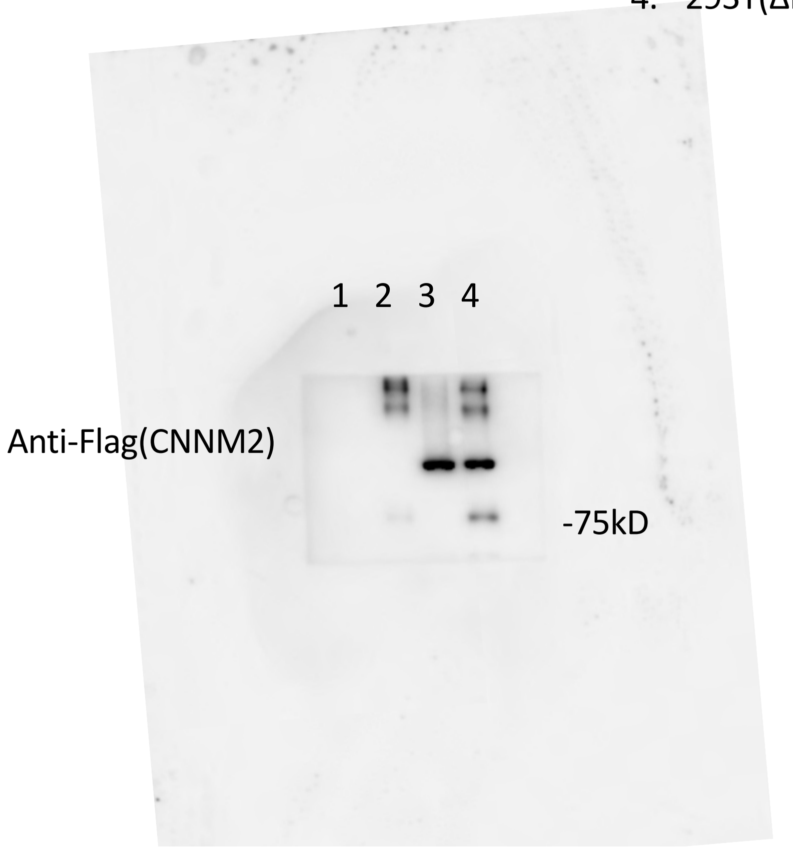

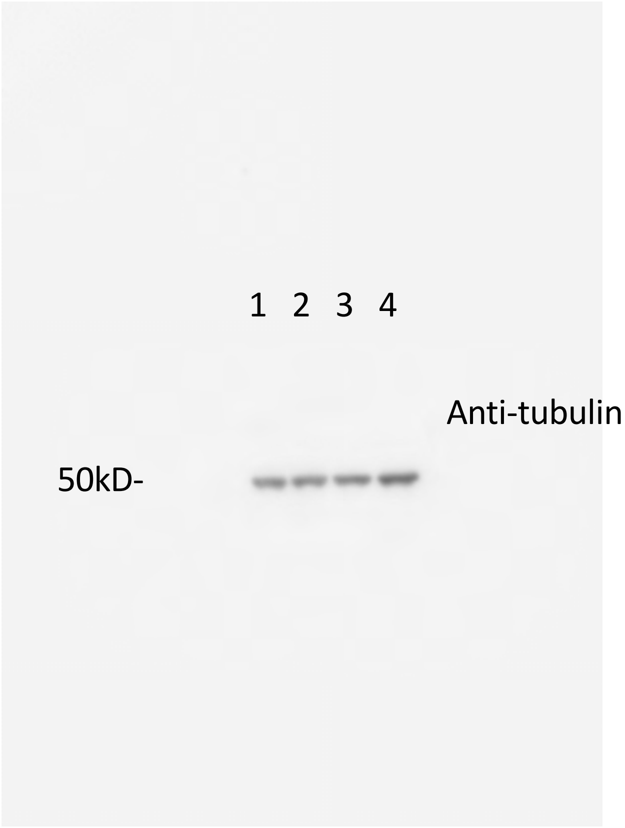

S6G Fig

1. 293 CNNM2
2. 293-M7(WT) tet+ 14h CNNM2
3. 293-M7(WT) tet+ 18h CNNM2
4. 293-M7(WT) tet+ 24h CNNM2
5. 293-M7(E1047K) tet+24h CNNM2

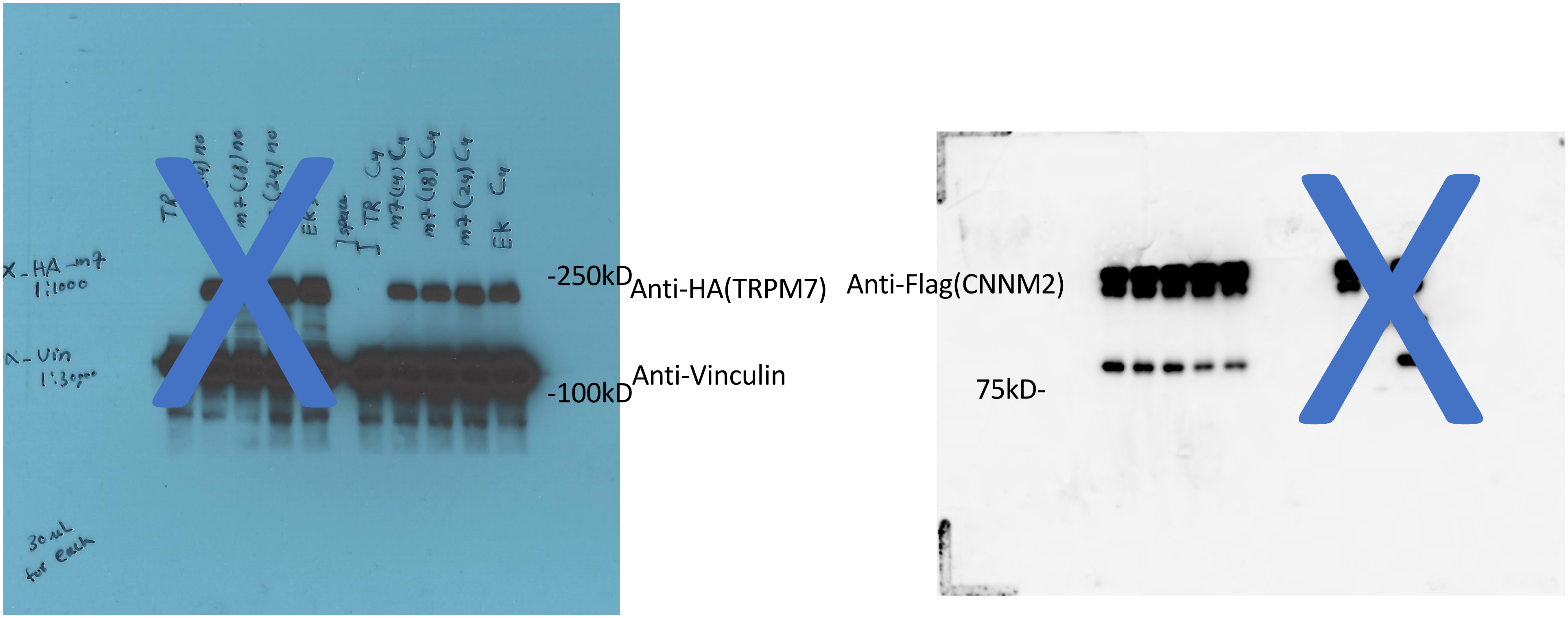

S8E_1 Fig

1. 293
2. 293-M7
3. 293-M7; CNNM4
4. 293-M7; SLC41A1

1 2 3 4

Anti-Flag(TRPM7)

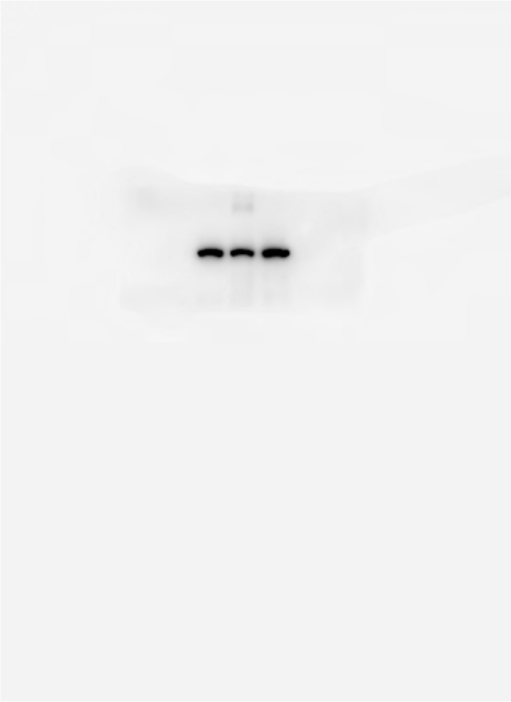

-250kD

100kD-

1 2 3 4

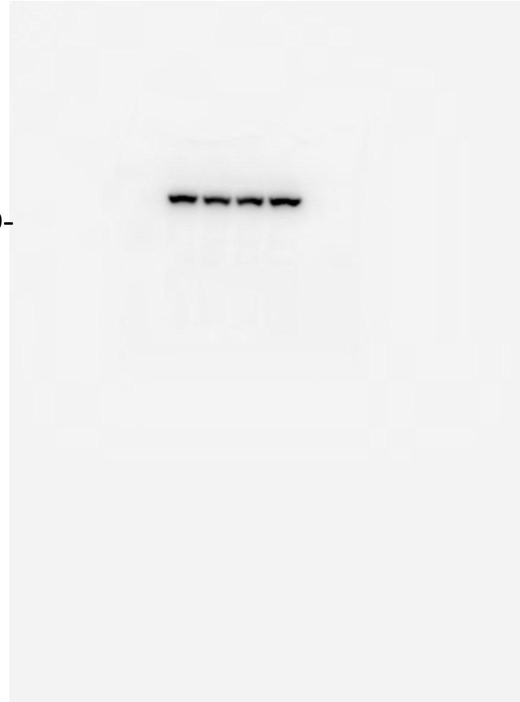

Anti-Vinculin

S8E_2 Fig

1. 293
2. 293-M7
3. 293-M7; CNNM4
4. 293-M7; SLC41A1

1 2 3 4

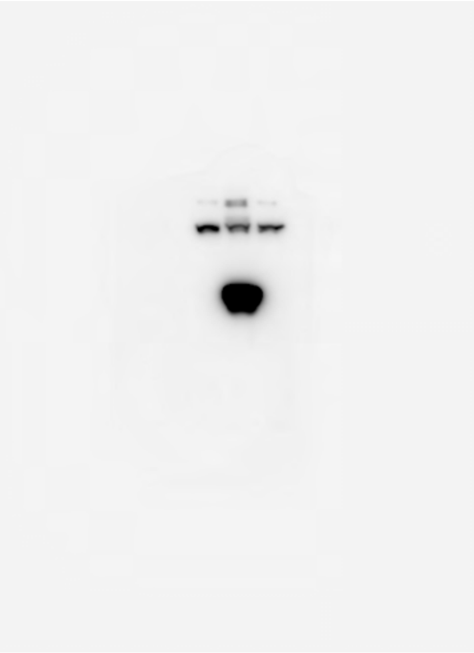

Anti-Flag(CNNM4)

-75kD

The same blot was used for anti-Flag first, then used for anti-HA without stripping.

50kD-

1 2 3 4

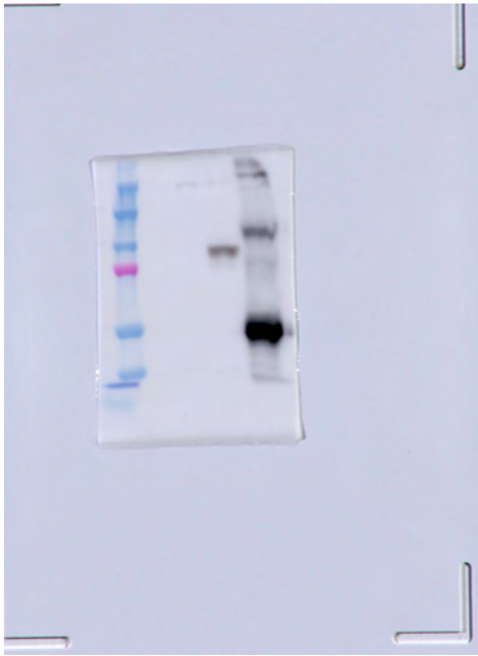

Anti-HA(SLC41A1)

S10C Fig

1. 293-M7 No Transfection
2. 293-M7 PRL-1
3. 293-M7 PRL-2
4. 293-M7 PRL-3

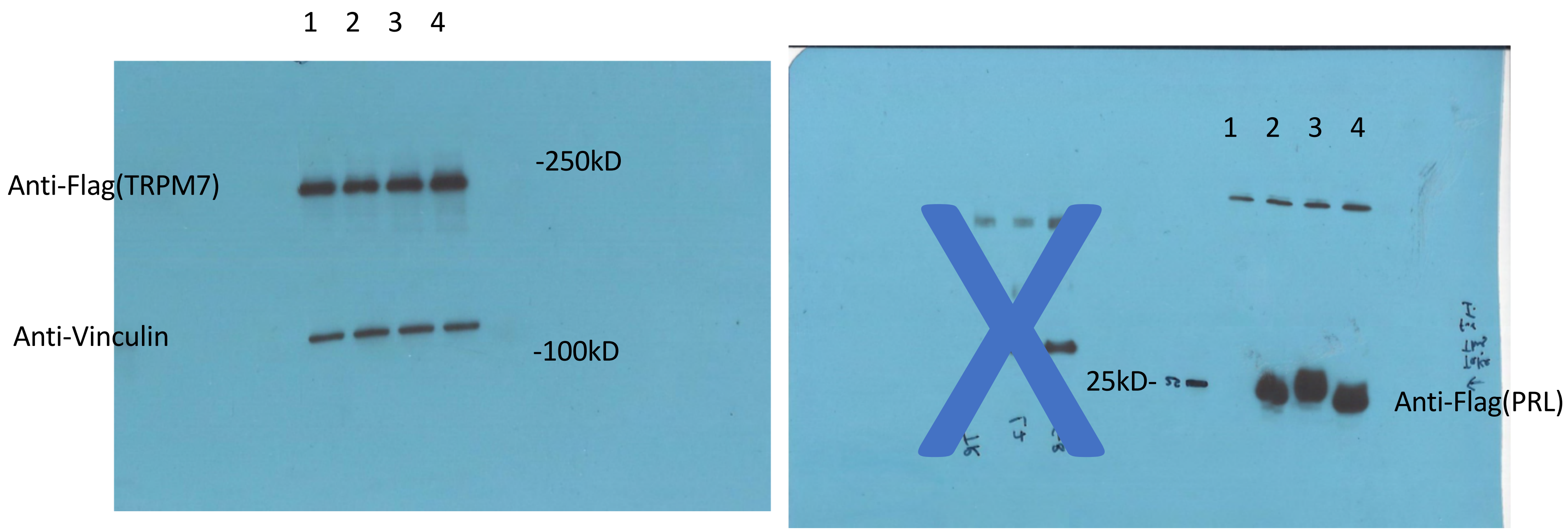

S10G Fig

1 2 3 4 5 6

Anti-Flag(PRL)

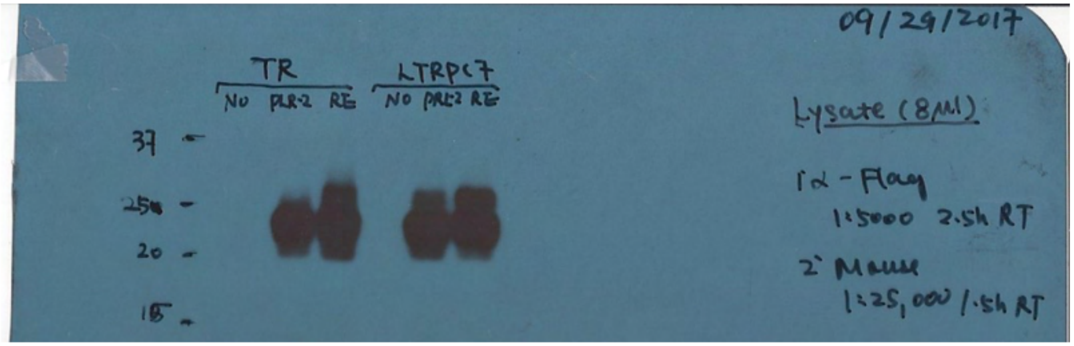

1. 293 No Transfection
2. 293 PRL-2(WT)
3. 293 PRL-2(RE)
4. 293-M7 No Transfection
5. 293-M7 PRL-2(WT)
6. 293-M7 PRL-2(RE)

1 2 3 4 5 6

Anti-TRPM7(C47)

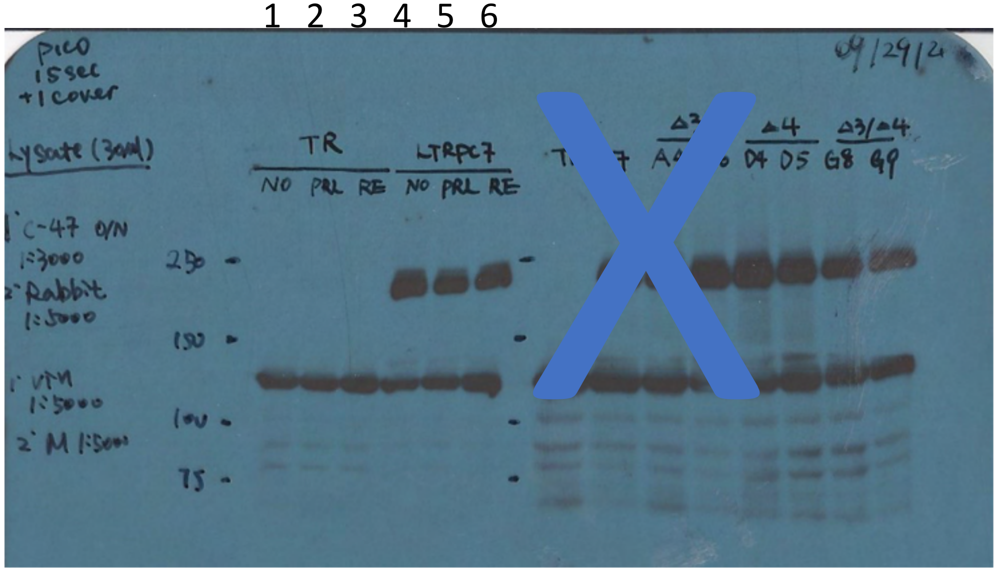

S11D Fig

1. 293-M7; no transfection
2. 293-M7; CNNM2(WT)
3. 293-M7; CNNM2(G356A)
4. 293-M7; CNNM2(E357A)
5. 293-M7; CNNM2(ΔCBS)
6. 293-M7; CNNM2(T568I)

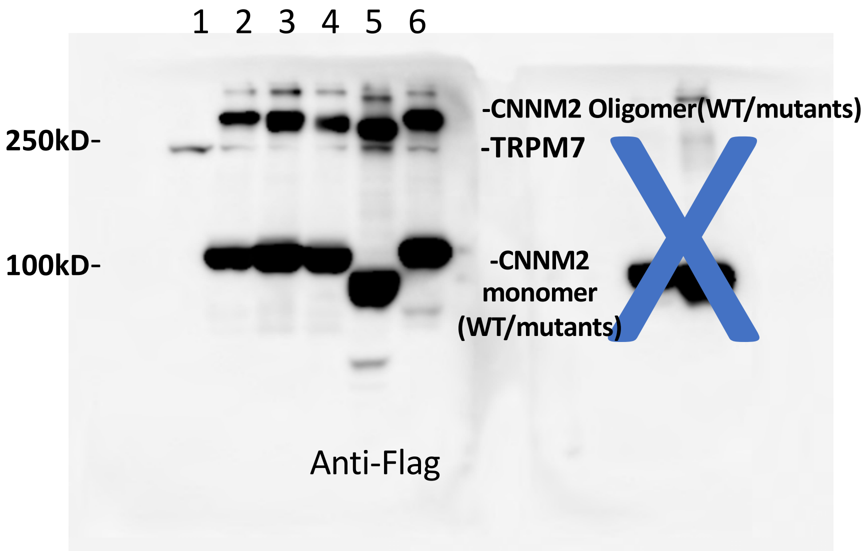

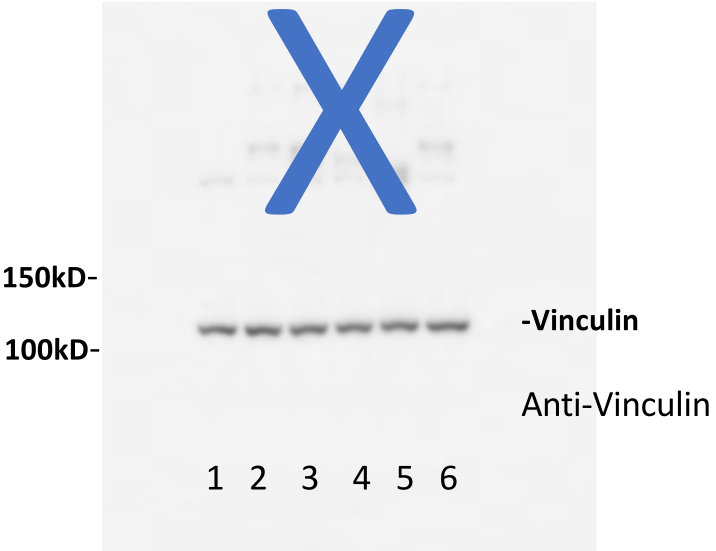

S11I Fig

1. 293-M7; no transfection
2. 293-M7; CNNM4(WT)
3. 293-M7; CNNM4(S196P)
4. 293-M7; CNNM4(S200Y)
5. 293-M7; CNNM4(N250A)
6. 293-M7; CNNM4(ΔCBS)
7. 293-M7; CNNM4(ΔCNBH)
8. 293-M7; CNNM4(F631K)

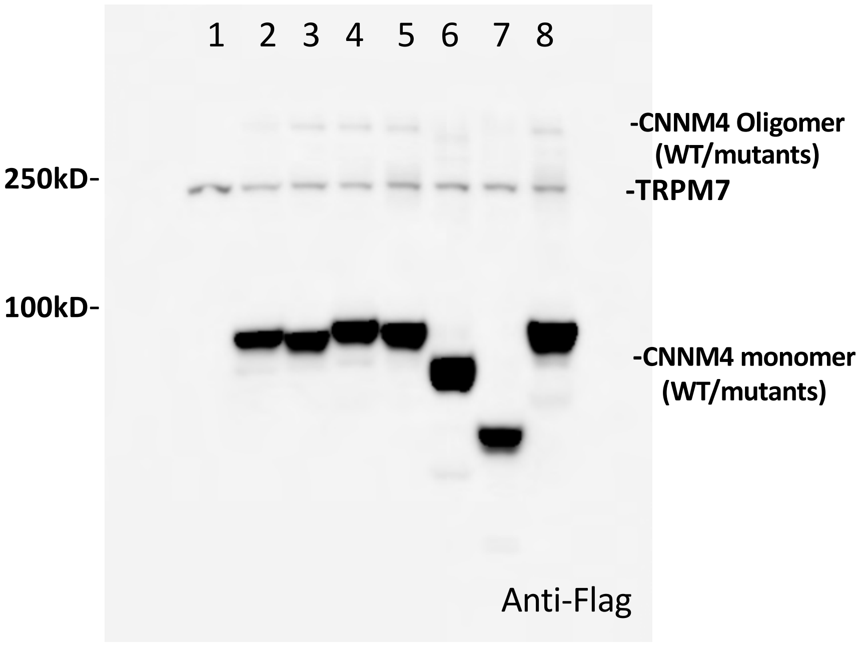

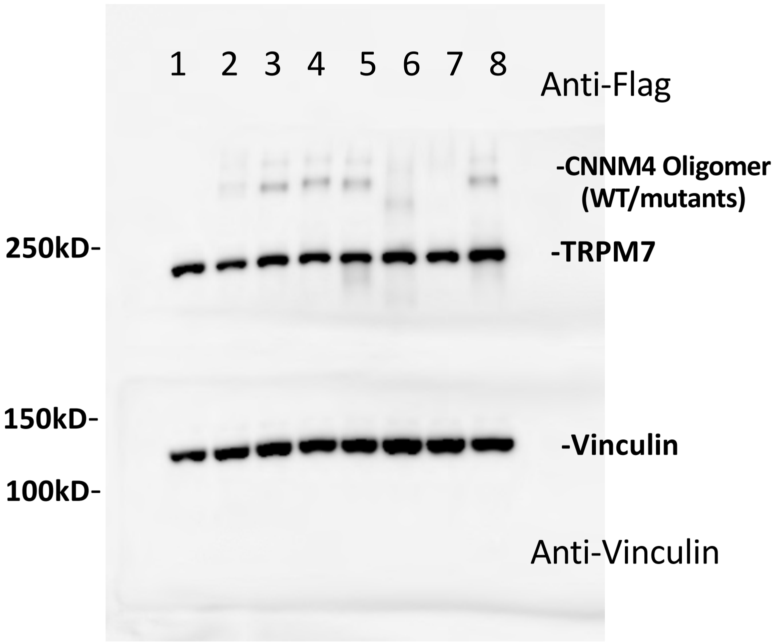

S12E Fig

1. 293-M7; control siRNA
2. 293-M7; control siRNA
3. 293-M7; arl-15 siRNA-1
4. 293-M7; arl-15 siRNA-1
5. 293-M7; arl-15 siRNA-2
6. 293-M7; arl-15 siRNA-2

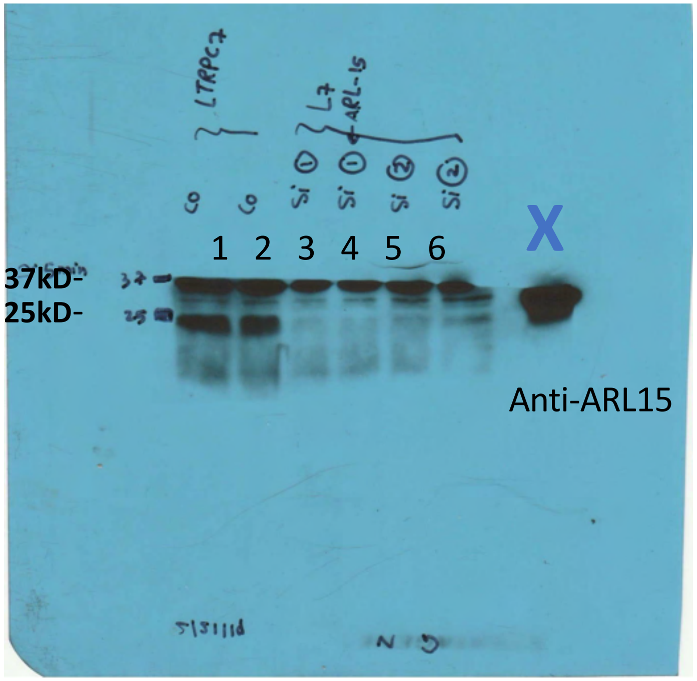

S16A Fig

1. Lysate. No Transfection
2. Lysate. Flag-CNNM1
3. Lysate. Flag-CNNM2
4. I.P. No Transfection
5. I.P. Flag-CNNM1
6. I.P. Flag-CNNM2

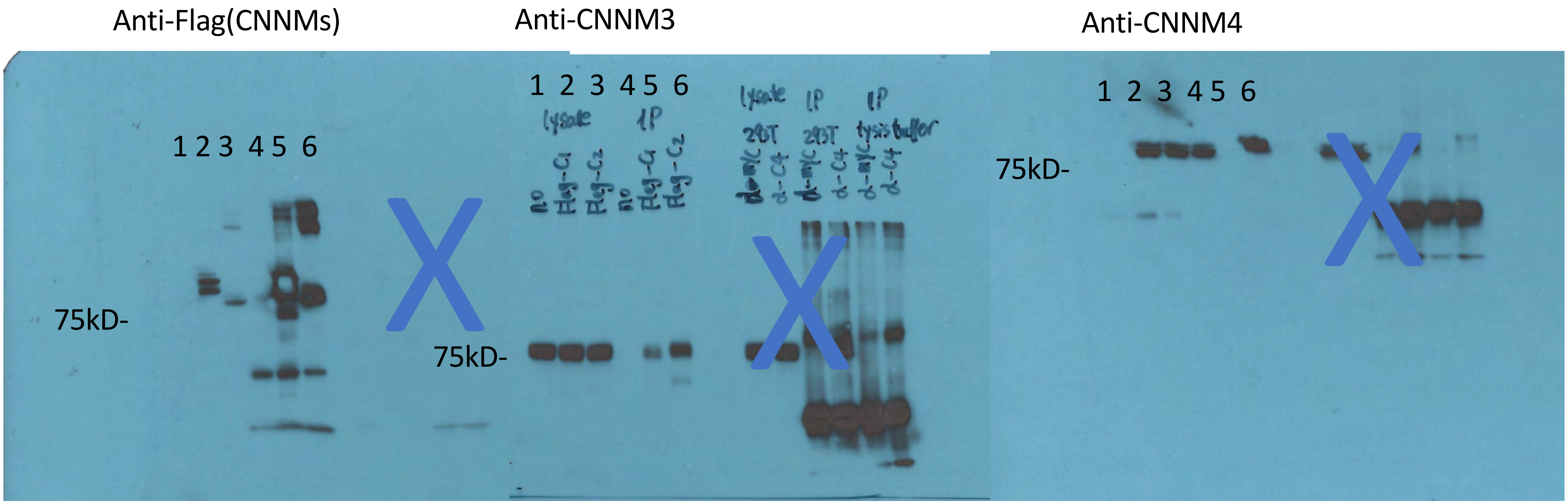

S16B Fig

1. Lysate. No Transfection
2. Lysate. Flag-CNNM3
3. Lysate. Flag-CNNM4
4. I.P. No Transfection
5. I.P. Flag-CNNM3
6. I.P. Flag-CNNM4

Anti-Flag(CNNMs)

1 2 3 4 5 6

75kD-

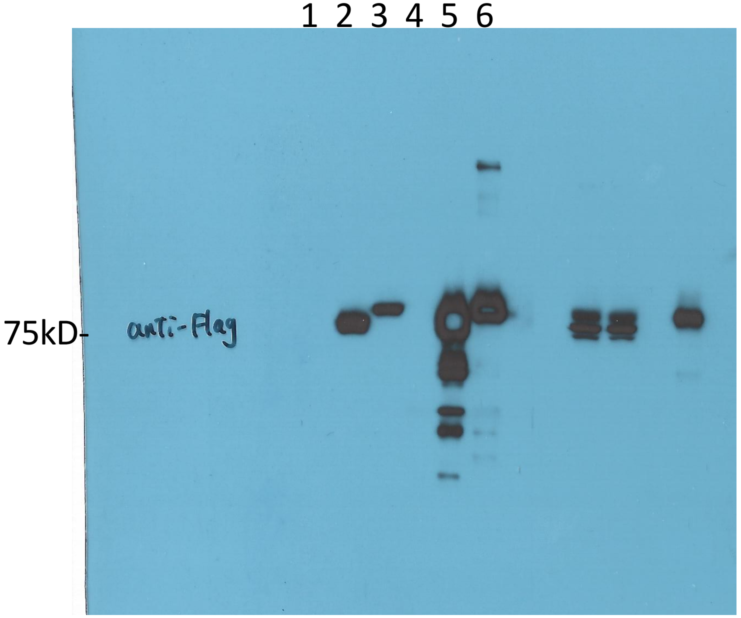

Anti-CNNM1

1 2 3 4 5 6

100kD-

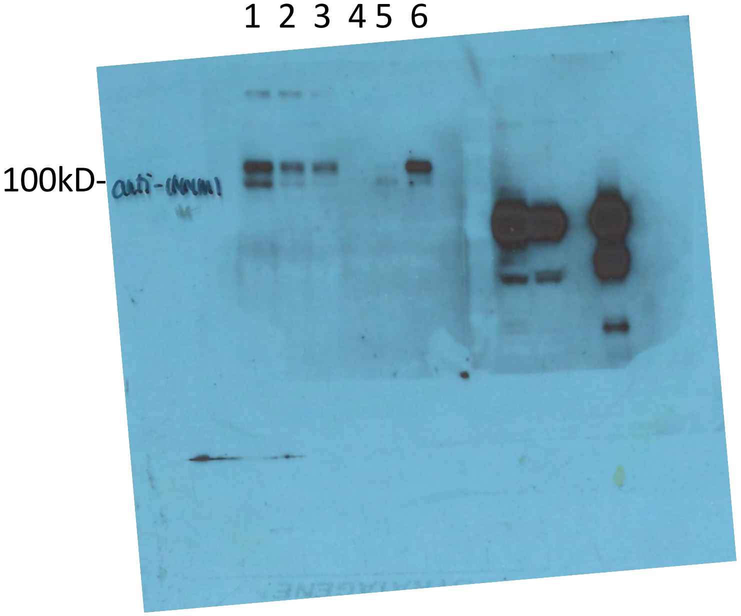

S16C Fig

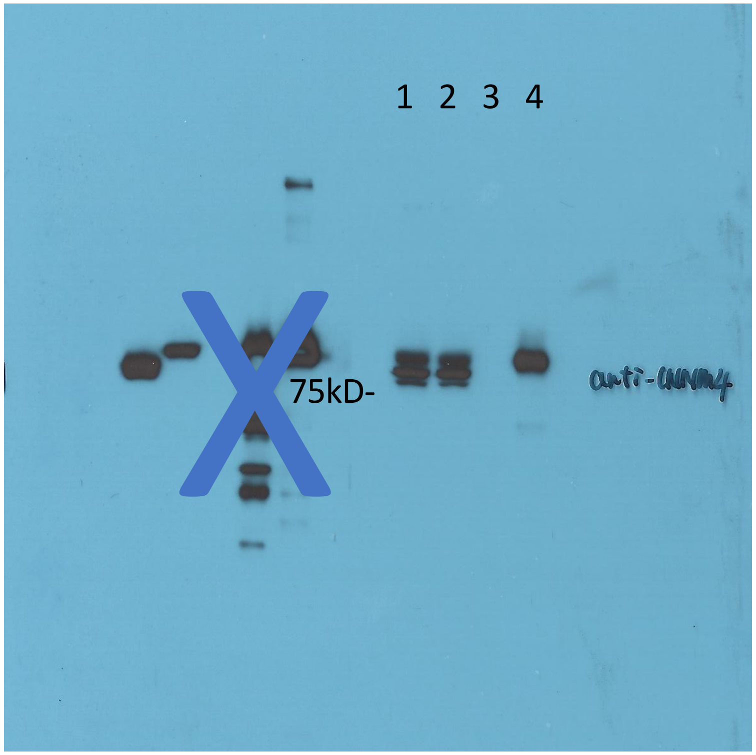

1. Lysate. No Transfection
2. Lysate. Flag-CNNM3
3. I.P. No Transfection
4. I.P. Flag-CNNM3

S16D Fig

1 2 3 4

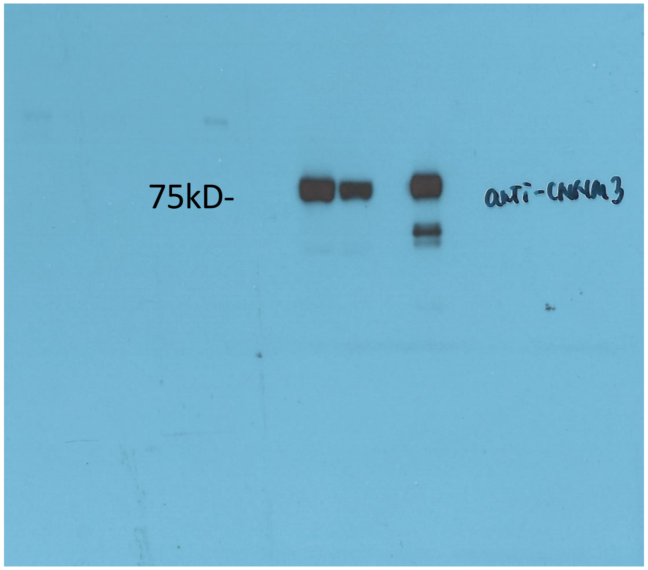

1. Lysate. No Transfection
2. Lysate. Flag-CNNM4
3. I.P. No Transfection
4. I.P. Flag-CNNM4

S16E Fig

1. 293T Lysate (Anti-myc)
2. 293T Lysate (anti-CNNM3)
3. 293T I.P. anti-myc
4. 293T I.P. anti-CNNM3
5. Lysis Buffer anti-myc
6. Lysis Buffer anti-CNNM3

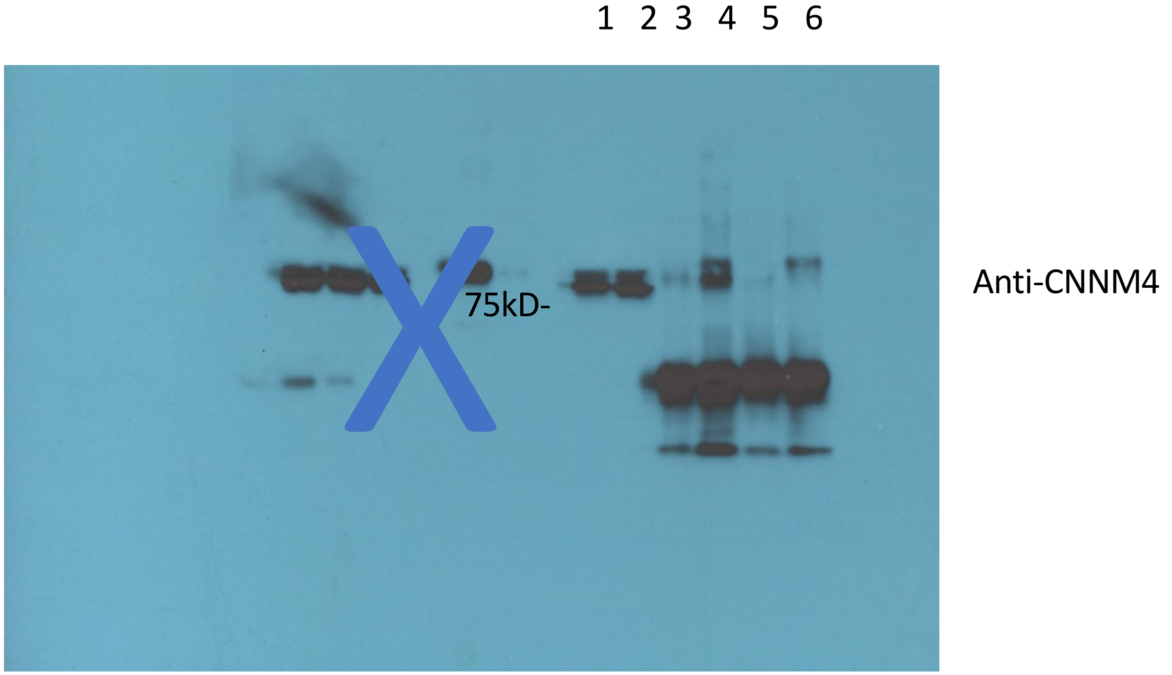

S16F Fig

1. 293T Lysate (Anti-myc)
2. 293T Lysate (anti-CNNM4)
3. 293T I.P. anti-myc
4. 293T I.P. anti-CNNM4
5. Lysis Buffer anti-myc
6. Lysis Buffer anti-CNNM4

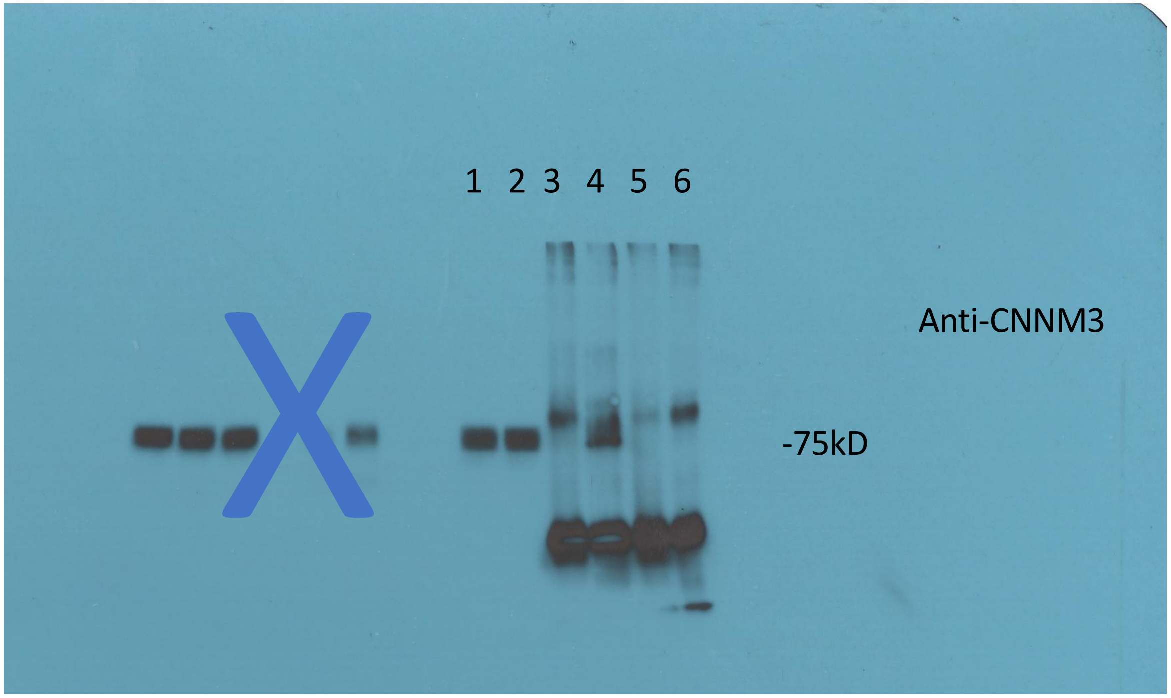

Supplement: S1 Raw Images — (PDF) [file pbio.3001496.s002.pdf]
