# Supplementary figures and images for: CNNM proteins selectively bind to the TRPM7 channel to stimulate divalent cation entry into cells
Source: PLoS Biol. 2021 Dec 20;19(12):e3001496. doi: 10.1371/journal.pbio.3001496 (PMC8726484; doi:10.1371/journal.pbio.3001496)

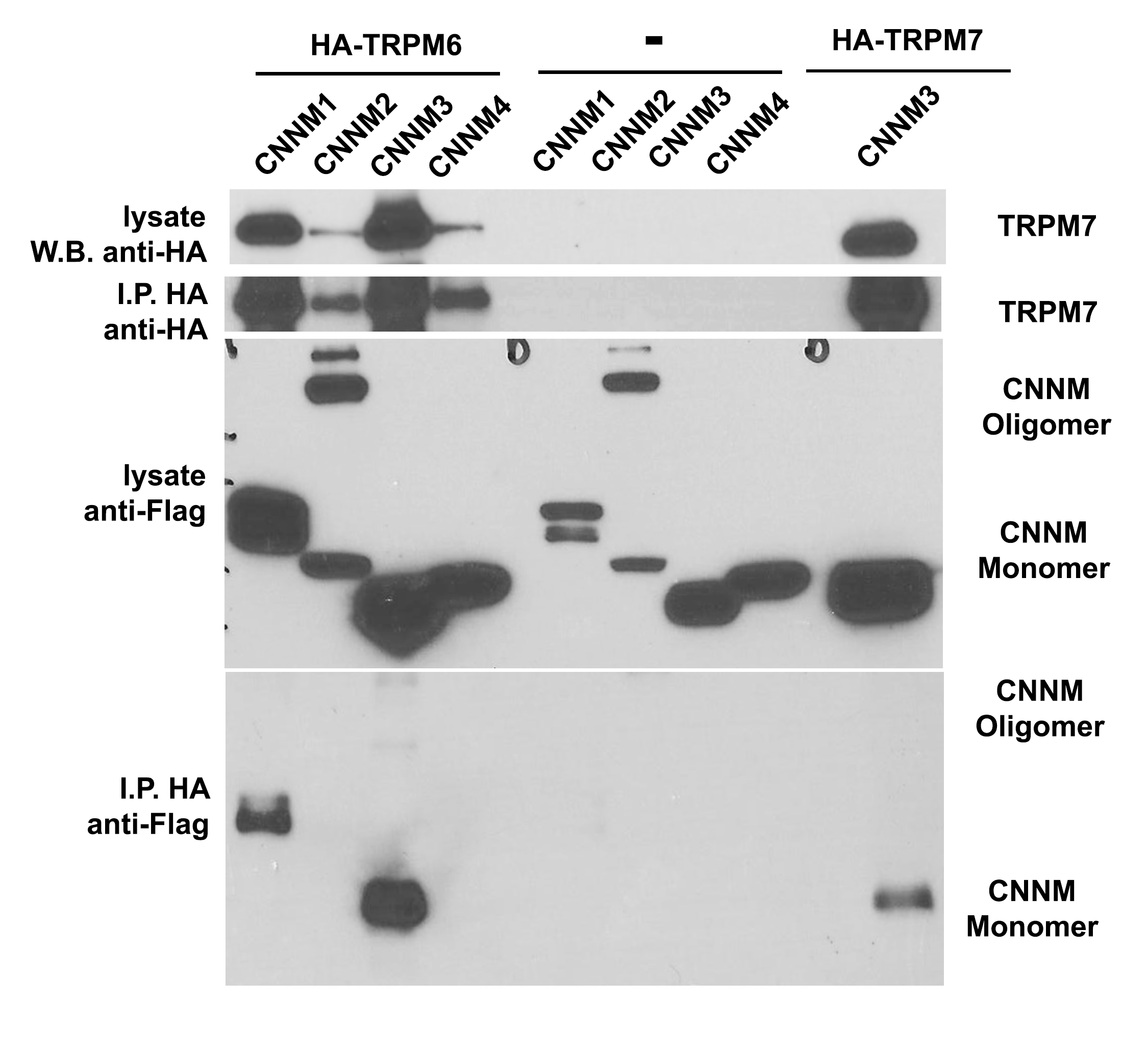

Supplement: S1 Fig — HA-TRPM6 was coexpressed with FLAG-tagged CNNM1-4 in HEK-293T cells, and the channel was immunoprecipitated with HA-agarose. CNNM1 and CNNM3 strongly interacted with TRPM6. An interaction between TRPM6 with CNNM2 and CNNM4 was not observed. CNNMs were not immunoprecipitated in HEK-293T cells N.T. with HA-TRPM6. Unprocessed images of blots are shown in S1 Raw Images. N.T., not transfected. (TIF) [file pbio.3001496.s004.tif]

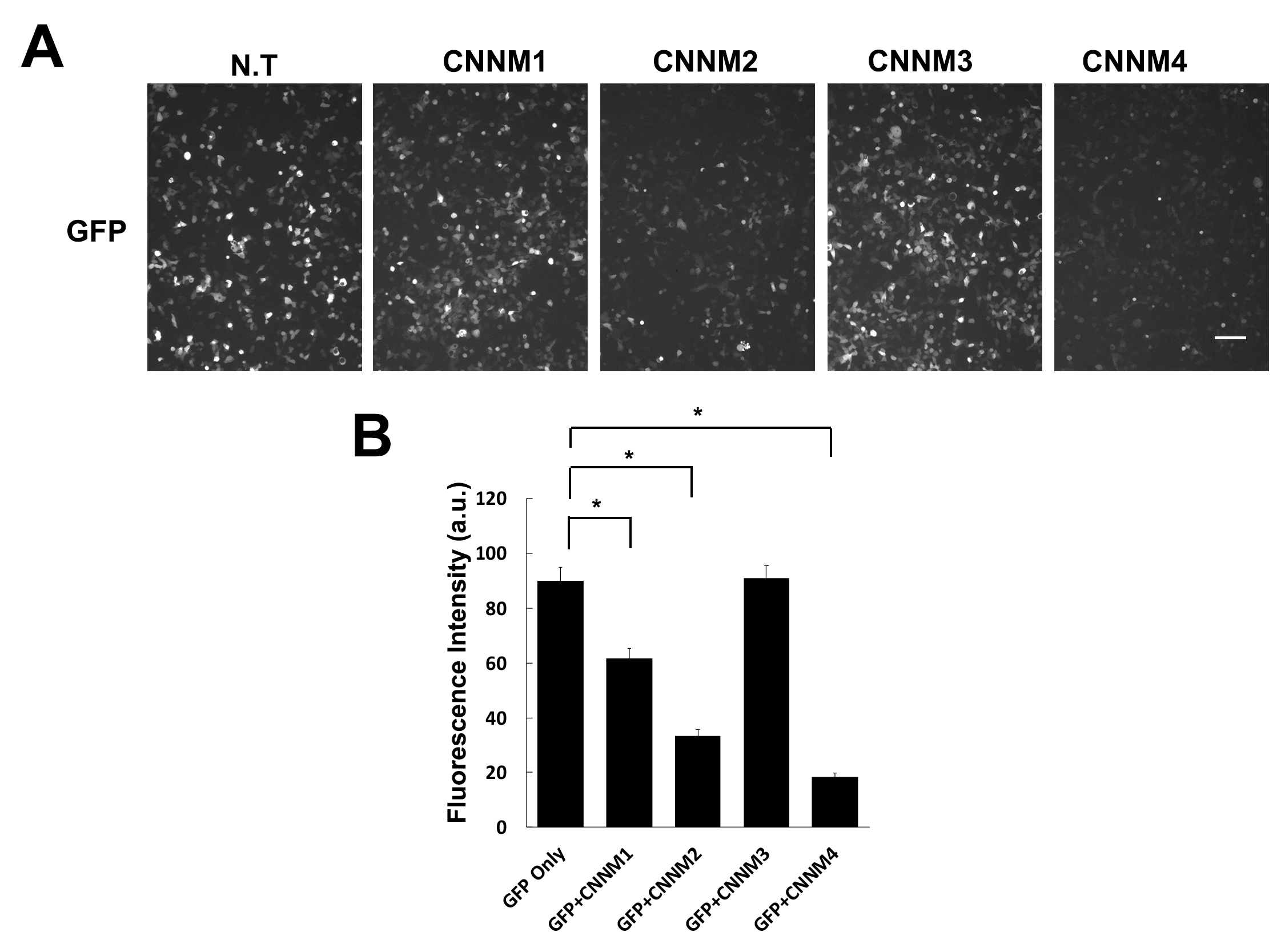

Supplement: S2 Fig — (A) EGFP was cotransfected with CNNM1-4 in HEK-293T cells and GFP protein expression was assessed by fluorescence microscopy. Coexpression of CNNM2 and CNNM4 with GFP significantly reduced GFP protein expression, most likely as a result of a decrease in intracellular Mg2+. (B) 100 cells were randomly selected for quantification. n = 100. * indicates a p-value of less than 0.05. The underlying data for this figure can be found in S1 Data. (TIF) [file pbio.3001496.s005.tif]

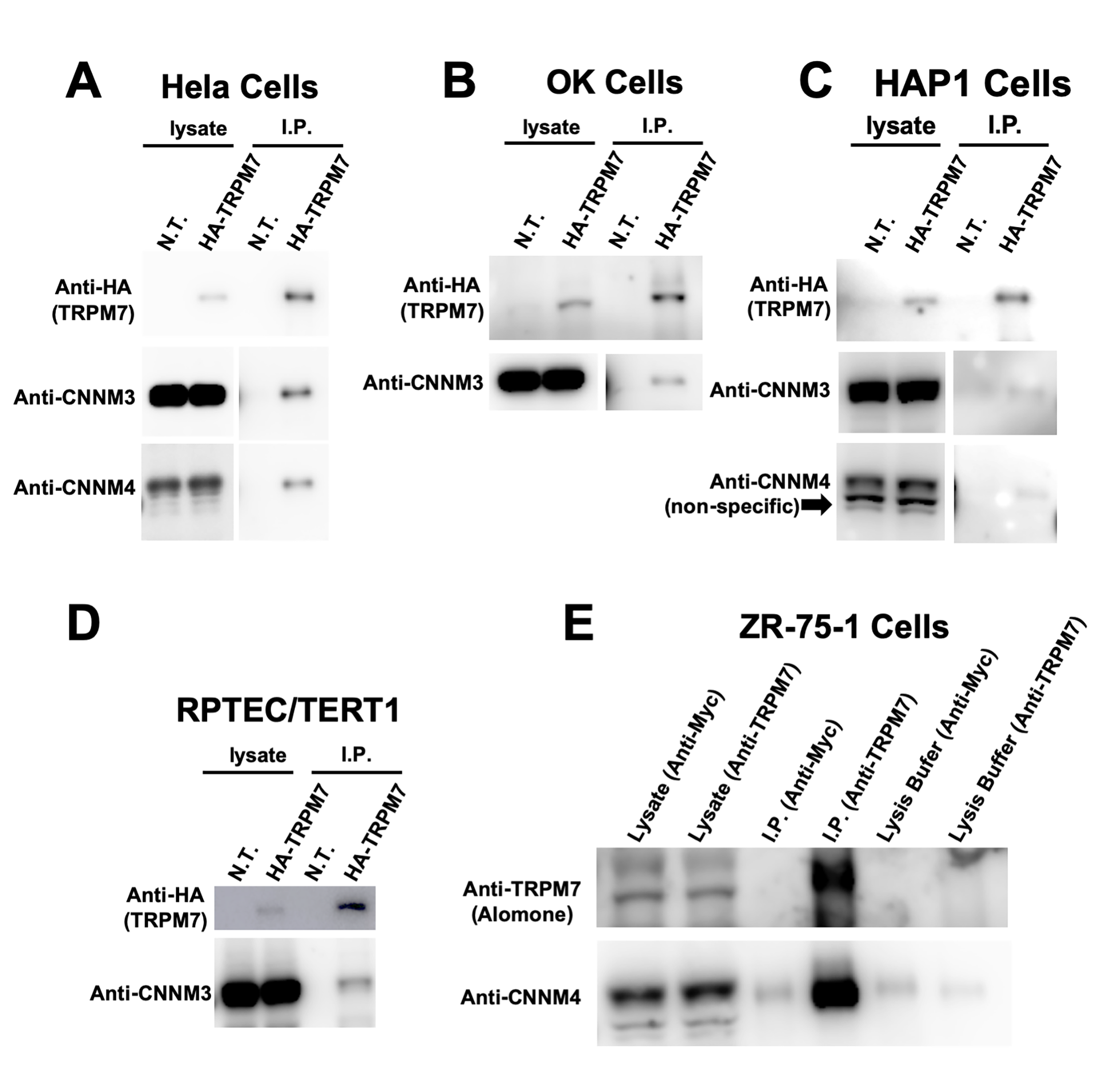

Supplement: S3 Fig — (A–D) HA-TRPM7 was overexpressed in the indicated cells and was the immunoprecipitated with HA-agarose. Native CNNM3 and CNNM4 coimmunoprecipitated with HA-TRPM7 transiently transfected into Hela cells. In OK cells transiently transfected with HA-TRPM7, only native CNNM3 was detected as interacting with HA-TRPM7. In HAP1 cells, both CNNM3 and CNNM4 could be weakly be coimmunoprecipitated with HA-TRPM7, which was expressed by viral transduction using the Ad-TRPM7-HA adenovirus. Note that the lower band in the lysate of the CNNM4 blot is a nonspecific band. In RPTEC/TERT cells transduced with Ad-TRPM7-HA, native CNNM3 coimmunoprecipitated with overexpressed HA-TRPM7. (E) Native TRPM7 is highly expressed in ZR-75-1 cells. Immunoprecipitation of native TRPM7 efficiently immunoprecipitated native CNNM4. Unprocessed images of blots are shown in S1 Raw Images. OK, opossum kidney proximal tubule. (TIF) [file pbio.3001496.s006.tif]

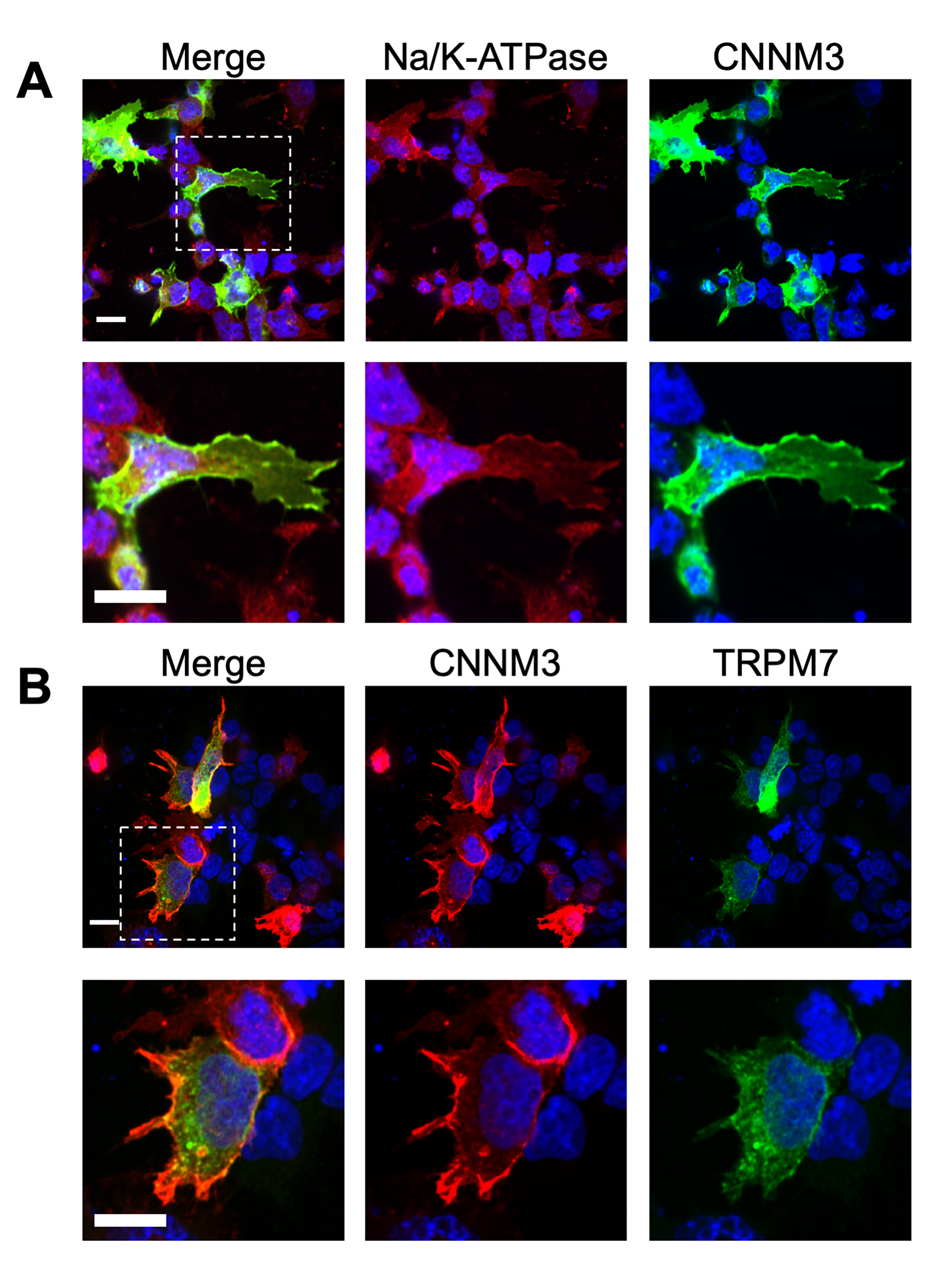

Supplement: S4 Fig — (A) Confocal images taken from HEK-293T cells transfected with FLAG-CNNM3. Shown are images of FLAG-CNNM3 and the endogenous Na/K-ATPase, a plasma membrane marker. CNNM3 is often found at the plasma membrane colocalized with the Na/K-ATPase. (B) Confocal images taken from HEK-293T cells transfected with HA-TRPM7 and FLAG-CNNM3.TRPM7 can be found colocalized with CNNM3 at the cell border but is also observed intracellularly. Dashed white boxes indicate regions of interest that were enlarged. Scale bar = 20 μM. (TIF) [file pbio.3001496.s007.tif]

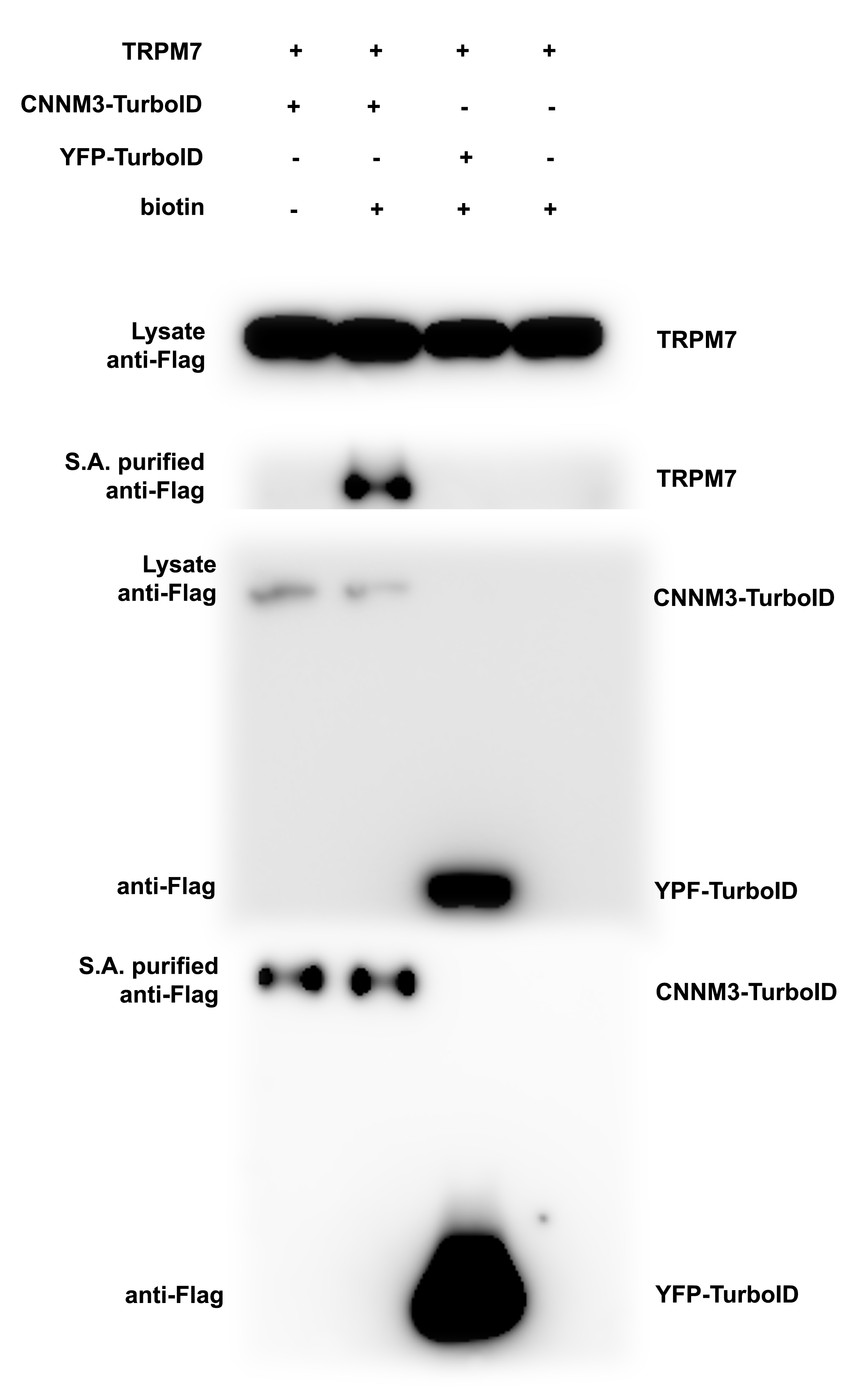

Supplement: S5 Fig — Proximity-dependent BioID is a recently developed method that allows the identification of proteins in the close vicinity (10 to 30 nm) of a protein of interest in living cells. We used the optimized E. coli BirA biotin ligase (TurboID) to create a fusion protein between CNNM3 and FLAG-tagged TurboID (CNNM3-FLAG-TurboID). As a negative control, we used EYFP fused to FLAG-tagged TurboID (YFP-FLAG-TurboID). CNNM3-TurboID and YFP-TurboID were transfected into 293-TRPM7 cells expressing FLAG-TRPM7. The top blot shows equal expression of TRPM7 in the lysate. In cells expressing TRPM7 with CNNM3-FLAG-TurboID treated with biotin, SA was able to efficiently purify biotinylated TRPM7. By contrast, for cells expressing the TRPM7 with the negative control YFP-TurboID, no biotinylated TRPM7 was purified with the SA. Blots show expression of CNNM3-TurboID and YFP-TurboID in cell lysates and following purification with SA. Unprocessed images of blots are shown in S1 Raw Images. BioID, biotin identification; SA, streptavidin agarose. (TIF) [file pbio.3001496.s008.tif]

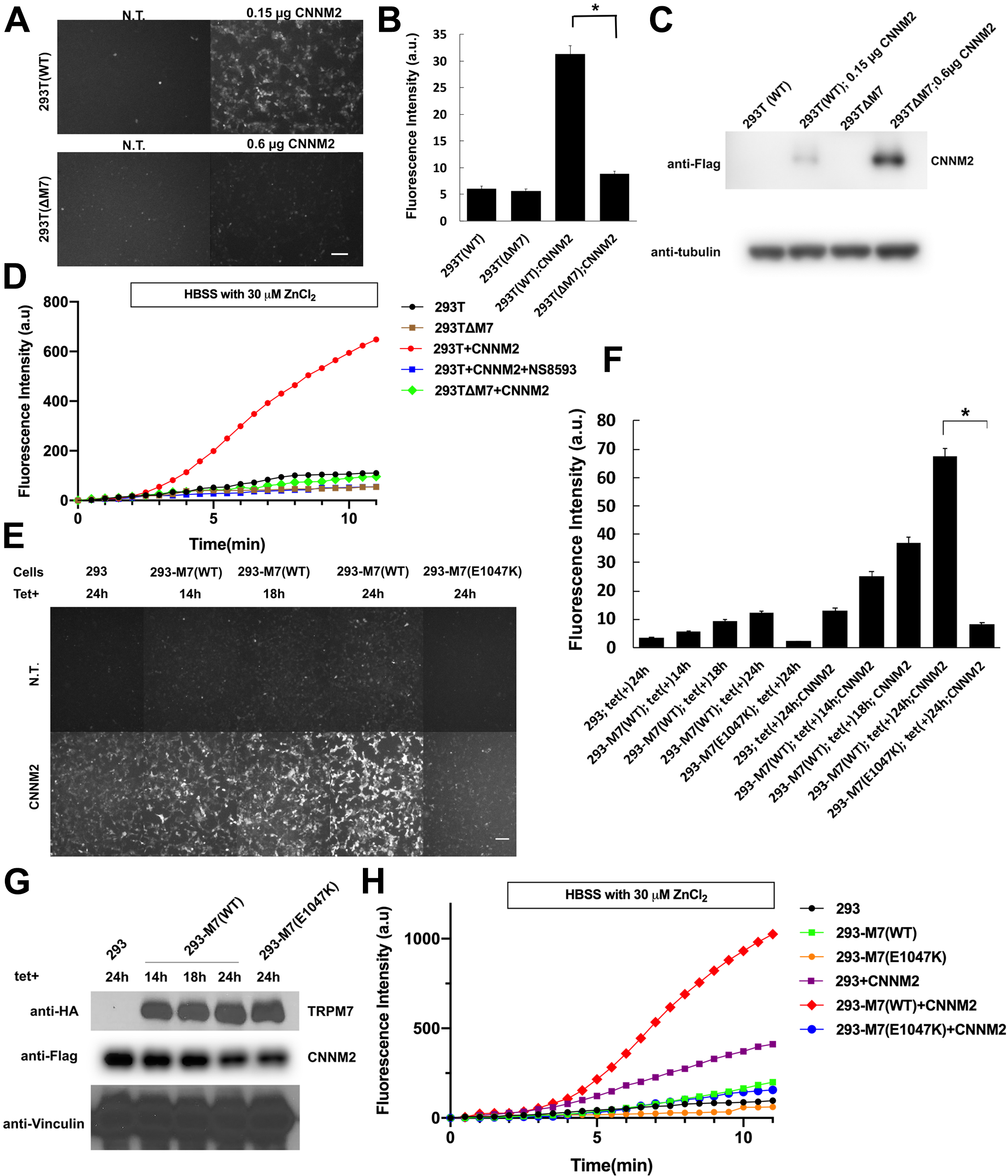

Supplement: S6 Fig — (A) Zinc influx assay using the Fluo-Zin-3 Zn2+ indicator was used to monitor the intracellular concentration of Zn2+ in intact cells. Overexpression of CNNM2 (0.15 μg CNNM2 DNA) in WT HEK-293T cells 293T(WT) elicited an increase cellular Zn2+. By contrast, overexpression of CNNM2 (using 0.6 μg instead of 0.15 μg CNNM2 DNA) in HEK-293T cells deficient in TRPM7 293T(ΔM7) did not increase cellular Zn2+. Shown are images taken at a time point 5 to 10 minutes after application of 30 μM ZnCl2. These data indicate that the native TRPM7 channel is required for CNNM-mediated divalent influx in HEK-293 cells. White scale bar = 100 μM. (B) Quantification of the results from (A). A total of 100 cells were randomly selected for quantification. n = 100. * indicates a p-value of less than 0.05. (C) Western blot showing expression of CNNM2 in the cells used for the experiment described in (A). (D) Separate time course measurements were also acquired to evaluate how CNNM2 overexpression affects the rate of Zn2+ influx compared in 293T(WT) versus 293T(ΔM7) cells. The TRPM7 channel inhibitor NS8593 (10 μM) was employed to further investigate whether the CNNM2-mediated increase in Fluo-Zin-3 fluorescence upon CNNM2 overexpression in 293T(WT) was dependent on TRPM7 channel function. HBSS media was replaced with HBSS containing 30 μM ZnCl2 for the period indicated. The fluorescence intensity of the cells (mean of 50 cells) were quantified for each time point. (E) Zinc influx assay using the Fluo-Zin-3 Zn2+ indicator was used to monitor the intracellular concentration of Zn2+ in intact cells. HA-tagged mouse TRPM7 (WT) and TRPM7-E1047K HEK-293 expressing cells 293-M7(E1047K) were used for these experiments (see Methods). Overexpression of CNNM2 with WT TRPM7, but not the TRPM7-E1047K pore-inactive mutant increased Zn2+ uptake. However, no Zn2+ uptake was observed when a channel-inactive mutant was coexpressed with CNNM2. White scale bar = 100 μM. (F) Quantification of the results from (E [file pbio.3001496.s009.tif]

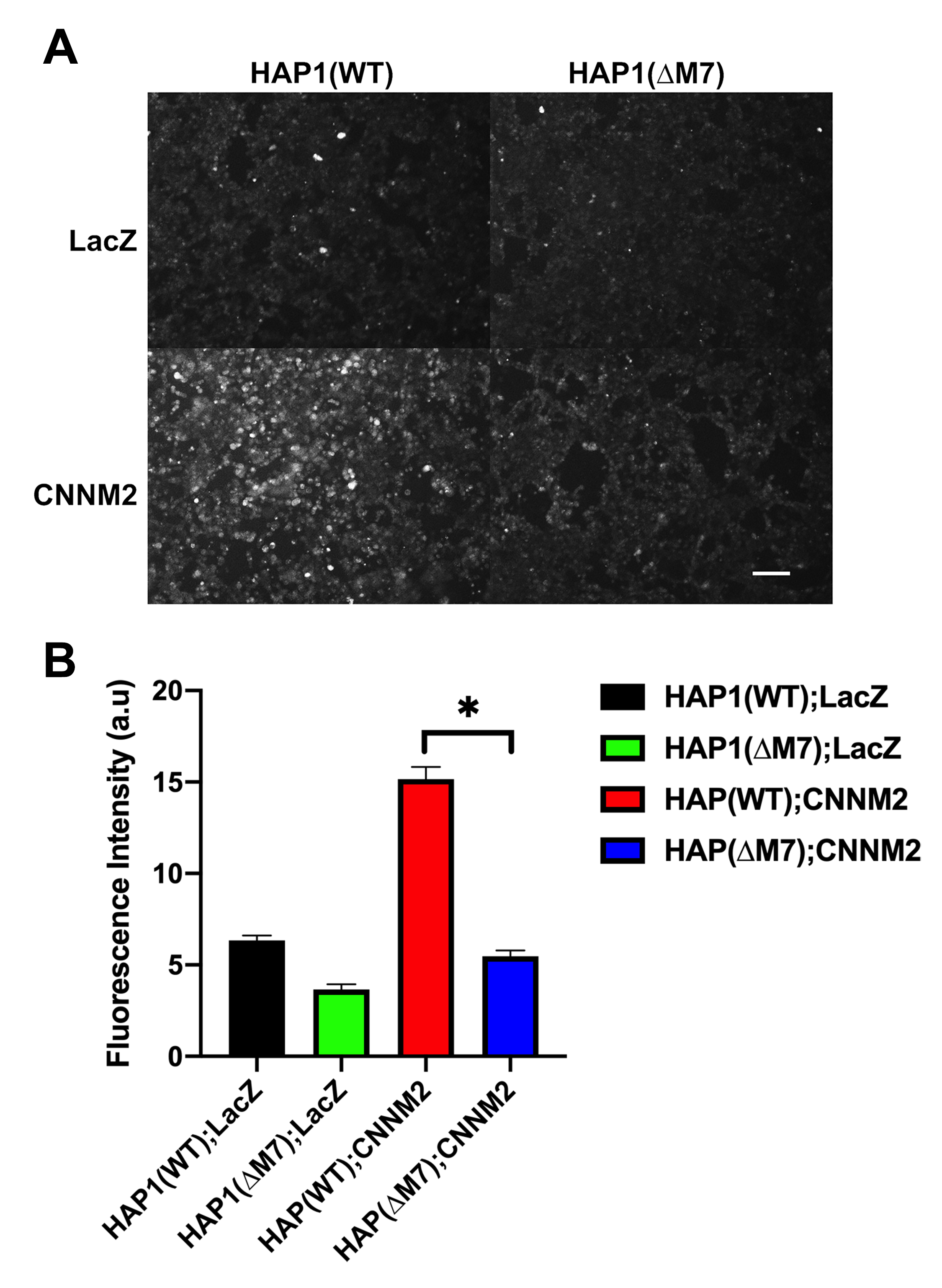

Supplement: S7 Fig — (A) A Zinc influx assay using the Fluo-Zin-3 Zn2+ indicator was used to monitor the intracellular concentration of Zn2+ in intact cells. Shown are images taken at a time point 5 to 10 minutes after application of 30 μM ZnCl2. Overexpression of CNNM2 in WT HAP1 cells HAP1(WT) using a recombinant adenovirus expressing FLAG-CNNM2 elicited an increase in cellular Zn2+. By contrast, overexpression of CNNM2 in HAP1 cells deficient in TRPM7 HAP1(ΔM7) did not significantly increase cellular Zn2+. Expression of LacZ was used as a negative control. These data indicate that the native TRPM7 channel is required for CNNM-mediated divalent influx in HAP1 cells. White scale bar = 100 μM. (B) Quantification of the data in (A). A total of 100 cells were randomly selected for quantification. n = 100. * indicates a p-value of less than 0.05. The underlying data for this figure can be found in S1 Data. WT, wild-type. (TIF) [file pbio.3001496.s010.tif]

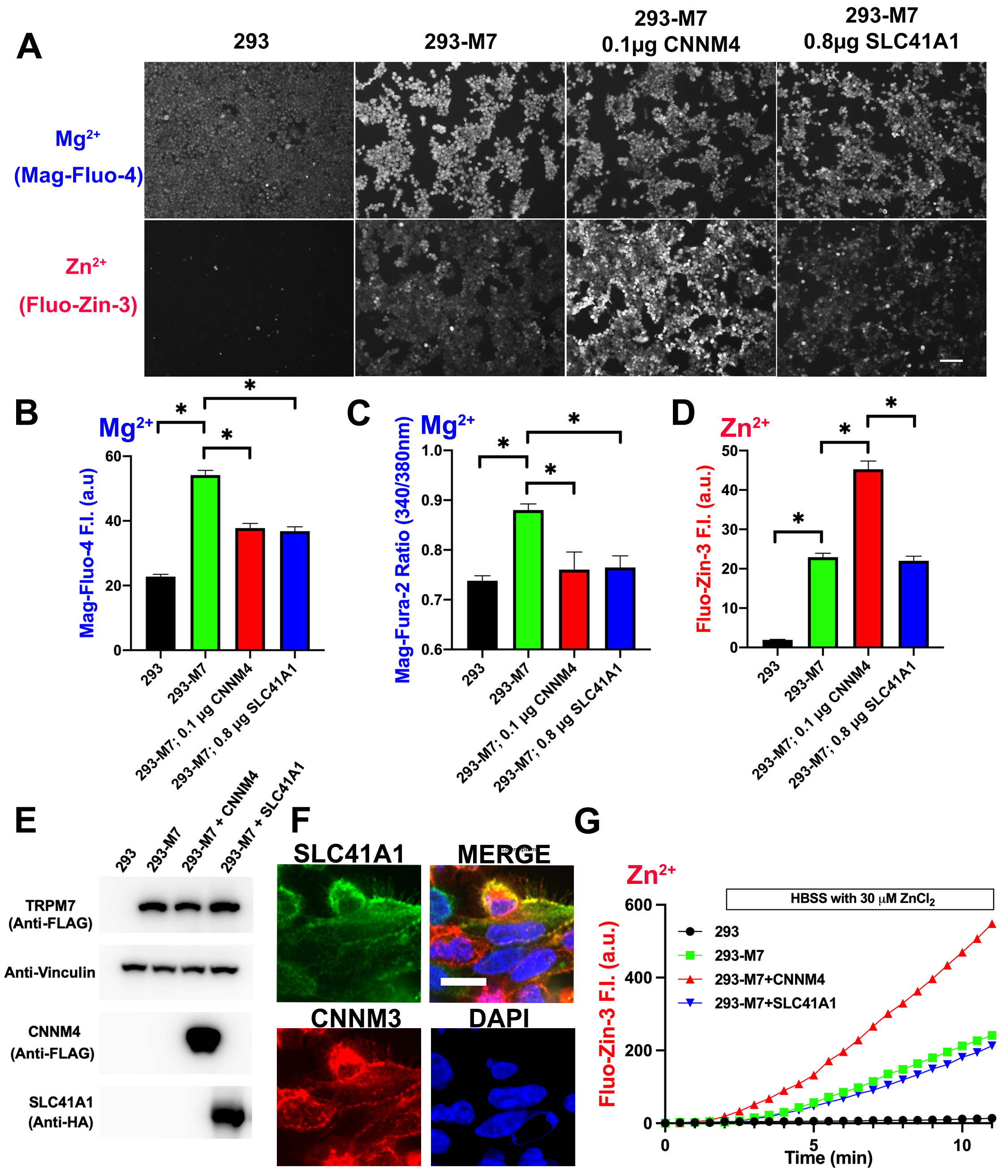

Supplement: S8 Fig — (A) A Zinc influx assay using the Fluo-Zin-3 Zn2+ indicator was used to monitor the intracellular concentration of Zn2+ in intact cells. Shown are images taken at a time point between 5 and 10 minutes after application of 30 μM ZnCl2. In parallel experiments, intracellular Mg2+ levels were assessed using the Mag-Fluo-4 Mg2+ indicator. White scale bar = 100 μM. (B) Quantification of Mag-Fluo-4 results from (A). A total of 100 cells were randomly selected for quantification. n = 100.* indicates a p-value of less than 0.05. (C) Separate control experiments were performed using the Mag-Fura-2 dye to show that the magnesium levels of 293-TRPM7 cells expressing CNNM4 as well as SLC41A1 decreased compared to 293-TRPM7 cells alone. Plotted is the ratio of the fluorescence intensity at 510 nm from 340 nm versus 380 nm excitation. 30 cells were randomly selected for quantification. n = 30. * indicates a p-value of less than 0.05. (D) Quantification of Fluo-Zin-3 Zn2+ indicator fluorescence intensity from (A). A total of 100 cells were randomly selected for quantification. n = 100.* indicates a p-value of less than 0.05. (E) Western blot evaluating expression of CNNM4, SLC41A1, and TRPM7 in the indicated cell lines. (F) Confocal images taken from HEK-293T cells transfected with MYC-SLC41A1 and FLAG-CNNM3. SLC41A1 can be found colocalized with CNNM3 at the cell border but is also observed intracellularly, which may be due to the effects of its overexpression. Scale bar = 20 μM. (G) Separate time course measurements were also acquired to demonstrate rate of Zn2+ influx in the different cell lines. HBSS media was replaced with HBSS containing 30 μM ZnCl2 for the period indicated. The fluorescence intensity of the cells (mean of 50 cells) were quantified for each time point. Unprocessed images of blots are shown in S1 Raw Images. The underlying data for this figure can be found in S1 Data. HBSS, Hanks’ balanced salt solution. (TIF) [file pbio.3001496.s011.tif]

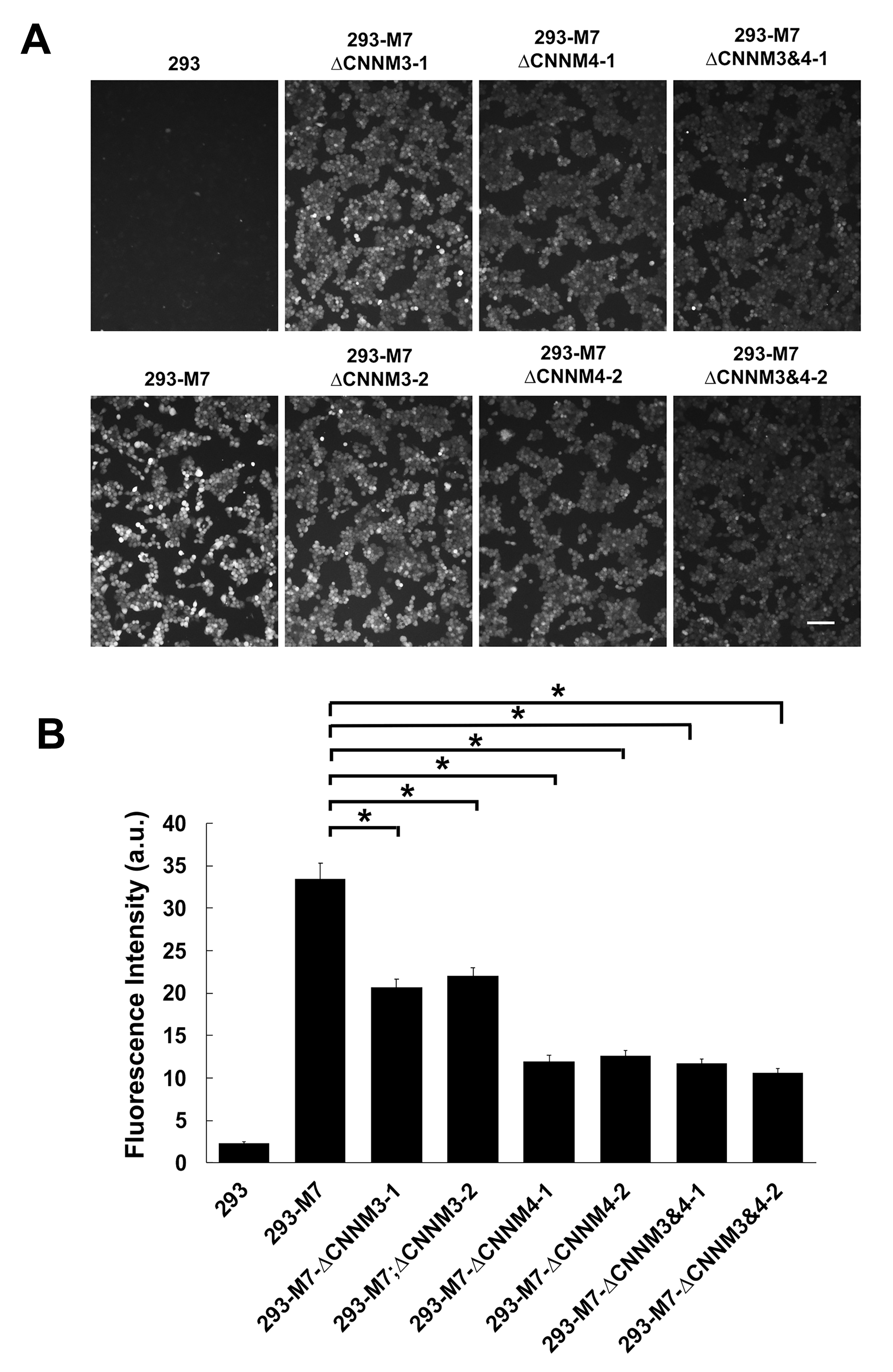

Supplement: S9 Fig — (A) Zinc influx assay using the Fluo-Zin-3 Zn2+ indicator was used to monitor TRPM7 function in independent cell lines in which CNNM3, CNNM4, and both CNNM3 and CNNM4 were deleted by CRISPR/Cas-9 from 293-TRPM7 cells (293-M7-ΔCNNM3, 293-M7-ΔCNNM4, 293-M7-ΔCNNM3/4. Shown are images taken at a time point between 5 and 10 minutes after application of 30 μM ZnCl2. Similar loss of TRPM7 function was observed among the 2 independent lines tested, with deletion of CNNM3 and CNNM4 producing the largest loss-of-function. All the cells in the assay were treated with tetracycline to induce TRPM7 expression. White scale bar = 100 μM. (B) Quantification of the results from (A). A total of 100 cells were randomly selected for quantification. n = 100. * indicates a p-value of less than 0.05. The underlying data for this figure can be found in S1 Data. KO, knockout. (TIF) [file pbio.3001496.s012.tif]

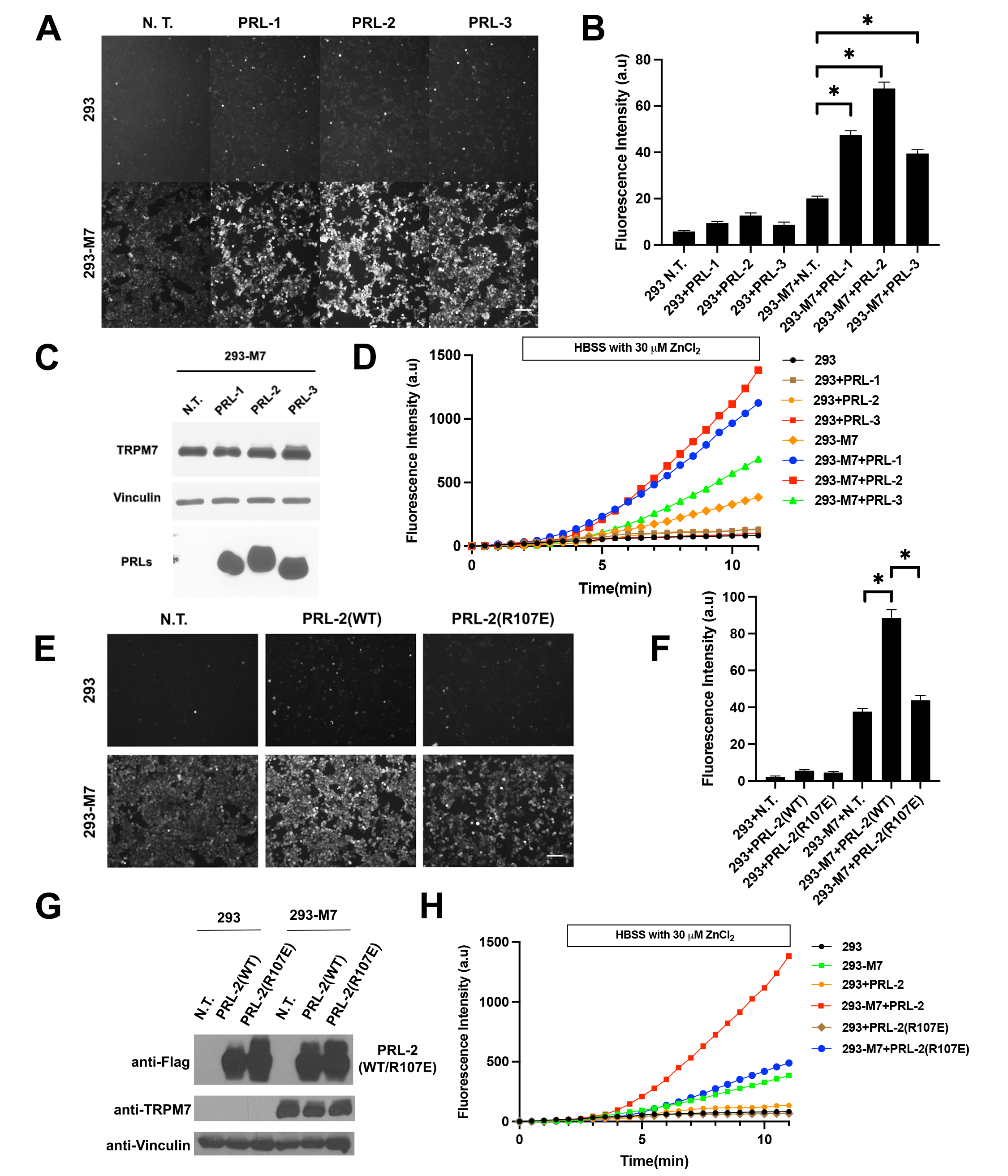

Supplement: S10 Fig — (A) Zinc influx assay using the Fluo-Zin-3 Zn2+ indicator was used to monitor the intracellular concentration of Zn2+ in intact cells. Shown are images taken at a time point between 5 and 10 minutes after application of 30 μM ZnCl2. Coexpression of the PRL isoforms PRL-1, PRL2, and PRL-3 all stimulated TRPM7-dependent Zn2+ uptake. White scale bar = 100 μM. (B) Quantification of the data from (A). A total of 100 cells were randomly selected for quantification. n = 100. * indicates a p-value of less than 0.05. (C) Western blot demonstrating expression levels of TRPM7 and PRL isoforms in the experimental samples from (A). (D) Separate time course measurements were also acquired to demonstrate rate of Zn2+ influx in the different cell lines. HBSS media was replaced with HBSS containing 30 μM ZnCl2 for the period indicated. The fluorescence intensity of the cells (mean of 50 cells) were quantified for each time point. (E) Coexpression of WT PRL-2 (PRL-2(WT)) but not the CNNM-binding deficient mutant PRL-2(R017E) stimulates TRPM7-dependent Zn2+ uptake. White scale bar = 100 μM. (F) Quantification of the data from (E). A total of 100 cells were randomly selected for quantification. n = 100. * indicates a p-value of less than 0.05. (G) Western blot demonstrating expression levels of TRPM7, PRL-2(WT), PRL-2(R107E) in the experimental samples from (E). (H) Separate time course measurements were also acquired to demonstrate rate of Zn2+ influx in the different cell lines. HBSS media was replaced with HBSS containing 30 μM ZnCl2 for the period indicated. The fluorescence intensity of the cells (mean of 50 cells) were quantified for each time point. Unprocessed images of blots are shown in S1 Raw Images. The underlying data for this figure can be found in S1 Data. HBSS, Hanks’ balanced salt solution; PRL, phosphatase of regenerating liver; WT, wild-type. (TIF) [file pbio.3001496.s013.tif]

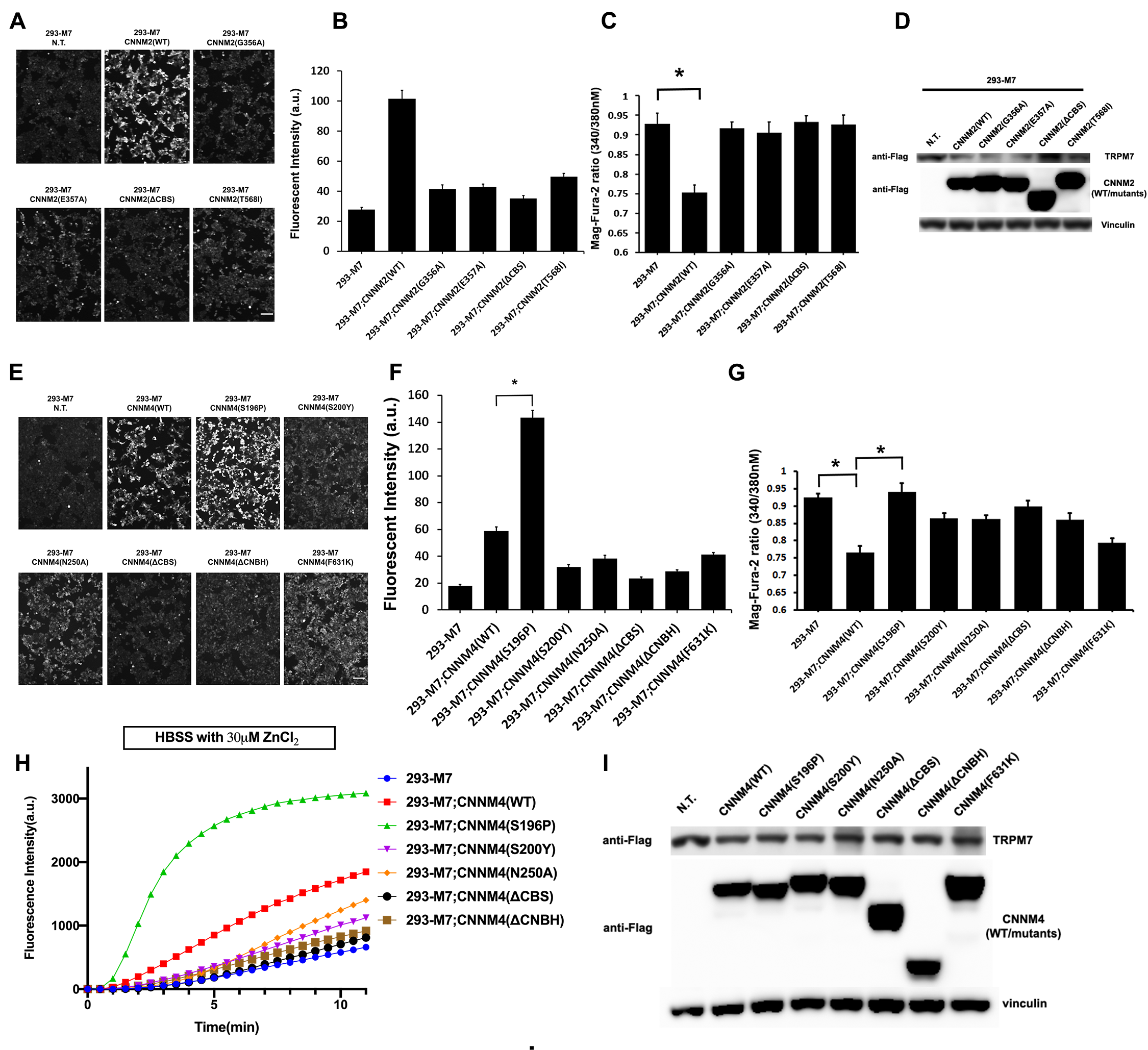

Supplement: S11 Fig — (A) A Zinc influx assay using the Fluo-Zin-3 Zn2+ indicator was used to monitor the intracellular concentration of Zn2+ in intact cells. Shown are images taken at a time point between 5 and 10 minutes after application of 30 μM ZnCl2. Coexpression of mutational variants of CNNM2 known to disrupt CNNM2 Mg2+ efflux activity also affected TRPM7-mediated increases in intracellular free Zn2+ to varying degrees. (B) Quantification of the data from (A). A total of 50 cells were randomly selected for quantification. n = 50. * indicates a p-value of less than 0.05. (C) Separate control experiments were performed using the Mag-Fura-2 dye to show that the magnesium levels of 293-TRPM7 cells expressing CNNM2 are decreased compared to 293-TRPM7 cells alone and that CNNM2 mutants used in (A) do not effectively lower intracellular Mg2+ levels as effectively as WT CNNM2. Plotted is the ratio of the fluorescence intensity at 510 nm from 340 nm versus 380 nm excitation. A total of 50 cells were randomly selected for quantification. n = 50. * indicates a p-value of less than 0.05. (D) Western blot validating expression of TRPM7 and CNNM2 WT and mutants. Vinculin is shown as a loading control. (E) A Zinc influx assay using the Fluo-Zin-3 Zn2+ indicator was used to monitor the intracellular concentration of Zn2+ in intact cells. Shown are images taken at a time point between 5 and 10 minutes after application of 30 μM ZnCl2. Coexpression of mutational variants of CNNM4 known to disrupt CNNM4 Mg2+ efflux activity also affected TRPM7-mediated increases in intracellular free Zn2+ to varying degrees. Strikingly, the CNNM4(S196P) mutant, which is reported to disrupted Mg2+ binding to the CNNM4 transmembrane domain, enhanced Zn2+ influx compared to WT CNNM4 and the other tested variants. (F) Quantification of the data from (E). A total of 50 cells were randomly selected for quantification. n = 50. * indicates a p-value of less than 0.05. (G) Separate control experiments were performed using t [file pbio.3001496.s014.tif]

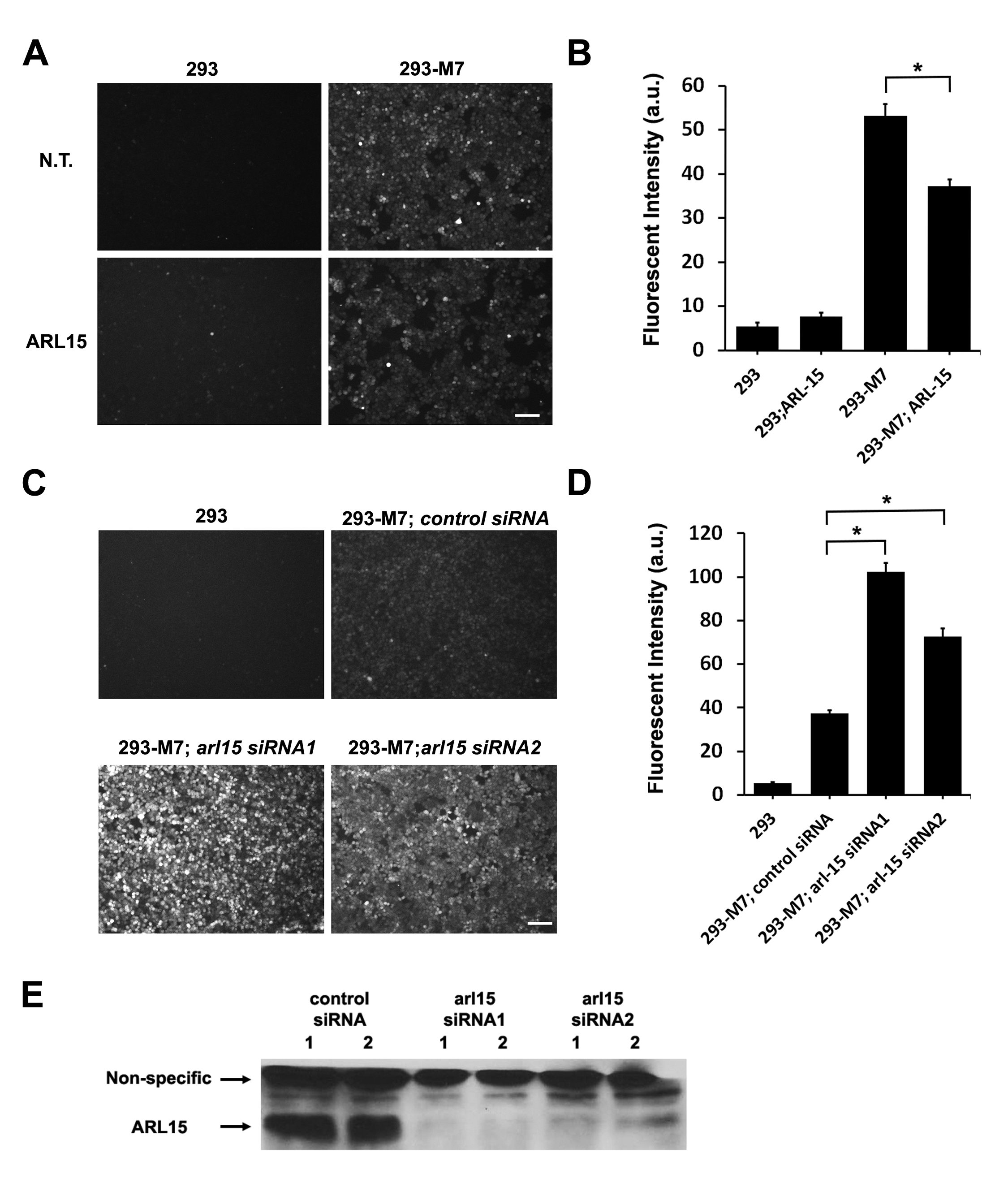

Supplement: S12 Fig — (A) Zinc influx assay using the Fluo-Zin-3 Zn2+ indicator was used to monitor the intracellular concentration of Zn2+ in intact cells. Shown are images taken at a time point between 5 and 10 minutes after application of 30 μM ZnCl2. Coexpression of the ARL15 protein suppressed TRPM7-dependent Zn2+ uptake compared to nontransfected cells. White scale bar = 100 μM. (B) Quantification of the data from (A). A total of 50 cells were randomly selected for quantification. n = 50. * indicates a p-value of less than 0.05. (C) Zinc influx assay using Fluo-Zin-3 Zn2+ indicator was used to monitor the intracellular concentration of Zn2+ in intact cells. Shown are images taken at a time point between 5 and 10 minutes after application of 30 μM ZnCl2. Coexpression of siRNAs targeting the ARL15 protein increased TRPM7-dependent Zn2+ uptake compared to a control siRNA. White scale bar = 100 μM. (D) Quantification of the data from (A). A total of 50 cells were randomly selected for quantification. n = 50. * indicates a p-value of less than 0.05. (E) Western blot validating ability of 2 independent siRNAs targeting ARL15 to knockdown expression of ARL15 compared to the nonsilencing control. Unprocessed images of blots are shown in S1 Raw Images. The underlying data for this figure can be found in S1 Data. (TIF) [file pbio.3001496.s015.tif]

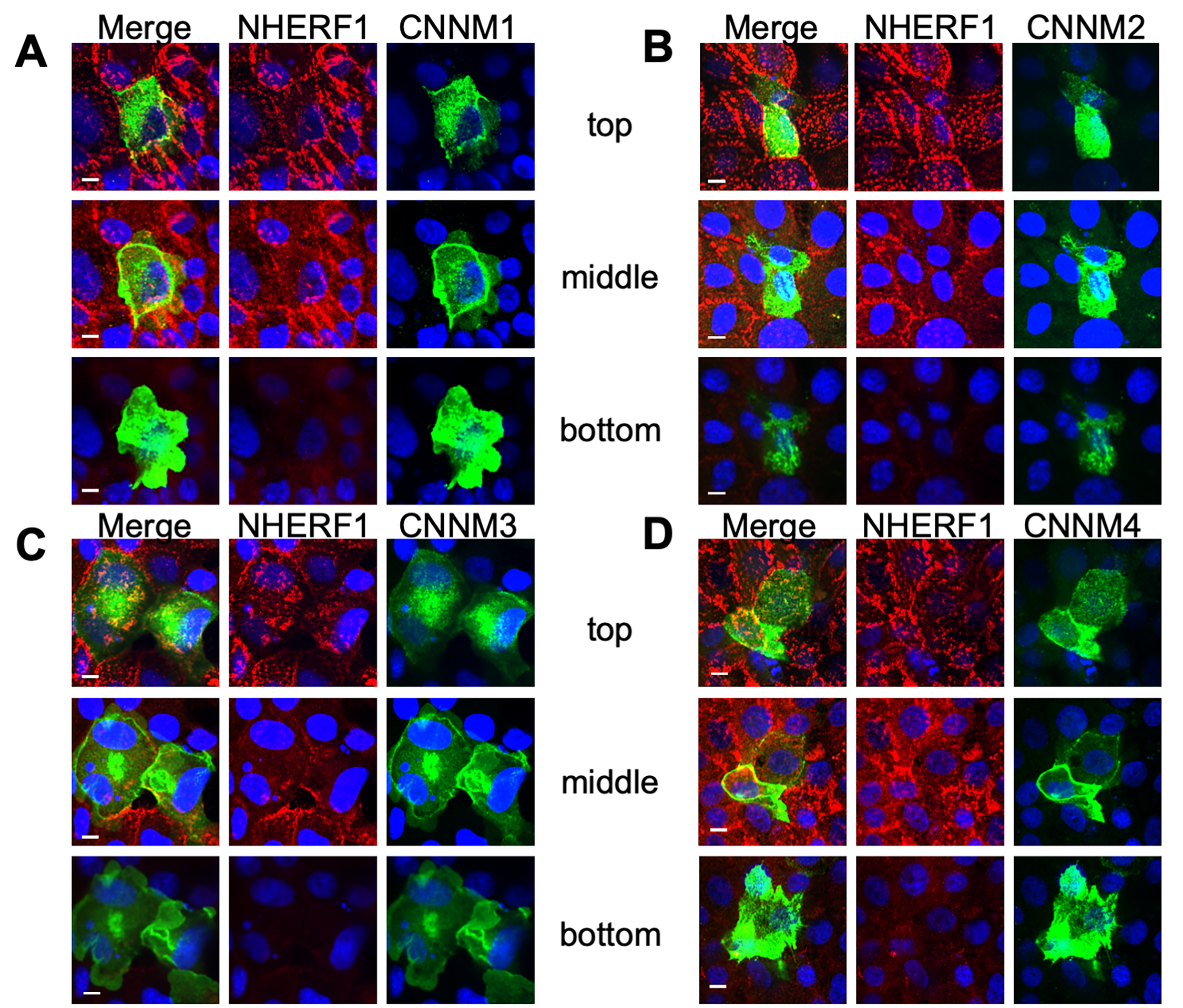

Supplement: S13 Fig — Shown are confocal microscopy images taken of the top (apical), middle, and bottom of cells. FLAG-tagged CNNM proteins were stained with the rabbit monoclonal anti-FLAG antibody. NHERF-1, an apical membrane marker that localizes to microvilli on polarized epithelial cells, was stained using a mouse monoclonal antibody. FLAG-CNNM isoforms were transiently transfected in OK cells and stained following a standard protocol (see Methods). Shown are the localization for (A) CNNM1, (B) CNNM2, (C) CNNM3, and (D) CNNM4. Scale bar = 20 μM. OK, opossum kidney proximal tubule. (TIF) [file pbio.3001496.s016.tif]

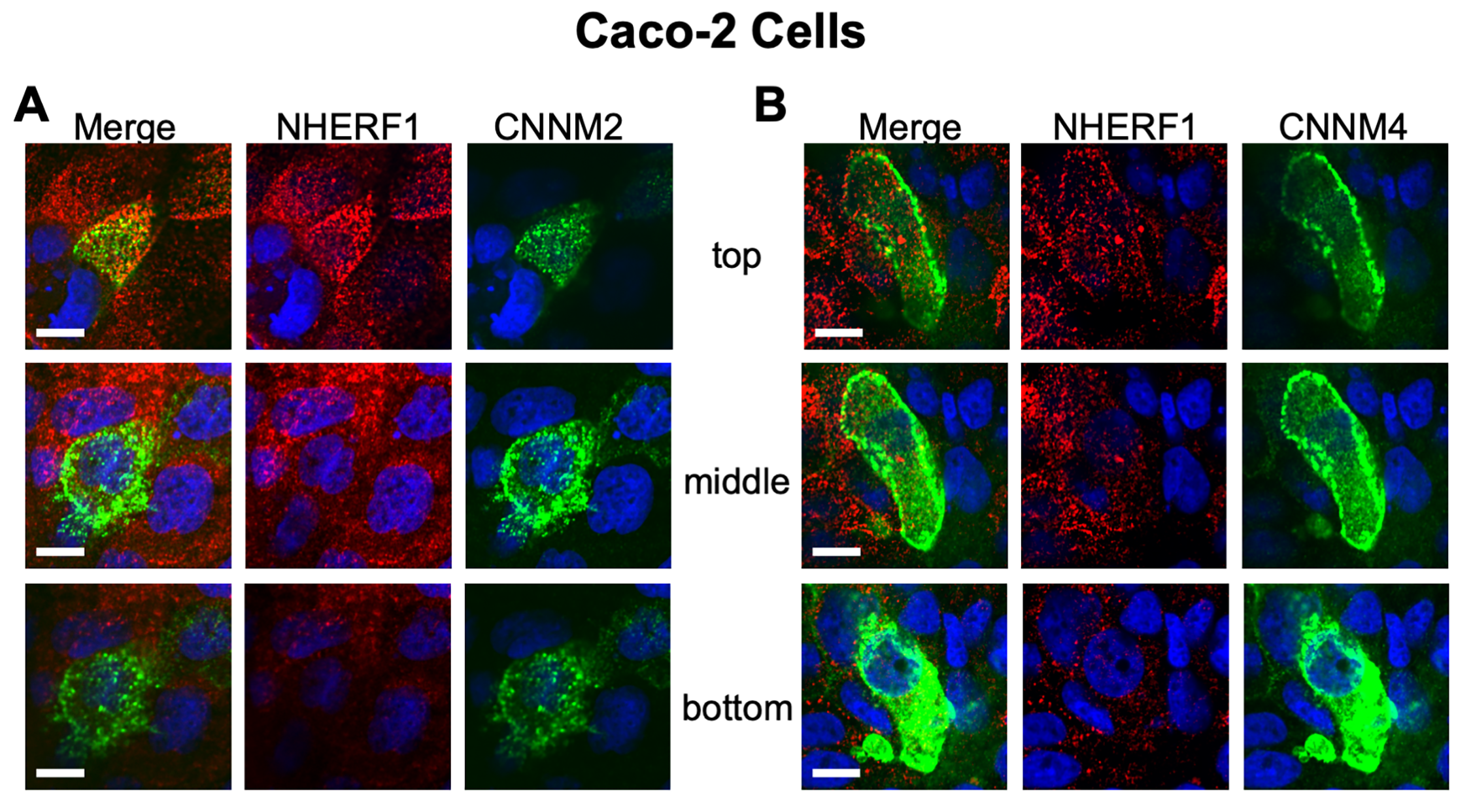

Supplement: S14 Fig — Shown are confocal microscopy images taken of the top (apical), middle, and bottom of cells. FLAG-CNNM2 and FLAG-CNNM4 were stained with the rabbit monoclonal anti-FLAG antibody. NHERF-1, an apical membrane marker that localizes to microvilli on polarized epithelial cells, was stained with a mouse monoclonal antibody. FLAG-CNNM2 and FLAG-CNNM4 isoforms were virally transduced into Caco-2 cells and stained following a standard protocol (see Methods). Shown are the localization for (A) CNNM2 and (B) CNNM4. Scale bar = 20 μM. (TIF) [file pbio.3001496.s017.tif]

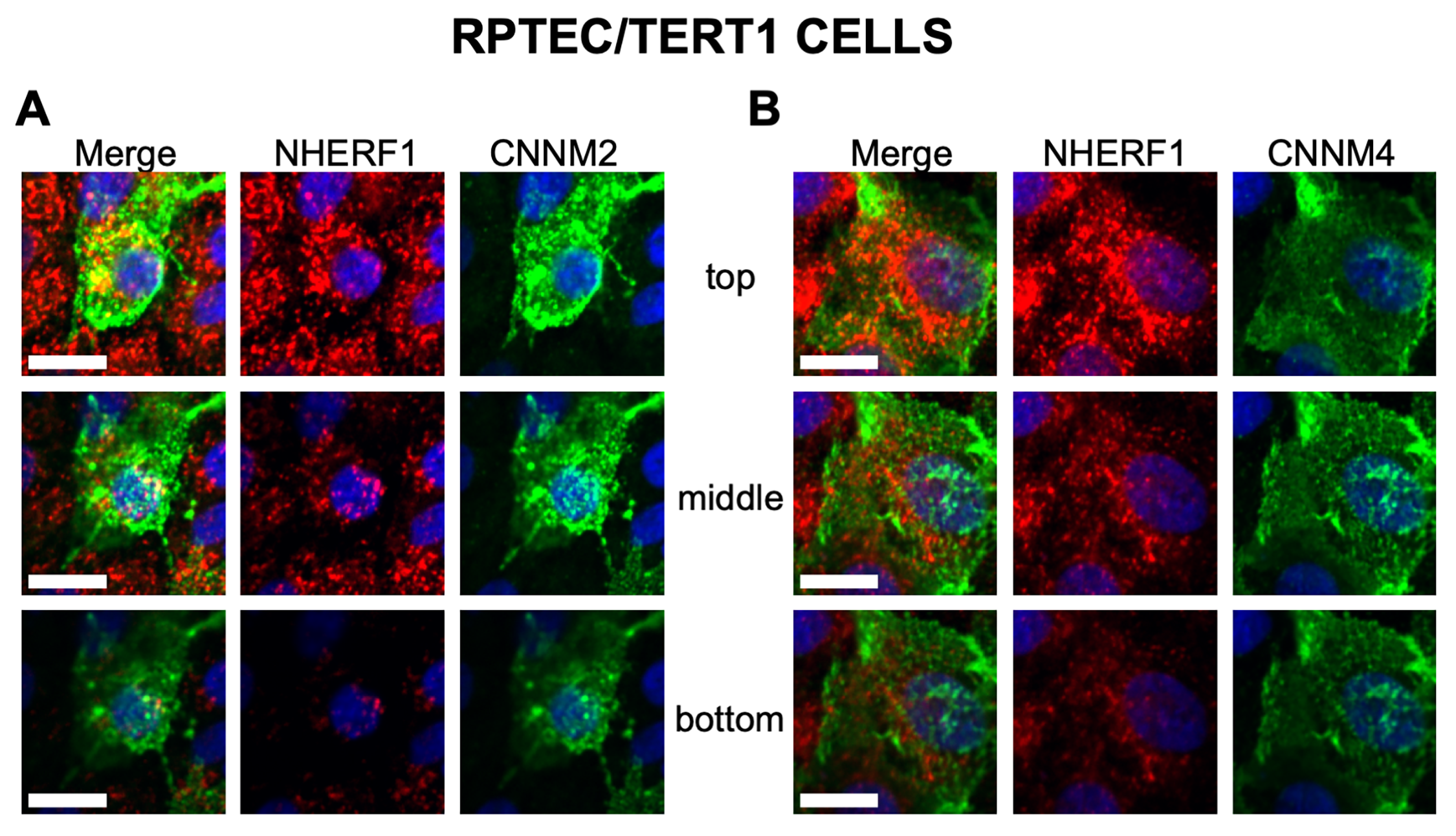

Supplement: S15 Fig — Shown are confocal microscopy images taken of the top (apical), middle, and bottom of cells. FLAG-CNNM2 and FLAG-CNNM4 were stained with the rabbit monoclonal anti-FLAG antibody. NHERF-1, an apical membrane marker that localizes to microvilli on polarized epithelial cells, was stained with a mouse monoclonal antibody. FLAG-CNNM2 and FLAG-CNNM4 isoforms were virally transduced into RPTEC/TERT1 cells and stained following a standard immunofluorescence protocol (see Methods). Shown are the localization for (A) CNNM2 and (B) CNNM4. Scale bar = 20 μM. (TIF) [file pbio.3001496.s018.tif]

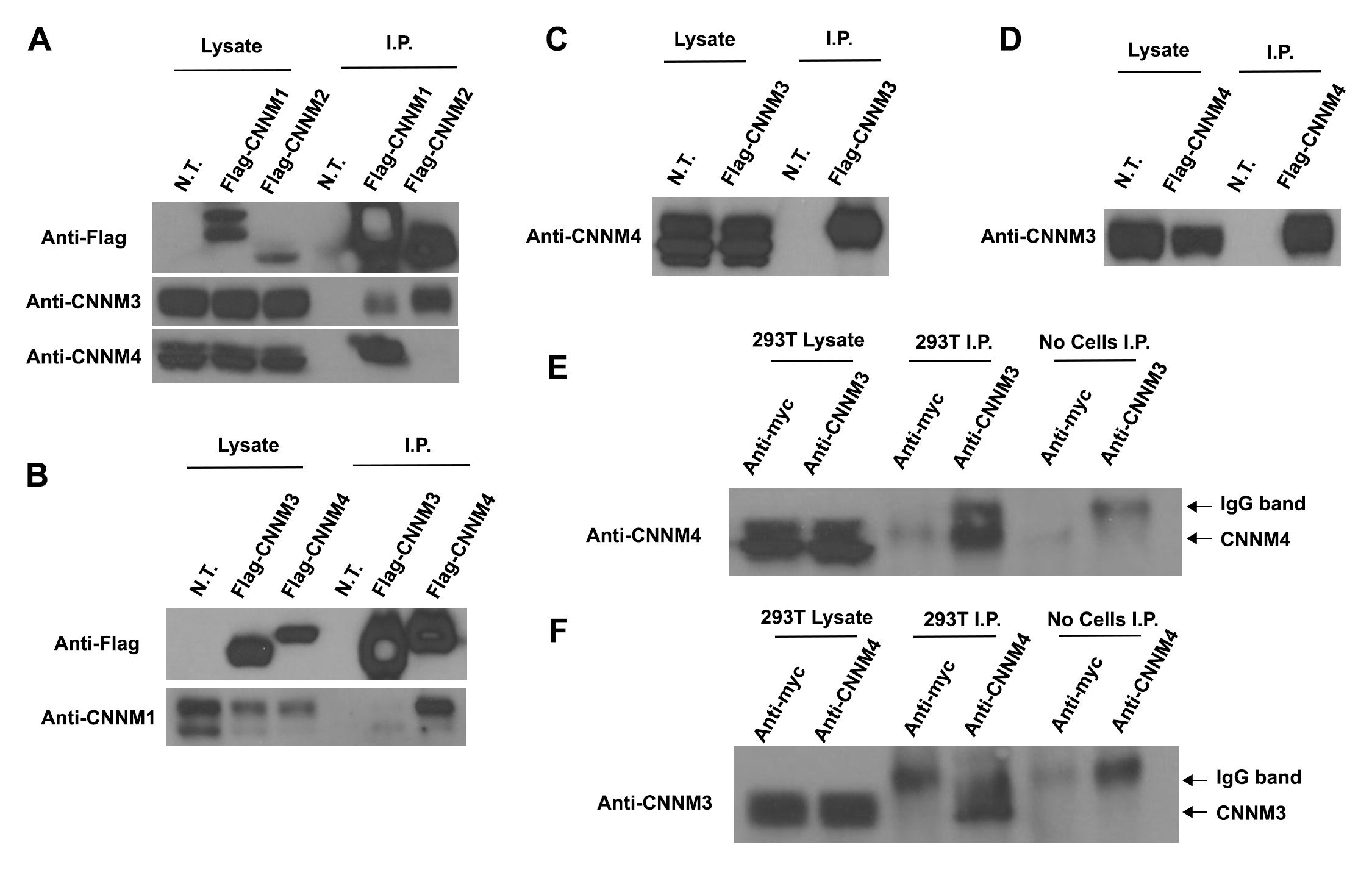

Supplement: S16 Fig — (A) FLAG-CNNM1 and FLAG-CNNM2 were individually expressed in HEK-293T cells. Immunoprecipitation of FLAG-CNNM1 effectively immunopurified native CNNM4, whereas native CNNM3 was immunopurified to a lesser degree. Immunoprecipitation of FLAG-CNNM2 immunopurified native CNNM3 but not native CNNM4. (B) FLAG-CNNM3 and FLAG-CNNM4 were individually expressed in HEK-293T cells. Immunoprecipitation of FLAG-CNNM4 efficiently immunopurified native CNNM1, whereas immunoprecipitation of FLAG-CNNM3 did not immunopurify native CNNM1. (C) FLAG-CNNM3 was expressed in HEK-293T cells. Immunoprecipitation of FLAG-CNNM3 was able to coimmunopurify native CNNM4. (D) FLAG-CNNM4 was expressed in HEK-293T cells. Immunoprecipitation of FLAG-CNNM4 was able to coimmunopurify native CNNM3. (E) Immunoprecipitation of native CNNM3 is able to coimmunopurify native CNNM4. Anti-myc antibody was used as a negative control for immunoprecipitation. Indicated is the nonspecific band derived from the antibody used for the immunoprecipitation. Below this band is CNNM4, as indicted by the arrow. (F) Immunoprecipitation of native CNNM4 is able to coimmunopurify native CNNM3. The anti-myc antibody was used as a negative control for immunoprecipitation. Indicated is the nonspecific band derived from the antibody used for the immunoprecipitation. Below the nonspecific band is CNNM3, as indicted by the arrow. Unprocessed images of blots are shown in S1 Raw Images. (TIF) [file pbio.3001496.s019.tif]
