# Supplementary material for: CNNM proteins selectively bind to the TRPM7 channel to stimulate divalent cation entry into cells
Source: PLoS Biol. 2021 Dec 20;19(12):e3001496. doi: 10.1371/journal.pbio.3001496 (PMC8726484; doi:10.1371/journal.pbio.3001496)
Supplement: Attachment — Submitted filename: ResponsetoReviewers_FINAL.pdf [file pbio.3001496.s021.pdf]

Response to Reviewers' Concerns

We are grateful to the reviewers for their helpful comments. Below is a point-by-point response to all of the reviewers' concerns, and a description of the changes we have made to the manuscript.

Reviewers' comments

Rev. 1:
The authors have made a significant effort in this revision and responded to many of the comments on the previous version. However, there still remain several problems that have not been sufficiently addressed or left uncorrected, which are explained below.

1: The experiments using 25Mg (Fig. 3A) clearly show that Mg2+ influx is stimulated by CNNM proteins. However, the critical point of this paper is whether it is ascribed to the "stimulation of TRPM7 activity by the protein-protein interaction with CNNM". The influx of 25Mg may just reflect the TRPM7 function independent of the interaction with CNNM proteins, for maintaining intracellular Mg2+ levels. The Na+ depletion experiments (Fig. 3E) show the increase in Mg2+ levels, even when Mg2+ efflux by CNNM proteins was probably suppressed. However, such Mg2+ increase can be caused by the reverse action of the ectopically expressed CNNM4 proteins under very abnormal condition. CNNM4 normally extrudes Mg2+ in exchange for Na+ influx, but in the absence of extracellular Na+, it can extrude Na+ in exchange for Mg2+ influx in the reverse action. In general, cells have approximately 10 mM intracellular Na+, of which efflux is theoretically sufficient for a few mM level increase in intracellular Mg2+. The authors' data show that Mg2+ increase by Na+ depletion was suppressed by NS8593 (TRPM7 inhibitor) and TRPM7 knockout, but I am wondering how these treatments could suppress the reverse action of the expressed CNNM4 proteins.
Related to this problem, I suggested the authors to use congenital disease-related CNNM mutants in the previous review comment. The authors actually tested the effect of one disease-related point mutant (CNNM2 T568I) and several deletion mutants. However, domain level deletions would certainly affect the protein structure, and the T568I mutation was reported to abolish the interaction with its ligand ATP (J. Biol. Chem. 289, 14731-14739, 2014), which affects the whole structure of the CNNM proteins (Sci. Adv. 7, eabe6140, 2021; Nat. Commun. 12, 4028, 2021). Therefore, these mutants may not be appropriate for discriminating Mg2+ influx function (via TRPM7) from Mg2+ efflux function (by itself). It should be noted here that there are several other point mutations in CNNM4, which are responsible for Jalili syndrome and abolish the Mg2+ efflux function, such as S196P and S200Y, ... (Sci. Adv. 7, eabe6140, 2021). In addition, recently reported structural data of its bacterial ortholog revealed that the residues are directly involved in the recognition of Mg2+ in the pore region formed in the membrane-spanning domain (Sci. Adv. 7, eabe6140, 2021). Mutations in these residues are expected to specifically affect the Mg2+ transporting function without so deleterious effects on the whole protein structure. In addition, the possibility of the reverse action of CNNM4 can be excluded using these mutants. The concept of Mg2+ influx function independent of Mg2+ efflux function is the core of this study, and thus, the authors should make the point clear by testing the effect of these congenital disease-related point mutants and showing the results.

We thank the reviewer for thinking deeply about the potential mechanisms by which CNNM's could potentially mediate divalent cation influx. It is suggested that in the absence of external Na^{+}, CNNM4 may operate in reverse, causing the increase of Mg^{2+} which account for the rise in intracellular Mg^{2+} when the external media is replaced with HBSS containing NMDG-Cl, as

shown in Figure 3B. The reviewer states, "I am wondering how these treatments could suppress the reverse action of the expressed CNNM4 proteins." Following the reviewer's logic as best we could, the only way we could imagine KO of TRPM7 or blockade of TRPM7 channel activity could stimulate the theoretical efflux activity of CNNMs in reverse would be to severely reduce the concentration of intracellular Na^{+}, which would interfere with Na^{+}/Mg^{2+} change. In our opinion, this scenario is highly unlikely to occur because intracellular Na^{+} would have to dependent on TRPM7 channel activity. However, this is also unlikely because extracellular Mg^{2+} largely blocks Na^{+}-influx through the channel and thus the absence of the channel from cells or transient inhibition of TRPM7 channel activity would be predicted to have at best only a negligible effect on intracellular Na^{+}. Nevertheless, we evaluated the effect of the several point mutations in CNNM4, which are responsible for Jalili syndrome and abolish CNNM Mg^{2+} efflux function (Sci. Adv. 7, eabe6140, 2021). We screened these mutants and discovered that the CNNM4-S196P mutant appears to suppress Mg^{2+}-efflux as previously reported yet does not interfere with Zn^{2+} influx stimulated by co-expression of the mutant with TRPM7. The other mutants appear to have partial effects on both activities, making them difficult to interpret. We include this data so the field can evaluate the results for themselves. Nevertheless, we have: 1) identified that the CNNM4-S196P mutant, which has been demonstrated to disrupt Mg^{2+} efflux, does not interfere with TRPM7-dependent Zn^{2+}-influx. 2) Shown that knockout of TRPM7 in HEK-293 (S6A) and HAP1 (S7A) suppresses divalent cation influx caused by overexpression of CNNNs. 3) Demonstrated that co-expression of CNNMs with the inactive pore mutant TRPM7-E1047K similarly suppressant divalent cation influx caused by CNNM overexpression, (S6E-H). And 4) Shown that transient application of the TRPM7 channel inhibitor NS8593 (S6D) suppresses divalent cation influx caused by co-expression of TRPM7 with CNNMs, all under conditions in which external Na^{+} concentrations are normal. We believe that the preponderance of evidence supports the hypothesis TRPM7 is required for CNNM-mediated divalent cation entry, bolstering the model that divalent cation influx stimulated by CNNMs is dependent upon the TRPM7 channel. We think that the CNNM4-S196P mutant will be useful in future studies and thank the reviewer for suggesting that we test the pore mutants.

2: The authors performed Mg2+ imaging analyses using Magfura2, but showed only the ratio values in the figure. As stated in the previous review comment, the authors should also perform appropriate calibration and show the calculated intracellular Mg2+ concentration, which is needed for proper evaluation of the data.

We worked diligently to calibrate the Mag-Fura-2 probe but were ultimately unsuccessful. This is most likely due to the fact it takes a very long time for the lower concentrations of free Mg^{2+} to reach equilibrium in HEK-293 cells when we permeabilize them with digitonin. We were requested after the first review to employ Mag-Fura2 so that we could "compare different samples." The new results obtained with Mg-Fura2 were in complete agreement with our previous experiments using the Mag-Fluo4 probe. The Mag-Fura2 data are presented as the ratio of the fluorescence emission from excitation at 340 versus 380, which is not uncommonly used in many publications to compare intracellular free magnesium levels (2018 Current biology, A Transient Rise in Free Mg^{2+} Ions Released from ATP-Mg Hydrolysis Contributes to Mitotic Chromosome Condensation – https://www.cell.com/current-biology/fulltext/S0960-98222(17)31668-8), 2016 Scientific Reports, Mitochondrial Mg^{2+} homeostasis decides cellular energy metabolism and vulnerability to stress. - https://www.nature.com/articles/srep30027). As these measurements are used solely to compare relative Mg^{2+}-levels between individual samples for mechanistic interpretation of experiments in which proteins are overexpressed in cells under non-physiological conditions, knowing the actual concentration of Mg^{2+} in the cells, while attractive, does not preclude interpretation of our experimental results. Thus, we present our final Mag-Fura-2 results as F(3400)/F(380), which are in agreement with earlier Mag-Fluo4

measurements, and hope the reviewers will understand that despite our best efforts we were not able to represent the imaging data in terms of absolute Mg^{2+} levels and that this does not take away from the conclusions of the experiment.

3: The newly added data of the localization of CNNM proteins (S11-S14) are not convincing. The authors use NHERF1 as an apical marker, but the signals do not show apical localization pattern in the presented data. In addition, localization of membrane proteins in epithelial cells are normally evaluated by vertical section images reconstituted from the stacks of confocal section images. Beautiful examples of apical localization of NHERF1 can be seen in the published papers (Mol. Membr. Biol. 18, 3-11, 2001; Am. J. Physiol. Renal Physiol. 311, F343-F351, 2016), in which the signals are observed only at the top side membrane in a large dot-like manner. In general, cell polarization requires careful handling of cell culture, and I suppose the cells did not polarize well enough for evaluating apical-basolateral localization. Normal localization of marker proteins is prerequisite for evaluating CNNM localization.
However, reading the authors' reply, I understand that further investigation in epithelial cells is not needed in this study. In that case, these preliminary data of expressed CNNM proteins and their descriptions in Discussion should be removed.

We have examined the data we presented and would agree that the NHERF1 immunofluorescence data taken of LLC-PK1 cells are not a "beautiful example" of apical NHERF1 localization. It's possible that these cells may not polarize well. However, the data we collected for OK cells, Caco-2 Cells, and RPTEC/TERT1 cells do show NHERF1 localization in a dot-like manner on the apical side reminiscent of the paper Reviewer 1 cites as well as one specific to OK cells (https://www.pnas.org/content/99/18/11957). However, not all cells show big punctate structures as observed in OK cells. For example, in Caco-2 cells the punctate structures usually appear smaller (e.g., see https://www.jbc.org/article/S0021-9258(20)62654-0/fulltext). In addition, we took care to make sure the cells had time to form the appropriate structures. For RPETC/TERT1 cells, we followed a protocol where we cultured cells for 1-2 weeks to let them form structures before imaging. Nevertheless, we admit that the images in the supplementary figure were small and may not have been large enough for the detail to be adequately evaluated by the reviewer. In our opinion the data from OK cells (S13 Fig), Caco-2 Cells (S14 Fig), and RPTEC/TERT1 (S15 Fig) were robust. We have thus provided improved zoomed-in images of the cells so that it is easier to visualize the NHERF1 localization in our revised supplementary figures. As we went to considerable effort to collect this data at Reviewer 1's request and as may be helpful to the scientific community and likely spark further research studies, our preference is to include the data from for OK cells, Caco-2 Cells, and RPTEC/TERT1 cells in our supplementary data.

4: Ectopically expressed membrane proteins often go to unusual locations. Therefore, localization data of SLC41A1 would be essential for appropriate evaluation of the results, as requested in the previous review comment.

We understand expressed membrane proteins often go to unusual locations, however, our intention was to use SLC41A1 simply a tool to lower intracellular magnesium as previously reported (Arjona FJ, Latta F, Mohammed SG, Thomassen M, van Wijk E, Bindels RJM, et al. SLC41A1 is essential for magnesium homeostasis in vivo. Pflugers Arch. 2019;471(6):845-60.). We were able to confirm the previous report and found that FLAG-SLC41A1 when overexpressed does lower intracellular Mg^{2+}. We now provide in the revised S8 Fig immunofluorescence data that shows that FLAG-SLC41A1 can be detected at cell border co-localized with CNNM3 and note in the text that when overexpressed FLAG-SLC41A1 can also be detected intracellularly.

5: In this revision, the authors added sequence information of the CNNM knockout cell lines used in this study (lines 540-547). Several frame-shift deletions in CNNM3 are described, but no information of CNNM4 is provided.

We thank the reviewer for discovering this error. There was a typo in the Methods section where "CNNM3" was written instead of "CNNM4". We have made the correction.

6: In the previous review comment, I listed several minor problems as "other minor points" at the last part. The authors answered properly to most of them, but some are left uncorrected. The following is the list of my comments on the original version and the authors' reply in the rebuttal letter, which are actually not corrected despite the authors' reply. The authors used ANOVA only for analyzing the results of Fig. 3A in this revision, but there are other data that need ANOVA (Figs. 1D, 2B, 2H, 3C, 3D, 4C, 4E, S2B, S8B, S8C, S8D, S9B, S10B, and S10F).

We apologize for missing some of the minor corrections. These have now been corrected. We have also reanalyzed the data for ANOVA as requested.

Line 25: Remove "it". This is from "Mg^{2+} it is the". It is now removed.
Line 244: "evaluate" – This is from "evaluation the impact". Changed to evaluate
Line 389: "were" resolved – This is from "the proteins resolved". "were" has been added.
Line 463: ANOVA should be used for statistical analyses. Also, two-tailed test would be appropriate. We have updated the statistical tests used in our analysis and state in the Methods section, "Unless otherwise indicated, statistical analysis used One-Way ANOVA plus Student's t-test (two-tailed). A p value of less than 0.05 was considered significant. Error bars indicate standard error."

Rev. 2:
The authors' have addressed my concerns. However, I strongly suggest to include their new negative data on the CNNM mutants (point 1 under Reviewer 1) with a short explanation why the experiments were done and a short discussion. While these data are negative, I feel it is important for the scientific community to know about negative results within the framework of a larger study such as this. It will provide context and likely spark further studies.

We thank Reviewer 2 for the careful review of our manuscript. As requested, we have included the requested data from our screen for mutants (CNNM2 T568I, CNNM2ΔCBS, CNNM4ΔCBS, CNNM4F631K and CNNM4ΔCNBH) that could separate CNNM-stimulated Mg^{2+}-efflux activity from CNNM-stimulated divalent cation influx activity. This data plus the new mutants we tested for Reviewer 1 in the last review have been incorporated into a new supplementary figure (S11 Fig). We have also added to the manuscript a short explanation of why the experiments were done.

Additional relevant literature that has been published since the original submission and mention any additional citations in your response.

It was requested by the Editors that we include additional relevant literature that has been published since the original submission. To keep the manuscript fresh, we have referenced the following papers in the revised manuscript as appropriate throughout the paper.

Huang Y, Jin F, Funato Y, Xu Z, Zhu W, Wang J, et al. Structural basis for the Mg(2+) recognition and regulation of the CorC Mg(2+) transporter. Sci Adv. 2021;7(7).

Chen YS, Kozlov G, Moeller BE, Rohaim A, Fakih R, Roux B, et al. Crystal structure of an archaeal CorB magnesium transporter. Nature communications. 2021;12(1):4028.

Zolotarov Y, Ma C, Gonzalez-Recio I, Hardy S, Franken GAC, Uetani N, et al. ARL15 modulates magnesium homeostasis through N-glycosylation of CNNMs. Cell Mol Life Sci. 2021.

Kollewe A, Chubanov V, Tseung FT, Haupt A, Müller CS, Bildl W, et al. The molecular appearance of native TRPM7 channel complexes identified by high-resolution proteomics. bioRxiv. 2021:2021.07.09.451738.

The last of these papers is a preprint on bioRxiv that reported that native TRPM7 channel complexes composed of TRPM7, CNNMs, PRL, and ARL15 can be detected in mouse and rat brain. This data is consistent with our own data, demonstrating that CNNM-TRPM7 complexes can be found *in vivo*. We also now include new data confirming these authors' discovery that ARL15 affect TRPM7 channel activity. S12 Fig shows that overexpression of ARL15 stimulates TRPM7-mediated Zn^{2+}-influx and that knockdown of ARL15 reduces TRPM7-mediated Zn^{2+}-influx. Thus, we have made efforts to keep our manuscript up-to-date and relevant to state of the field and think that the new additional data and citations will increase the impact of our paper on the research community.

Finally, we wish to again thank the reviewers and editors for their sustained interest and critical review of our manuscript, which has substantially improved the paper. It took a long time to get to this point, but we deeply appreciate the construct feedback that made our manuscript better.
