# Supplementary material for: CNNM proteins selectively bind to the TRPM7 channel to stimulate divalent cation entry into cells
Source: PLoS Biol. 2021 Dec 20;19(12):e3001496. doi: 10.1371/journal.pbio.3001496 (PMC8726484; doi:10.1371/journal.pbio.3001496)
Supplement: Attachment — Submitted filename: ResponseToReviewers_6_18_21.docx [file pbio.3001496.s020.docx]

**Response to Reviewers’ Concerns**

We are grateful to the reviewers for the many useful and helpful comments. The feedback has prompted us to conduct additional experiments that we feel have greatly improved our manuscript. Below is a point-by-point response to all of the reviewers' concerns, and a description of the changes we have made to the manuscript.

*****************************************************
REVIEWS:

Reviewer #1: In this manuscript, Bai et al. report a novel role of CNNM proteins in regulating TRPM7 cation channel. CNNM proteins are involved in Mg transport, but there has been a controversy over its precise function. The authors found that CNNM proteins bind to TRPM7 and stimulates its channel activity. TRPM7 is permeable to Mg and has been shown to play an important role in cellular and organismal Mg homeostasis. The authors claim that this study has revealed a crucial importance of CNNM proteins in regulating intracellular Mg levels by coordinating its direct role in Mg efflux and indirect role in Mg influx via TRPM7. However, the presented data are preliminary and not solid enough to support the authors' claim. In addition, there are many errors in the text. Substantial modifications and additional experiments will be needed to improve the quality of this manuscript to the level appropriate for publication in PLOS Biology. Major and minor problems are listed as
follows.

Major problems:
1: The authors claim that CNNM proteins have dual functions in both Mg efflux by themselves and Mg influx by stimulating TRPM7. The idea itself sounds very attractive because it can reconcile the controversy over the function of CNNM proteins. However, the presented data clearly show that CNNM expression decreases Mg levels irrespective of the TRPM7 expression status (Fig 3C). Therefore, Mg efflux seems to be the dominant function of CNNM proteins, even if Mg influx may also be stimulated. The authors need to show that CNNM actually stimulates Mg influx in the presence of TRPM7 to validate their claim. It should also be noted that there are serious problems in Mg imaging analyses, which were used to quantify Mg levels (see the following comment no. 2).
  Related to this issue, CNNM proteins are known to be associated with various congenital diseases, and several disease-related missense mutants have been shown to abolish Mg efflux activity. Using these mutant forms of CNNM proteins, the authors may be able to discriminate the activity for Mg influx via TRPM7 from that for Mg efflux. Experiments using such CNNM mutants are desirable.

There are four members of the CNNM family CNNM1-4. CNNM2 and CNNM4 appear to robustly mediate Mg^2+^ efflux, whereas CNNM1 and CNNM3 have less efflux activity (see Figure 1 from Hirata et. al. JBC 2014; <https://www.ncbi.nlm.nih.gov/pmc/articles/PMC4031528/>). CNNM3 in particular is associated with Mg^2+^-influx (See Fig 3, for example, from Hardy et al. Oncogene 2014; https://www.nature.com/articles/onc201433). Thus, CNNMs as a class have varied activity. Based on experiments we provided in our manuscript, we proposed that CNNMs can affect Mg^2+^-efflux as well as Mg^2+^-influx activities. To bolster our data, we conducted new experiments to analyze the effect of CNNM4, which has robust Mg^2+^-efflux activity, on Mg^2+^ influx. We now show using a ^25^Mg uptake assay (Revised Fig 3A) that CNNM4 stimulates TRPM7-mediated Mg^2+^-influx. Our experiment measured ^25^Mg uptake in WT HEK-293 cells, stable TRPM7 expressing HEK-293 cells, and stable TRPM7 expressing HEK-293 cells deficient in CNNM3 and CNNM4. As expected, in TRPM7 expressing cells ^25^Mg uptake is higher than in WT HEK-293 cells, in accordance with TRPM7 established ability to promote Mg^2+^ influx. Knockout of CNNM3 and CNNM4 from TRPM7 expressing HEK-293 cells reduced ^25^Mg to a level comparable to WT HEK-293 cells. Reexpression of CNNM4 in TRPM7 expressing HEK-293 cells deficient in CNNM3 and CNNM4 significantly recovered Mg^2+^ uptake, demonstrating the potential for CNNM4 to promote Mg^2+^-uptake in a TRPM7 dependent manner.

To add to the ^25^Mg data we also conducted experiments to further investigate the impact of CNNM4 on Mg^2+^ influx. We took advantage of work by Miki’s group, who demonstrated that CNNM4 overexpression induces Mg^2+^ efflux which depends on Na^+^/Mg^2+^ exchange (see Yamazaki et al. Plos Genetics 2013, Figure 4E; <https://journals.plos.org/plosgenetics/article?id=10.1371/journal.pgen.1003983>). Replacing NaCl in the extracellular buffer with NMDG-Cl was shown to block Mg^2+^ efflux by CNNM4, which provides us a special opportunity to separate CNNM4 influx function from efflux. In new experiments reported in Fig 3B we show that CNNM4 stimulates increases in intracellular Mg^2+^ levels in cells following change of the extracellular buffer from regular HBSS (with NaCl) to HBSS without extracellular Na^+^(with NMDG-Cl). In the absence of extracellular Na^+^, HEK-293 cells intracellular Mg^2+^ levels are similar to TRPM7 KO HEK-293 cells. Overexpression of CNNM4 in WT HEK-293 cells, however, causes a substantial increase in intracellular Mg^2+^ in the absence of extracellular Na^+^, whereas overexpression of CNNM4 in TRPM7 KO HEK-293 cells does not. We further show that the increase in intracellular Mg^2+^ levels in HEK-293 cells caused by CNNM4 overexpression can be blocked by the TRPM7 channel inhibitor NS8593. This new data demonstrates CNNM4 ability to mediate Mg^2+^-influx is dependent upon the native TRPM7 channel. Collectively, the ^25^Mg^2+^ and time-lapse CNNM4 Mg^2+^-imaging experiments support the Zn^2+^-imaging data (Fig 2) and electrophysiology experiments (Fig 4), demonstrating that CNNM4 driven Mg^2+^ uptake as well the influx of other divalent cations occurs in TRPM7-dependent manner. Thus, CNNMs have divalent-uptake activities, which depend on the TRPM7 channel. These new experiments, promoted by the suggestion of the reviewer, have strengthened our manuscript.

Reviewer 1 also suggested that it may be possible to exploit disease-related mutations in CNNM proteins to separate the discriminate the activity for Mg^2+^ influx via TRPM7 from that for Mg^2+^ efflux. This was an excellent idea. We evaluated several point and deletion mutants (CNNM2 T568I, CNNM2ΔCBS, CNNM4ΔCBS, CNNM4F631K and CNNM4ΔCNBH), known to disable Mg^2+^-efflux activity. All of these mutants also appeared to disable Zn^2+^-influx activity to varying degrees. This data indicates that the efflux and influx activities do not appear to be easily separable by mutation. Since we able to obtain new data separating CNNM4 efflux and influx data using Na^+^-free extracellular buffer (Fig 3B), we did not feel it necessary to incorporate our negative results into the manuscript, as it does not add to our understanding of how CNNMs affect TRPM7 function.

2: There are serious problems in imaging analyses for Zn and Mg. As for Zn imaging, the authors show only a single fluorescence image, which was obtained at 5-10 min after the 30 microM Zn supplementation, for each sample. However, it cannot be excluded that the results just reflect uneven incorporation of the fluorescent probe. In addition, time-lapse analyses before and after the Zn addition to the medium should be performed to quantify Zn influx into cells.
  Mg imaging experiments were done using Mag-Fluo4. It may be a good probe for time-lapse monitoring of the dynamic change in Mg levels in the same cell, but it is not ratiometric. Therefore, it cannot be used for quantifying absolute Mg levels, to compare different samples. For this purpose, the authors should use ratiometric probes for Mg with appropriate calibrations. Moreover, it should be noted that these Mg probes react not only with Mg but also strongly with Ca. TRPM7 channel is permeable to Ca, and thus, the results obtained by using fluorescent Mg probes need to be carefully interpreted. The authors should verify that the fluorescence signals in Mg imaging experiments properly reflect Mg levels, but not Ca levels, when analyzing TRPM7 functions.

We appreciate the reviewer’s concern about the Zn^2+^ imaging data. The fault comes the fact that we didn’t adequately describe the protocol in our Methods section and instead referenced a paper by Inoue and colleagues, where it was originally developed to study zinc-induced neurotoxicity by TRPM7 (Inoue et al. 2010. JBC). Since our original description of the methods was not extensive, it likely wasn’t clear to the reviewer how much care and effort we took to validate our assay. It took several months to develop the assay in our own lab, during which time we worked out the concentration of indicator to use in our experiments, the kinetics of Zn^2+^-uptake by TRPM7-expressing cells, and how to reduce variability when collecting data. We routinely visually inspect our cell samples to make sure there isn’t uneven loading of the dye and other artifacts, which in the end has resulted in an assay that is very reproducible, and produces results that can are comparable to other methodologies. Indeed, a testament to the validity the assay is the fact that we see similar experimental results using different approaches. The Zn^2+^-uptake assay mirrors the ^25^Mg-uptake assay, which parallels the electrophysiology results. Thus, we are confident in our original data and apologize for the poor description of our method in the Methods section. We have added more detail to our method description, so it is clearer how we did the experiments.

Nevertheless, as requested we conducted new time-lapse analysis experiments to bolster our original data. The Zinc-influx time-lapse assays allowed use to examine the kinetics of the changes in intracellular Zn^2+^-levels after incubating cells with 30 μM ZnCl^2^ for the different samples. It took a considerable amount of time and effort to conduct these experiments, which we are happy to report was entirely consistent with our original data. We now show time-lapse Zn^2+^-influx data to supplement our original experimental results; this new data appears in Fig 1F, Fig 2C & I, S6D&H Fig, S8F Fig, and S10D&H Fig. The new experiments validate our original results and greatly strengthens our manuscript by providing evidence of how CNNMs and TRPM7 affect the kinetics of Zn^2+^-influx into cells.

Reviewer 1 also had concerns of our use of Mag-Fluo4 and advised that we use Mag-Fura-2, which is a ratiometric probe, to compare changes in intracellular Mg^2+^ levels between samples. Unfortunately, a microscopy capable of doing these experiments was not available on our campus. We were therefore required to upgrade our Olympus IX70 microscope with a FURA filter set, including Dual exciter ER340ex – ET380ex – 400lpx4 – 510/80m, a filter cube, a filter wheel with internal shutter, and an upgraded filter/shutter control card installed into a refurbished LUDL controller system. These upgrades took 3 months to install during the pandemic and thus significantly contributed to the delay in the revision of our paper. We only add this to explain why it took us so long to revise our manuscript. We used our new ratiometric imaging system to use Mag-Fura-2 compare Mg^2+^ levels between different experimental samples. We are happy to report that the new data mirrored our original data, which was obtained using Mag-Fluo4. We have updated our methods section to describe the Mag-Fura-2 measurements and now report the additional data in an updated Fig 3D. We also applied ratiometric Mag-Fura imaging for our experiments with CNNM4 and SLC41A2 shown in S8C Fig, which reports that both CNNM4 and SLC41A2 similarly lower intracellular Mg^2+^ levels in 293-TRPM7 expressing cells, but that only CNNM4 stimulates an increase Zn^2+^-influx when co-expressed with channel (S8A,D&F).

The addition of the time-lapse Zn^2+^ ratiometric and time-lapse Mg^2+^ imaging experiments as well as Mag-Fura-2 imaging experiments considerably strengthened our paper by making our study more rigorous. We are grateful to the reviewer for the suggestions.

3: CNNM proteins are mainly expressed in polarized epithelial cells in vivo and localized at their basolateral membrane, whereas TRPM proteins are localized at the apical membrane. Therefore, they are secluded from each other in epithelial cells. It is uncertain whether the authors' conclusion based on the analyses using non-polarized culture cells can be generalized. The authors should perform experiments using epithelial cells and investigate the localization, complex formation, and functional relationship of CNNM and TRPM7.

There is a misconception that CNNM proteins are mainly expressed in tissues with polarized cells, such as kidney and intestine. In fact, CNNM isoforms can be found in nearly all tissues, with CNNM3 being the most widely expressed isoform. For example, CNNM4 is expressed in liver, where has been recently found to have a role in hepatic transport of Mg^2+^(Simon et al. 2014. J. of Hepatology). CNNM3 and CNNM4 in particular are highly expressed in cancer cells (Hardy et al. 2015. Oncogene; Funato et al. J. Clin Investigation. 2014). Thus, CNNMS are an important class of proteins expected to have important functions in non-polarized as well as polarized cells as well as in physiological as well as pathological states. Whether CNNM and TRPM7 form a complex to mediate divalent cation influx in polarized cells is a fascinating question. However, we feel that it is beyond the scope of the present paper as it is an experimental question that will take substantial time and effort to answer. Nevertheless, we did conduct experiments to address the reviewer’s question about whether CNNMs have the potential to localize to apical membranes in polarized epithelial cells where it could potentially form a complex with TRPM7. In OK proximal tubule epithelial cells, for example, transiently expressed CNNM1-4 can be detected on apical membranes to varying degrees, with CNNM2 found more concentrated on apical membranes than basolateral membranes (new S11 Fig). Whereas CNNM4, in particular, is more concentrated at the basolateral rather than apical membranes. CNNM1 and CNNM3 can be found in both apical and basolateral membrane in OK cells (S11 Fig). We also conducted an analysis of the localization of FLAG-CNNM2 and FLAG-CNNM4 in other polarized cells, including CaCO2 (new S12 Fig), immortalized primary renal proximal tubule epithelial cells (S13 Fig), and LLC-PK (S1*4* Fig), where we find that to different degrees CNNM2 and CNNM4 can localize to both apical and basolateral membranes, with again CNNM2 found more prominently found on apical membranes and CNNM4 found more concentrated on basolateral membranes. The collection of these results suggests that CNNMs and TRPM7 are likely not be secluded from each other in polarized epithelial cells. We have added a discussion of this question to the Discussion section of the manuscript. However, a full analysis of the function and localization of CNNM and TRPM7 in polarized epithelial cells, while intellectually compelling, is beyond the scope of the present study and will be its own individual research project.

Northern blot of mouse CNNM expression. Figure taken from Wang et al. 2004. BMC Genomics.

4: The authors show the data of TRPM7-CNNM complex formation using culture cells ectopically expressing TRPM7. They should investigate whether endogenous TRPM7 proteins associate with endogenous CNNM proteins. Also, it should be addressed whether the complex formation status is affected by PRL expression or Mg availability, such as Mg depletion from the medium and Mg supplementation.

The reviewer is correct that in the original draft of our paper we primarily showed evidence of TRPM7-CNNM complex formation in HEK-293 cells ectopically expressing TRPM7. We have conducted new experiments showing ectopically expressed TRPM7 can interact with native CNNM3 and/or CNNM4 to form complexes in other cell types, including Hela, OK, HAP1, and immortalized primary proximal tubule epithelial cells (S3A-D Fig). Despite the difficulty in doing biochemistry experiments with the native channel, owing to TRPM7 extremely low expression level, we were also successful in demonstrating that immunoprecipitation of the native TRPM7 channel from the ZR-75 -1 breast cancer line can co-immunoprecipitate native CNNM4 (S3E Fig). Thus, we have added evidence that CNNM-TRPM7 complexes can be detected in different cell types and that the native proteins can be found as a complex in ZR-75-1 cells.

Additionally, we also conducted experiments to test whether CNNM’s can affect the endogenous channel activity of native TRPM7. As TRPM7 has been shown by us and others to be the predominant source for the TRPM7-like current in HEK-293 cells (Su et al. JBC. 2006; Abira et al. PNAS. 2017), we took advantage of this system by knocking out both CNNM3 and CNNM4 in WT HEK-293 cells. Loss of both CNNM3 and CNNM4 significantly reduced the native TRPM7 current amplitidudes, providing evidence that native CNNM3 and CNNM4 impact the function of the native TRPM7 channel. This new data is shown Figure 4F&G. The new results mirror what we observed with the overexpressed channel in 293-TRPM7 cells and demonstrate that the native channel can also be affected by endogenous CNNM proteins.

We think it is a very interesting question whether CNNM-TRPM7 complex formation is affected by Mg^2+^ availability. In the experiments we have conducted thus far we have not observed any obvious effect on complex formation in response changes in Mg^2+^ availability. We also did experiments to test whether overexpression of PRLs with CNNM and TRPM7 affect co-IP of CNNMs and TRPM7 and we did not observe marked change in the stability of the complex. Since the issue of how CNNM-TRPM7 complexes are regulated by changes in Mg^2+^ status is an interesting one, we have added to the Discussion section of the manuscript a discussion of this question and cite recent papers reporting how changes in intracellular Mg^2+^ affect TRPM7 and PRL expression levels, which could be a unique mechanism by which the of CNNM-TRPM7 complexes are controlled *in vivo*.

Minor problems:
Fig 1B: The estimated size of the bands in the IP samples seems to be larger than that in the lysate in the same gel.

We are assuming that the reviewer is referring to the migration of the native CNNM3 and CNNM4 bands in the I.P. samples. There is considerably more CNNM3 and CNNM4 in the lysate of the blot, which makes the band larger. If you compare the upper size of the bands the migration is similar, so we believe that the difference in sizes observed are likely due to sample loading in this blot. It’s worth noting, however, that Western Blotting of HEK-293 cell lysates using the anti-CNNM4 often reveals two bands, with the upper one being the bona fide CNNM4 (e.g., see Fig 2D where the lower band still remains in HEK-293 CNNM4 and CNNM3/4 KO cells). It is also possible that the CNNM3 band in the I.P. lane is migrating slightly higher in the lysate and that this reflects some sort of post-translation modification. This is something we are currently investigating and will pursue in future work.

Fig 1C: The authors claim that the expressed proteins co-localize at the cell border. However, the cells have become rounded and the most part is occupied by the nuclei. Therefore, it is difficult to determine the precise localization of these proteins based on the immunofluorescent images shown here. Ectopically expressed membrane proteins often aggregate intracellularly, and thus, more careful investigations, such as co-staining with plasma membrane markers with appropriate controls and quantifications, are needed.

We agree that the image we provided in the original Fig 1 was far from ideal. We carefully repeated the experiment and provide new images of TRPM7 and CNNM3 in HEK-293 cells (S5 Fig). Our analysis of the localization of CNNM3 is that this protein primarily localizes to the plasma membrane, where it colocalizes with the plasma membrane marker Na^+^/K^+^-ATPase. TRPM7, on the other hand, can often found intracellularly in the E.R. in HEK-293 cells, as we reported in a previous publication (Overton et al., *Scientific Reports.* 2015), as well as at the plasma membrane. We have repeated the experiments and show improved co-staining images that show areas of co-overlap at the border of HEK-293 cells. We have changed the language to reflect that the two proteins can be detected at the plasma membrane.

Fig 1E: The information of the anti-TRPM7 antibody should be provided in the methods section.

We have revised the figure legend to indicate the antibody used to detect TRPM7 was the TRPM7-specific PLIKC47 antibody (anti-C47) antibody we previously characterized. A mouse monoclonal antibody against TRPM7 from Alomone is also now used in the revised manuscript, and this has been added to the Methods section as well.

Fig 2C: The CNNM4-immunoblotting result shows irregular bands in CNNM3&4-KO cells and CNNM3-KO cells. What do these signals represent? Furthermore, the authors performed immunoblotting analyses to verify these KO cells, but the changes at the genome level should be directly confirmed by DNA sequencing around the CRISPR-Cas9 target site and shown in the figure.

The CNNM4 antibody recognizes a non-specific band, which is now indicated in the revised figure legend for Fig 2D. We also have performed DNA sequencing around the CRISPR-Cas9 target sites for the cell lines. We now report the changes at the genome level in the cells lines we created in the Methods.

Fig 2E: The result is not clear enough to justify the authors' claim. In addition, no data for validating the specific labeling of membrane proteins are presented.

We reperformed the experiment using a different approach to validate labeling of TRPM7 and not a control cytosolic protein (Tubulin). The new data is shown in a revised Fig 2E.

Fig 2F-H: Experiments using PRL-2 overexpression need appropriate controls. The results of R107E mutant shown in S9 fig should be moved here.

Since we now include time-course measurements in Fig 2 for two separate Zn-influx assays we do not have the space to provide this control in the main Figure, which itself had its own additional Zn-influx kinetics assay done to accompany the data. The revised (S10 Fig) contains this plus additional data to show the specificity of PRL stimulating TRPM7 channel function.

Fig 2G: The information of the anti-PRL-2 antibody should be provided in the methods section.

We now provide the information about the anti-PRL-2 antibody.

Fig 3A: The authors should perform appropriate statistical analyses to validate the claim.

Appropriate statistical analyses is now provided for Figure 3A.

S1 Table: Many candidate proteins are identified in MS analyses. The authors should explain the reason why they chose CNNM proteins for further analyses. Also, experimental details of MS analyses should also be described.

We apologize for our oversight in not providing a detailed description of this experiment. We have revised the results section to explain our rationale for why we chose CNNM proteins for further analysis. We have added to the Methods section a full description of the experimental details of the mass spectrometry experimental analysis.

S7 fig: Immunoblotting and immunofluorescence data for SLC41A1 should be shown in the figure to verify its proper expression and localization at the plasma membrane.

We now provide immunoblotting data for SLC41A1 to show that it is expressesed in HEK-293. We also provide new functional data to verify that it mediates intracellular Mg^2+^ extrusion as previously reported (Arjona FJ, Latta F, Mohammed SG, Thomassen M, van Wijk E, Bindels RJM, et al. SLC41A1 is essential for magnesium homeostasis in vivo. Pflugers Arch. 2019;471(6):845-60.) This new data has been incorporated into S8 Fig.

Other minor errors:
Line 25: Remove "it". -- removed
Line 133: D and E should be E and F, respectively. – with the new data this has been revised as d-g
Line 147: Fig 1D should be Fig 1E. – This has been corrected.
Line 148: overexpression "of" either – This has been corrected.
Line 168: S7 fig 6 should be S7 fig B. – This has been corrected
Line 174: Fig 1C,E should be Fig 1D,F. – This has been corrected.
Line 179: Remove "Fig 2A,B". – This text has been removed.
Line 244: "evaluate" – This text is now “evaluated”
Line 251: Fig 3C should also be referred here. We have added text referring to these experiments in this section.
Line 283: The voltage information should be indicated. The voltage information is indicated in the methods section.
Line 389: "were" resolved – “were” was added.
Line 443: Experimental details of the ICP-MS analyses should be described. -We have added a new section to the Methods sections that describes the experimental details of the ICP-MS analysis
Line 454: transfection "of". – “of” is now included.
Line 463: ANOVA should be used for statistical analyses. Also, two-tailed test would be appropriate. We have updated the statistical tests used in our analysis.
Line 674: "LacZ" in the figure should be mentioned in the legend. - LacZ has now been included.


Reviewer #2: The manuscript provides convincing data supporting the hypothesis that CNNM proteins are regulatory/accessory proteins enhancing divalent ion flux through the channel domain of the TRPM7 bifunctional protein. In the absence of TRPM7, CNNMs carry an important magnesium efflux role.

While the data are comprehensive and logically presented, major confusion remains in regards to the TRPM7 species, antibody tag and expression system used. For example, it seems that three different overexpression systems were used for TRPM7 alone, and all in HEK293 cells: FLAG tag (identified as mouse TRPM7, tet-induction), HA tag (species not identified, also not described in methods as of source etc. Human?), and a third unspecified "stably overexpressing TRPM7" cell line, also not described in the methods (line 141 and beyond). What was the tag, source and overexpression system (tetracycline? Other?) of the "stably overexpressing TRPM7" system? In most cases, TRPM7 overexpression leads to cell detachment and cell loss. How did the authors counteract that in this cell line? The above has to be clarified in order to substantiate the conclusions drawn.

We apologize for the confusion caused by using the multiple HEK-293 cell lines for our study. We did not do a good job in describing these reagents in the Methods section or throughout the results section. For a majority of the studies we employed the LTRPC7 cell line which overexpresses mouse FLAG-tagged TRPM7. This cell line was used for the mass spectrometry experiments as well as for nearly all of the functional studies (Zn^2+^, Mg^2+^, and electrophysiology) with the exception of S6 Fig, where we used HEK-293 cells expressing HA-tagged TRPM7 and HA-tagged TRPM7-E1047K so that experiment has its own positive control. For the co-immunoprecipitation experiments involving mouse HA-tagged TRPM7, the plasmid was transiently transfected into HEK-293T cells.

The reviewer is correct that TRPM7 overexpression in the LTRPC7 cell line in particular can lead to cell detachment and cell loss when the cells are plated on tissue culture plates and allowed to express for over 24 hours. We were able to overcome this limitation by plating the cells on polylysine coated plates and not indicate this in the Methods section.

To avoid any confusion we have updated the Results and Methods sections to make it clear what specific cells were used for the different experiment and that we are employing polylysine to keep cells attached to cell culture plates and coverslips in our imaging experiments.

The single point mutation in the TRPM7 channel domain is cited from previous work by the authors, however, it would be helpful to again mention the source and species and expression system in the methods.

As described above, we have updated the results and Methods section to better describe the source, species, and expression system used for these experiments.

S5B (described line 151 and beyond) clearly shows a doubling in Zn influx in the absence of TRPM7 and overexpression of CNNM2 compared to WT alone. Is this statistically significant? This is in contrast to what is seen in HAP1 cells (S6). The text guides the reader away from this observation and instead provides a lump sum statement that endogenous TRPM7 is required for Zn influx. This might be the case for the HAP1 cells, but in HEK293 the picture might be more differentiated. Perhaps worth briefly discussing (if statistical significance is observed in HEK293)?

The reviewer noted that CNNM2 overexpression in TRPM7 KO HEK-293 and TRPM7 KO HAP1 cells slightly increased Zn^2+^ influx compared to non-expressing TRPM7 KO HEK-293 and HAP1 expressing cells. Yes, in both HEK cell and HAP1 cells, the current data showed a significant difference, which might indicate that TRPM7 is responsible for most but not all CNNM2 overexpression induced Zn^2+^ influx. Others proteins such as TRPM6 or a zinc transporter, etc may be mediating this effect. These results, while interesting, are not the focus of the paper. To fully and seriously explore this effect, more extensive study is needed. Thus we changed the language and instead of saying that TRPM7 is “required” for CNNM mediated divalent cation influx that the channel instead is the major contributor to this effect, which leaves open that other channels (e.g., TRPM6) may also be affecting CNNM-mediated influx.

The primer sequences for the Turbo ID experiments need to be provided in the Supplemental Data (line 352).

We now include primer sequences for all of our constructs in the Methods section.


Reviewer #3: In the present paper, Runnels and colleagues identify and characterize CNNM proteins as modulators of TRPM7 function and propose the concept that CNNMs may serve as regulatory subunits of TRPM7 channel complexes in the plasma membrane to fine-tune cellular magnesium entry via TRPM7.

In principle, these are very interesting observations. However, there are a number of critical issues that require further attention.

Conceptual shortcomings:

(1) The physiological relevance of the results is rather limited. In particular, the study mostly relies on proteomic data and functional experiments conducted with cultured cell lines (HEK293) and - with few exceptions - overexpressed proteins. Hence, it remains unknown whether TRPM7 interacts with CNNM and RPL proteins in native tissues.

We agree that our data presented mainly focused on the overexpressed channel and the relevance of the interaction of TRPM7 channel to the native system is limited. We now provide new data demonstrating whether the activity of the native channel is affected by loss of CNNM proteins. In experiments added to Figure 4F&G we now report that knockout of *CNNM3* and *CNNM4* in HEK-293 cells reduces the activity of the native TRPM7 channel. We also present new biochemical data (S3 Fig) that CNNM-TRPM7 complexes can be observed in different cell systems, including Hela, HAP1, and OK cells, and RPTEC cells, which is immortalized primary rental proximal tubule epithelial cells. Finally, we provide new data that the native complex can be detected in ZR-75-1 breast cancer cells (S3E Fig). Elucidating the function and regulation of native CNNM-TRPM7 complexes *in vivo* is the next step in our research and we look forward to continuing this work and sharing the results in future publications.

(2) Mechanistically, the regulatory role of CNNM proteins for TRPM7 activity has been clearly defined. Several important questions remain to be addressed. Thus, it remains elusive which channel characteristics of TRPM7 are affected by CNNM or RPL proteins (open probability, kinetics of activation/rundown, sensitivity to PIP2, Mg2+ and ATP etc.). Does TRPM7 interact with CNNMs transiently or are stable complexes formed? What is the stoichiometry of such heteromeric complexes? Which domains of TRPM7 and CNNM proteins are required for the interaction to occur?

The reviewer asks very good questions and these are issues that we are keenly interested in. Our immunoprecipitation data supports that CNNM and TRPM7 form stable complexes. The stoichiometry of this complex is not unknown and is a challenging question to answer. The effect of CNNM or PRL proteins on kinetics of activation, sensitivity to PIP_2_, Mg^2+^, and ATP, are things are currently investigating and will take many years to complete. Thus, these experiments, while important, are beyond the scope of the current study.

(3) The dominating theme of the Abstract, Introduction and Discussion sections is cellular Mg2+ homeostasis and possible implications of CNNM/RPL proteins in Mg2+ transport. In this regard, there is a kind of disconnect with the actual experiments performed mostly concentrating on biochemical verification of proteomic data, imaging of free Zn2+ levels and patch-clamp assessment of outward monovalent cation currents of TRPM7. As the TRPM7 channel plays a central physiological role in the transport of Zn2+ and Ca2+ (in vitro and in vivo), it appears to be somewhat premature and unjustified to claim that the experiments shown are mainly relevant for Mg2+ homeostasis.

We have softened the language throughout the manuscript claiming that the experiments shown are mainly relevant for Mg^2+^ homeostasis and agree with the reviewer that our findings are likely to be relevant to the regulation of Ca^2+^ and Zn^2+^ homeostasis through the function of the TRPM7 channel. Our emphasis on Mg^2+^ in the writing of the manuscript is because we were motivated to address the controversy over the dual function of the CNNMs, which is one of the key points of the paper.

(4) Nearly all the data shown in the manuscript indicate that CNNM/RPL proteins are not essential (required) TRPM7 function per se. For instance, Fig. 5 clearly shows that TRPM7 currents were only reduced in the absence of CNNM3 and CNNM4. Therefore, the authors should avoid claims that CNNN proteins are 'required' for or 'control' the TRPM7 channel. It would be more appropriate to use terms like 'regulate' or 'modulate'.

We agree that we should have more careful with our choice of language. We have changed the language in the manuscript as suggested throughout the manuscript. Thank you for the excellent suggestion.

(5) The author correctly point out that most of the intracellular Mg2+ is bound to ATP and other metabolites and that free Mg2+ accounts for only ~10% of total Mg2+ levels. It remains unclear how to correlate the reported alterations in Mag-Fluor4 fluorescence and 25Mg2+ uptake with particular changes either in free Mg2+ levels or in total Mg2+ content of the cell. The authors regularly claim in the text that CNNM/RPL are central for cellular Mg2+ homeostasis. To this end, the authors have to provide direct evidence supporting such a conclusion.

When we were stating that CNNM and PRL “are central for cellular Mg^2+^ homeostasis” we were referring to other published work. This is not something we are claiming based on data we are reporting in the present manuscript. Nevertheless, we have revised the manuscript so it’s clear that we are not making this conclusion ourselves but instead referencing other existing research.

(6) The most prominent phenotype of TRPM7-deficient cells is reduced total Mg2+ levels entailing a Mg2+-dependent proliferation block. The authors created CNNM3 and CNNM4 gene-deficient clones which are important and valuable reagents to demonstrate the physiological importance of these proteins. If CNNM proteins are indeed required for TRPM7-mediated Mg2+ uptake, CNNM-deficient cells should somehow mirror the TRPM7 null phenotype. However, the mutant cells appear to grow normally under standard cell culture conditions (the authors did not explicitly state that extra Mg2+ has to be added to the culture medium). These results have to be transparently reported and discussed accordingly.

As demonstrate in our manuscript, CNNMs have dual activities, affecting both Mg^2+^-efflux as well as divalent cation uptake. Therefore, knockout of CNNMs would not necessarily be predicted to produce the same cellular phenotype observed with knockout of TRPM7. The cell lines we created in this study do not require Mg^2+^ supplementation for external cell growth. We now report this fact in our Results section as well as in the Methods sections where we describe the cell lines. A detailed study of how the CNNM-TRPM7 influx and CNNM efflux activities are simultaneously influenced to affect to cellular magnesium homeostasis will be conducted and reported in future work.

Methodological shortcomings:

(1) TRPM7 proteome.
Proteomic procedures are not described at all. As gleaned from the short text in the footnote of Table S1, it appears that FLAG-tagged TRPM7 was pulled down using FLAG-specific antibodies in HEK293 cells overexpressing the recombinant protein. The authors have to provide a detailed and fully transparent protocol for all steps of the experiment, including cell lysis conditions, immunoprecipitation, and assessment of the yield and purity of enriched TRPM7 proteins. What was the negative control to rule out unspecific hits? How exactly were raw MS data analysed? What were the biological and technical replicates of these experiments and how as statistical analysis of the data done? How were proteomic data sets assessed qualitatively and quantitatively and what exactly were the criteria and the statistical procedure to identify hits? The authors should explain what is exactly depicted in the '293-TRPM7 peptide count' and '293- peptide count' columns of Table S1. Which proteins
are considered as interaction partners of TRPM7? Most proteins are abundantly present in both columns. Are they considered as false-positive hits? What was the ratio of positive and unspecific hits? CNNM3 and CNNM4 proteins are represented by a relatively low frequency of peptides detected. How does this finding support the hypothesis that that CNNMs are relevant interaction partners of TRPM7? Finally, fully annotated proteome results have to be submitted to a publicly accessable database available for reviewers and the scientific community.

Rather than give a long description of our mass spectrometry experiment in our original submission of the manuscript we only referenced a previous publication (Cai et al., Scientific Reports, 2017), where we reported mass spectrometry experiments describing our analysis of TRPM7 phosphorylation. This was a major error in not including a fully description of our methodology and we apologize for our oversight. We now include in the Methods section a full description of the mass spectrometry approach we used to first identify CNNM proteins as potential TRPM7 interacting proteins. Two biological replicates were performed in our experiments. In the original submission we only showed the raw data. We have worked with our mass spectrometry facility to prepare a new supplementary table where we show proteins that were detected in both experimental samples but not present in the negative control samples. Thus, CNNMs were one of the few proteins that were identified as potential TRPM7 interacting proteins after this process. Among the other proteins identified in our analysis were ARL15 and PTP4A1, which are CNNM-interacting proteins. Together this data supports our decision to conduct additional experiments to explore the potential interaction between TRPM7 and CNNMs. Finally, we have worked with our mass spectrometry facility to submit the full data sets to a public database. We were instructed by the database to include the following information so that you can review the data submission.

Submission details:

**Project Name:** CNNM Proteins Regulate the TRPM7 Channel to Control Cellular Divalent Cation Entry

**Project accession:** PXD026635

**Project DOI:** Not applicable

Reviewer account details:

**Username:** reviewer_pxd026635@ebi.ac.uk

**Password:** luawCt5Y

(2) IP-based assessment of TRPM7-CNNM interaction.
Many methodological issues are not described. The authors should report all procedures and reagents used for IP experiments. Also, please explain what kind of negative control is shown in Fig. 1A? Presumably, HA-TRPM7 protein was not expressed in HEK293 cells, but the corresponding figure legend does not clearly explain this point. As CNNM proteins are transmembrane proteins, incomplete solubilisation of the membrane fraction is a common limitation of IP approaches in general, i.e. pull-down of TRPM7-containing vesicles together with CNNM proteins may occur. How was the quality of the membrane solubilisation assessed for the experiments shown in Fig. 1?

We apologize for omitting a description of our immunoprecipitation methods. The Methods section contains a detailed description of the procedures used, including a description of the solubilization procedure we employed. A description of how solubilization was originally assessed is now provided. We have revised the Figure legend for Fig 1A and S1 Fig to indicate that the negative control was non-transfected HEK-293T.

(3) 'Zinc influx' assay.
In the manuscript Zn2+ imaging experiments reflect the main approach to identify the functional role of CNNM proteins. However, this is the weakest aspect of the manuscript. In many figures (Fig. 1D, 2D, 3D, 4C, S3, S5-S9), the authors used whole-frame images captured at only one time point to measure intracellular Fluo-Zin-3 fluorescence and described this approach as a 'zinc influx' assay. At best, such images can rather crudely report on the steady-state level of free cytosolic Zn2+, but cannot be interpreted as dynamic 'zinc- influx'. Thus, transient expression of CNNM proteins may affect efflux of cytosolic Zn2+ or release of Zn2+ from intracellular compartments (free Zn2+ accounts for only ~1% of the total cellular Zn2+ content). In addition, overexpression of TRPM7 or CNNMs may influence the loading efficiency of Fluo-Zin-3 rather than free Zn2+ levels. Furthermore, the authors randomly picked up cells to quantify the Fluo-Zin-3 fluorescence, but it is unlikely that
CNNM cDNAs were transiently transcribed in 100% of cells. How would Fluo-Zin-3 fluorescence look like in un-transfected cells on the same dish? The conventional approach to conduct such dynamic influx studies is based on single-cell imaging over time to also work out the kinetics of changes of Fluo-Zin-3 fluorescence in transfected cells. Also, calibration of Zin-3 fluorescence against reference concentrations of Zn2+ is common practice to verify the physiological impact of alterations of Zin-3 fluorescence and to exclude numerous potential artefacts like movement of cells, bleaching of the probe, and reduced loading of the probe. TRPM7 blockers should be used to demonstrate that changes of Zn2+ uptake are indeed due to increased TRPM7 channel activity. Hence, the author's claim that CNNMs regulate TRPM7-mediated Zn2+ influx is not convincingly supported by the data shown.

As described above in our response to Reviewer 1, we should have done a much better job in the original manuscript describing the details of our Zn^2+^-influx assay. We are well aware of the potential of artifacts, such as bleaching of probe and reduced loading of the probe and we do not thing that these were factors influencing the original data. We were very careful in obtaining the original data. Nevertheless, as described to the response to Reviewer 1 we have now conducted experiments to show the dynamics of Zn^2+^-influx in response to CNNMs. This new data supports our conclusions from the original experiments. We have also added new data showing that influx of ^25^Mg mirrors our data with Zn^2+^ (Fig 3E), which is blocked by the TRPM7 channel inhibitor NS8593 as well as knockout of TRPM7. Also, we conducted experiments to show that the changes in Zn^2+^ uptake are due to TRPM7 channel activity by conducting experiments using the TRPM7 channel inhibitor NS8593 and that this activity is not observed in TRPM7 knockout cells (S6 Fig).

(4) Mag-Fluo4 imaging.
As explained above for experiments with Fluo-Zin-3, single snapshots of cells loaded with Mag-Fluor4 do not provide meaningful mechanistic information about the functional role of CNNMs.

As described above in the response to Reviewer 1 we have repeated all experiments using the ratiometric Mg^2+^-indicator Mag-Fura-2. The new data mirrors the original data taken with Mag-Fluor-4, thus confirming its validity (Fig 3C&D and S8B&C Fig) . We also provide new time-course measurements using Mag-Fluo4 to observe Mg^2+^-influx (Fig 3E), which further strengthens our conclusion that CNNMs are capable of mediating Mg^2+^-influx through TRPM7.

In summary, we believe that the new experiments have greatly strengthened the manuscript and are extremely grateful to all the reviewers for their constructive suggestions.
